# Weakened weekdays: lockdown disrupts the weekly cycle of risk tolerance

**DOI:** 10.1038/s41598-023-48395-9

**Published:** 2023-11-30

**Authors:** Virginia Fedrigo, Benno Guenther, Rob Jenkins, Matteo M. Galizzi, Jet G. Sanders

**Affiliations:** 1 Department of Psychological and Behavioural Sciences, London School of Economics and Political Science, Houghton Street, London, WC2A 2AE UK; 2 Department of Psychology, University of York, Heslington, York, YO10 5DD UK

**Keywords:** Psychology, Human behaviour

## Abstract

Risk tolerance decreases from Monday to Thursday and increases on Friday. Antecedents of this weekly risk cycle are difficult to investigate experimentally as manipulating the seven-day cycle is impractical. Here we used temporal disorientation during the UK COVID-19 lockdown to conduct a natural experiment. In two studies, we measured responses to risk in participants with either a strong or weak sense of weekday, after either a short or long period of disruption to their weekly routine by lockdown. In Study 1 (N = 864), the weekly risk cycle was consistent in risk attitude measures specifically to participants who reported a strong sense of weekday. In Study 2 (N = 829), the weekly risk cycle was abolished, even for participants who retained a strong sense of weekday. We propose that two factors sustain the weekly risk cycle. If the sense of weekday is lacking, then weekday will have little effect because the current day is not salient. If weekday associations decay, then weekday will have little effect because the current day is not meaningful. The weekly risk cycle is strong and consistent when (i) sense of weekday is robust and (ii) weekday associations are maintained.

## Introduction

Does the day on which a decision is made affect the outcome of the decision? On its face, it seems unlikely. The day of the week is rarely a factor in decision making. However, weekdays have distinct profiles at the level of mental representation^1,2^, are associated with different routines and activities^3^, and can arouse contrasting emotional states^4–6^.

Weekly fluctuations have been well documented in a variety of settings. Examples range from traffic flow^7^ and energy consumption^8^ to medical^9–11^, economic^12^, and political decisions^13^. For example, one study suggests that opting for a surgery later in the week can double the risk of complications^9^. Another study shows that the day on which national votes are held could determine their outcome^13^. As counterintuitive as it may seem, our adherence to the weekly cycle has unintended consequences in all sectors of society.

Why do weekly fluctuations in decisions outcomes occur, and why are they so widespread? At a higher level, insights from personality psychology can shed light on a speculative mechanism. Past work has shown that individuals behave in different ways, especially in manifestation of different personality traits, in different settings^14,15^. As such, each day of the week can be conceptualised as a different stereotyped ‘setting’, wherein the norms and expectations (i.e., one attends a pub in the UK on a Friday or Saturday at more than 4 times the rate as on a Monday^16^) dynamically shape the manifestations of different traits.

One possible explanation is that the weekly cycle affects a foundational cognitive process that feeds into thinking and behaviour more generally. We have previously proposed risk tolerance as a candidate process^13^. In a repeated-measures implementation of the Balloon Analogue Risk Task (BART^17^) that was counterbalanced for order effects, risk tolerance decreased from Monday to Thursday then increased on Friday. This same distinctive pattern was observed in UK polling data for high-stakes political decisions^13^.

One of the difficulties in establishing a causal connection between the weekly cycle and a pattern of behaviour is the unrelenting nature of the cycle itself. From an experimental point of view, it would be informative to remove the weekly cycle and measure any resulting change in the behaviour of interest. For example, if the behavioural pattern were to disappear after the weekly cycle was suspended, that would suggest a causal role for the weekly cycle in maintaining the behaviour.

In practice, of course, we cannot suspend the weekly cycle. Instead, researchers have relied on minor perturbations to the weekly cycle, such as phase offsets caused by long weekends^1^ or differences in cultural conventions^18^.

The COVID-19 pandemic presented a unique opportunity to study a major disruption to the weekly cycle. Although the imposed lockdowns did not strictly suspend the weekly cycle, they loosened its grip on large parts of the population by placing millions of people on furlough and requiring others to stay at home. Many whose routines were disrupted reported losing track of what day it was or complained that all days began to feel the same—a phenomenon known as *Blursday* in the media^19^.

In Study 1, we used this unique circumstance to examine the connection between reported salience of the weekly cycle (perception) and weekly fluctuations in risk tolerance (behaviour). Specifically, we compared risk measures for participants who reported a normal or strong sense of weekday (Normal/Strong SOW) and participants who reported a weak sense of weekday (Weak SOW). We predicted that the Normal/Strong group would show the same weekly risk cycle that we have seen elsewhere, with risk tolerance declining from Monday to Thursday then rebounding on Friday. However, we also predicted that this pattern would be attenuated in the Weak group, resulting in a flatter function for that group specifically. To explore the generality of these effects and their relation to different aspects of risk, we gathered from each participant several standard measures of risk that have been developed for different purposes. Regularities between these different measures should give us more confidence in the overall pattern and its scope.

The first study was conducted in May 2020, four weeks into the first UK lockdown since World War II. At this stage, disruption to weekly routines was considerable and widespread. Working from home had increased to 35.9%^20^ and at its peak 29% of workers were furloughed^21^. In view of this disruption, it seemed likely that those affected would report a weaker sense of the weekday than they had before (Weak SOW), while people who were unaffected would report a sense of the weekday that was just as strong as normal (Normal/Strong SOW). Our main interest was whether a difference in SOW would impact the weekly risk cycle. Based on previous findings, we expected risk scores in the Normal/Strong SOW group to exhibit the following features: (i) systematic change through the week, rather than random fluctuation, (ii) decreasing, rather than increasing, risk tolerance from the start of the week, and (iii) inflection point on Thursday, such that risk tolerance on Friday is higher. Observing this very specific pattern in different risk measures should increase our confidence in the effect. If the weekly risk cycle depends on a clear idea of what day it is, then this pattern should be reduced or eliminated in the Weak SOW group, in a manner that is consistent across risk measures. We had no specific predictions concerning the weekend days but included them throughout for completeness.

The cycles we live by are laden with associations: night is associated as darker than day, winter as colder than summer, Friday as preferable to Monday^1,22^. Yet the origins of these associations are very different. Diurnal and seasonal associations follow the clockwork of the solar system and are written into our biological inheritance^23,24^.

In Study 2, we again examined the weekly risk cycle, this time during the second UK lockdown in November 2020. The design was similar to Study 1, using the same risk measures and the same comparison of Normal/Strong SOW versus Weak SOW groups. The most important difference was that Study 1 followed a period of stability in the weekly cycle (the decades preceding COVID-19 restrictions), during which we would expect normal weekday associations to have been continually reinforced. In contrast, Study 2 followed a period of severe disruption (the months of COVID-19 restrictions), during which we would expect normal weekday associations to be reinforced much less.

As with Study 1, we expected the weekly risk cycle to be absent in the Weak SOW group. Our main interest was in the Normal/Strong SOW group. If weekday associations are sustained via social structure, those associations should dissipate over prolonged disruption to those structures. In that situation, knowing what day it is should make no difference. A strong sense of weekday is meaningless if the weekdays have lost their meaning. It follows that a weekly risk cycle that is based on weekday associations should also dissipate, even in the Normal/Strong SOW group.

If normal social structure is not required to sustain weekday associations, or the weekly risk cycle does not depend on weekday associations, then the weekly risk cycle in the Normal/Strong SOW group should be as strong as it was in Study 1.

## Methods

### Materials and design (Study 1 & 2)

Each participant completed four risk assessments, reported on their sense of weekday, and provided answers to basic demographic questions. Specifically, both studies used four different risk assessments capturing different aspects of risk tolerance^25^ that have been associated with different real-world behaviours: the Domain-Specific Risk Task (DOSPERT) questionnaire^26^; the German socioeconomic panel self-reported question (SOEP^27^); the incentive-compatible multiple lotteries gambling task (BEG^28,29^); and the performance-incentivised Balloon Analogue Risk Task (BART^17^). The BART and BEG are performance-incentivised tasks where participants had random chances of receiving the task pay-out in addition to their base pay. This diversity of risk measurements covers both actual risk-taking behaviour (BART, BEG) and general risk attitude (DOSPERT, SOEP). We believe that this spread of different risk measurements allows us to paint a more complete picture of an individual’s risk attitude.

#### DOSPERT

Risk-taking has been shown to vary by domain^30,31^. The DOSPERT questionnaire asks participants to self-report the likelihood that they would participate in a certain risky activity (Likert scale from 1 to 7), with the activities purposefully spanning different domains: ethics, recreational, health & safety, social, and financial decisions. The DOSPERT subscales have demonstrated real-world validity in these separable domains (e.g.^32^). To arrive at a collective risk score as well as the five domain-specific risk scores, the average across the respective responses is calculated.

#### SOEP

The SOEP, originating from the German Socio-Economic Panel longitudinal study, asks participants to self-report their willingness to take risks using a 0–10 Likert scale^27^. Participants in our study were presented with both a general question, asking directly how prepared the participant was to take risks in general, as well as five specific questions duplicating the general wording, but asking regarding health, financial, career, driving, and leisure and sports risks. For its simplicity, the SOEP is used in many panel cohorts and has shown to be predictive of various behaviours^33,34^.

#### BEG

The BEG is a multiple lotteries task, wherein participants select one gamble between six options^28,29^. Each gamble has two outcomes both with a 50% probability of occurring. Importantly, the expected value of each gamble increases but also presents a larger difference between the two outcomes (ranging from a certain pay-out of £28, to a gamble with a 50/50 chance of paying out £2 or £70). There was also an option presented to opt out and not participate in the gamble at all. The BEG is a common behavioural measure developed to assess risk preferences, and their applications to decision making and risk taking^28,29,35,36^. Across the studies, the participants had a 1 in 100 chance to be paid the outcome of their lottery choice.

#### BART

The BART measures risk taking through a virtual balloon-pumping task^17^. Participants are presented with a series of 20 balloons that they can inflate incrementally through clicking. The value of the balloon increases by a set amount (£0.01) per pump. However, each balloon will pop at a certain volume (based on a probability distribution unknown to the participants), bringing its value to zero. As such, a participant must balance increasing their pay-out from each balloon with the increasing risk of the balloon popping and losing the money for this particular balloon. For each participant, the *adjusted BART score* is calculated, as the average number of pumps for balloons that did not pop. The BART is a task developed in health psychology and shows to be most predictive of health risk behaviours such as smoking (e.g.^37^) or drinking (e.g.^38^). For the purpose of this study, the task was adapted for online use. For scalability, we also used a level of abstraction with respect to the stakes: rather than a direct pay-out of winnings, the participants had a 1 in 20 chance to be paid the winnings from the task. Based on the participants performance it was possible to earn a total bonus of up to GBP 81.80 across the two performance-incentivised tasks. While these tasks are designed to be incentive-compatible, we acknowledge that payment of tasks may not be enough to ensure true incentive compatibility^39–43^.

#### Sense of weekday

In order to determine whether risk fluctuation may depend on subjective experiences of time, we separated participants by their self-reported *sense of weekday* (SOW). Each participant responded to the question “During lockdown, my sense of which day of the week we are on is…?” on a scale of much weaker than usual (1) weaker than usual (2) the same as usual (3) stronger than usual (4) much stronger than usual (5) by means of a manipulation check as to whether their experience of time had or had not shifted.

#### Demographic questions

Participants also reported on their age, gender, and employment.

### Study 1

#### Participants and procedure

864 paid participants were recruited via Prolific Academic (http://www.prolific.co^44^) across 14 days from May 11 to May 24, 2020 (n = 122–128 per weekday; mean age = 32.9 years, age range = 18–77; 67.8% female; see Supplementary Materials Table A for a demographic breakdown by weekday). For their participation, the participants received a fixed payment of GBP 2.00 (Study 1) and GBP 3.00 (Study 2). Moreover, participants had the chance to be paid an additional bonus of up to GBP 81.80 based on two performance-incentivised tasks. Participants provided informed consent in line with the University Research Ethics Committee requirements (ethics approval number 07564) and were compensated in line with Prolific’s wage guidelines.

### Study 2

#### Participants and procedure

829 paid participants were recruited via Prolific Academic across 14 consecutive days during a UK government lockdown between 16 and 29 November 2020 (117–121 participants per day of week; mean age = 32.7 years, age range = 18–75; 71.9% female; see Supplementary Material F for a detailed breakdown of participant demographics). Participants provided informed consent in line with the LSE Research Ethics Committee requirements (ethics approval number 07564) and were compensated in line with Prolific’s wage guidelines.

The procedure, materials and data analysis of Study 2 were identical to Study 1, bar one adjustment.

Similar to Study 1, each participant responded to the question “During this lockdown, my sense of which day of the week we are on is…?” on a scale of much weaker than usual (1) weaker than usual (2) the same as usual (3) stronger than usual (4) much stronger than usual (5). This differs from the question in Study 1 with the addition of the word “this”, to make sure it is clear which lockdown was being referred to.

## Data analysis

Using a linear regression model, the primary dependent variable for our analysis was a composite risk score, calculated in a three-step process. First, scores for each of the above risk measures were calculated by participant, as per the respective standard procedure^17,26–29^. Then, all individual scores across each risk measurement (and each subscale for the DOSPERT and SOEP) were converted into Z-scores. Third, the Z-scores were averaged across the four risk measures for each participant to obtain a single composite risk score. The choice of this methodology for computing the composite score builds upon the equal weight, both computationally and theoretically, of each risk measurement.

Subsequent analyses divided participants into two groups by sense of weekday (SOW). Therefore, each analysis was conducted once for those with a Normal/Strong SOW and once for those with a Weak SOW.

As independent variables we used the day of the week (Monday, Tuesday, Wednesday, Thursday, Friday, Saturday, Sunday). We categorise the sense of the weekday by splitting it into two groups: weak (Much weaker (1) or weak (2)) and strong (normal (3), strong (4) or much stronger (5)).

We additionally control for gender and age effects in the model which have been shown to be important predictors of risk tolerance. In particular, men have been found to be more risk tolerant than women^45–48^ and age to be inversely related to risk tolerance^49–51^. In case of any imbalances in the sample, incidental effects of age and gender may appear and can be accounted for.

To check for consistency across the different risk measures, we repeat this analysis for each risk measure independently and report these findings in the Supplementary Materials.

## Results

### Study 1

Firstly, we note that there were no demographic differences between participants who self-reported a Weak or Normal/Strong SOW across the seven weekdays (see Supplementary Materials Table A). We note that we did not use weighting in the analysis to account for demographic variations.

**Figure 1:**
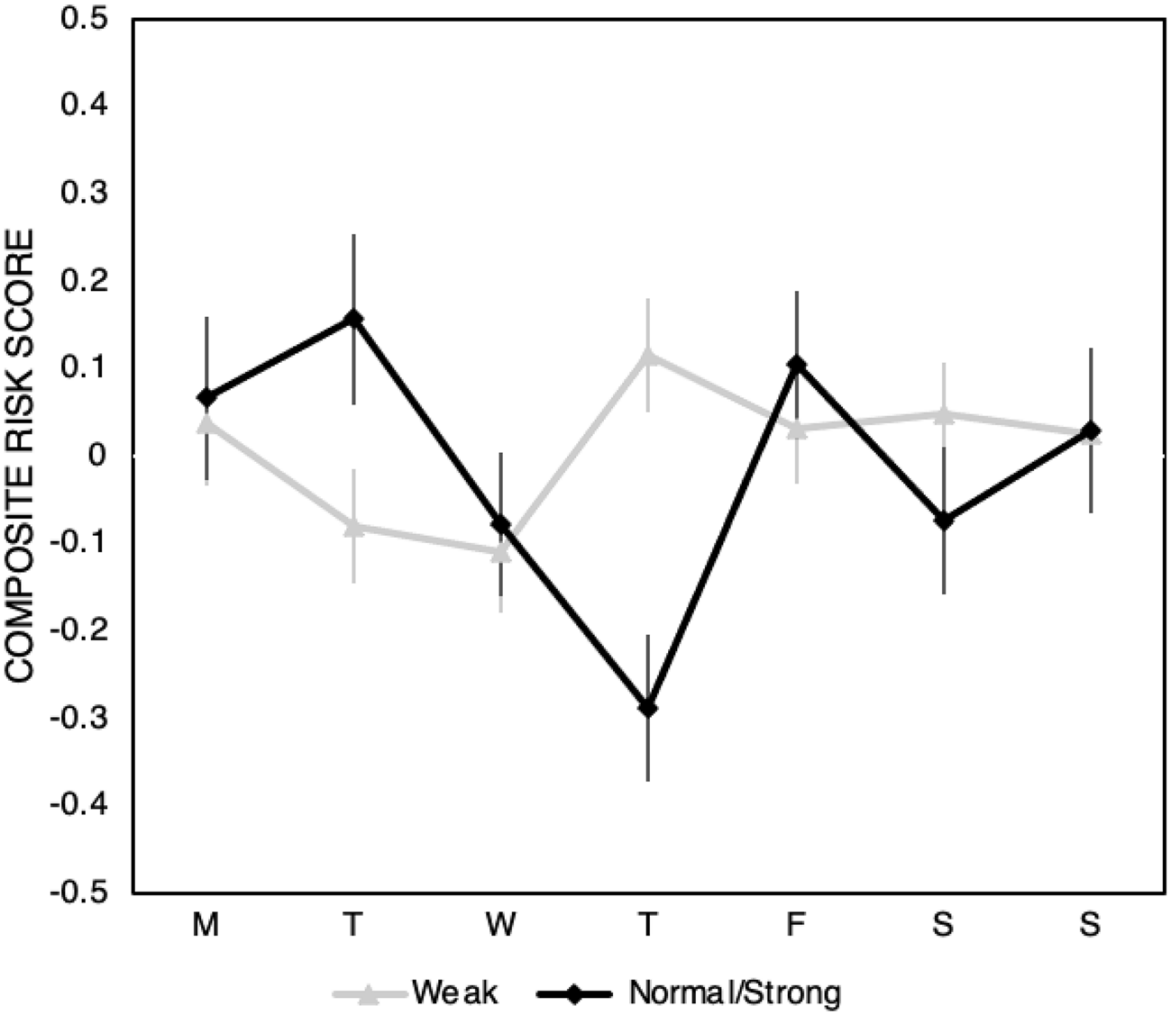
Composite risk score separated by participants with a Normal/Strong vs Weak sense of weekday plotted across the days of the week during the first lockdown. Error bars represent +/− SE.

Figure 1 shows the composite risk score by weekday separately for participants who report a Normal/Strong SOW and those who report a Weak SOW. Table 1 shows the associated values.

**Table 1:** Composite risk score for Normal/Strong and Weak SOW across days of the week (mean, standard error, 95% CI).

Sense	Day of the week	Mean	Standard error	95% CI
Strong/Normal	Monday	0.066	0.093	[− 0.116, 0.248]
	Tuesday	0.157	0.098	[− 0.034, 0.349]
	Wednesday	− 0.077	0.082	[− 0.239, 0.084]
	Thursday	− 0.288	0.084	[− 0.454, − 0.123]
	Friday	0.104	0.084	[− 0.061, 0.269]
	Saturday	0.073	0.084	[− 0.238, 0.092]
	Sunday	0.030	0.095	[− 0.156, 0.216]
Weak	Monday	0.037	0.071	[− 0.102, 0.176]
	Tuesday	− 0.079	0.066	[− 0.208, 0.049]
	Wednesday	− 0.11	0.068	[− 0.244, 0.023]
	Thursday	0.116	0.065	[− 0.011, 0.243]
	Friday	0.032	0.063	[− 0.092, 0.156]
	Saturday	0.048	0.059	[− 0.067, 0.163]
	Sunday	0.260	0.063	[− 0.097, 0.149]

A linear regression for those with a Normal/Strong SOW of weekday on composite risk score (adjusted R^2^ = 0.037) reveals an effect of weekday (Effect size η^2^ = 0.058, 95% CI [0.003, 0.103]) driven by Thursday–Monday (Estimate = − 0.355, SE = 0.127, 95% CI [− 0.604, − 0.105] t = − 2.802, p = 0.005). See Supplementary Materials Tables B.1. for full reporting and Supplementary Material Tables B.2. for post-hoc comparisons. Additionally, see and Supplementary Material Tables B.3.–B.4. for full reporting and post-hoc comparisons of a model including additional demographic controls (age, gender).

A linear regression for those with a Weak SOW of weekday on composite risk score (adjusted R^2^ = 0.004) reveals no main effect of weekday. See Supplementary Materials Table B.5. for full reporting and Supplementary Material Table B.6. for post-hoc comparisons. Additionally, see and Supplementary Material Tables B.7.–B.8. for full reporting and post-hoc comparisons of a model including additional demographic controls (age, gender).

The same analysis was performed for each risk measure separately, again dividing by sense of weekday into two groups (Normal/Strong SOW, Weak SOW). Analyses of both weekday only and of weekday, age, gender are both reported. See Supplementary Materials C for descriptives of each measure and Supplementary Materials Fig. D and Tables D.1. to B.32. for details of each independent analysis and Supplementary Materials Fig. E and Tables E.1. to E.8. for calculation of the composite risk score without the inclusion of BART. For the Normal/Strong SOW specifically, the Mon–Thursday dip was significant across both composite score variations (calculated with and without BART), as well as the SOEP and DOSPERT, but not the BEG or the BART.

### Study 2

**Figure 2:**
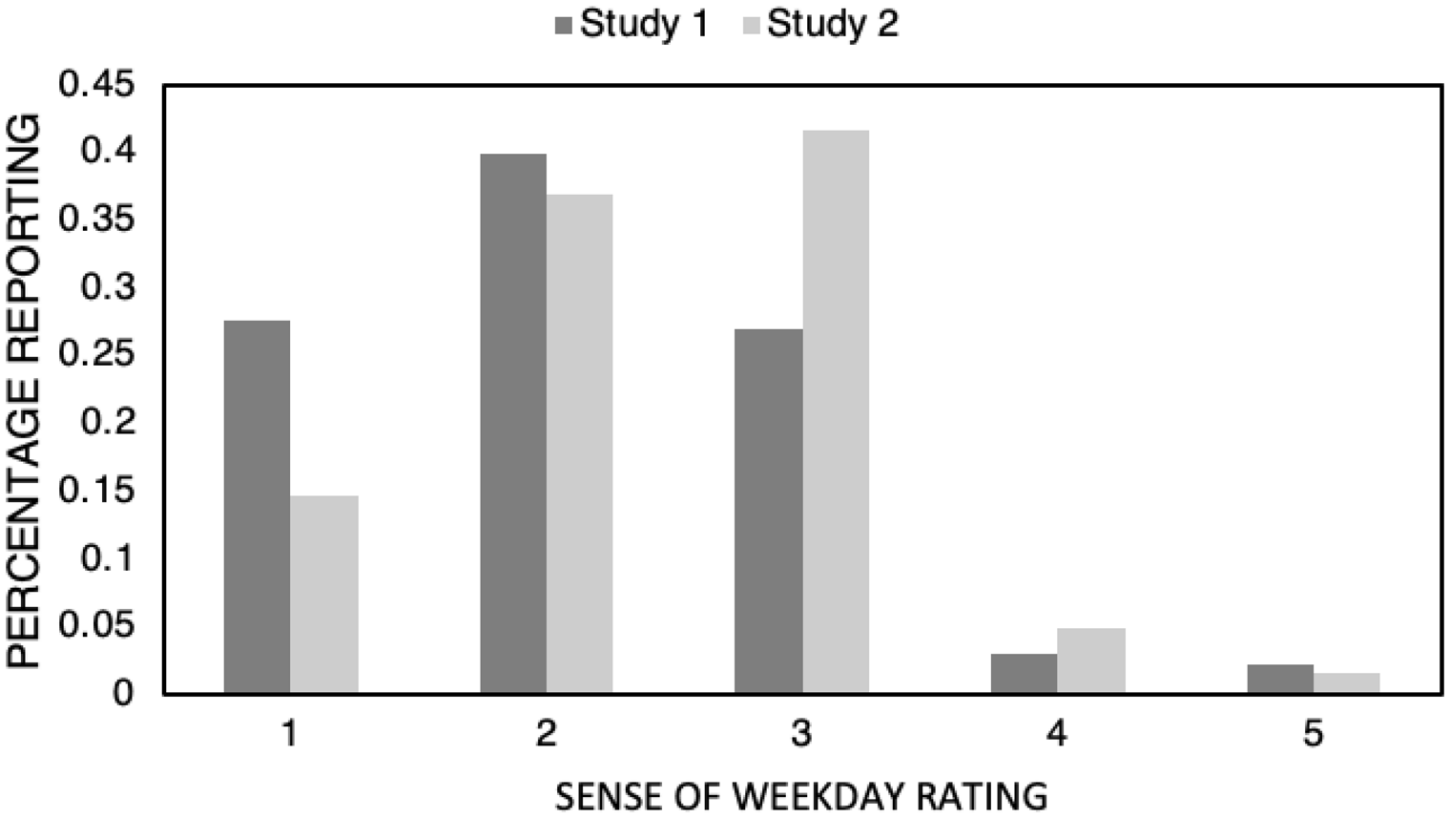
The distribution of participant scores for Study 1 and Study 2 for the question “How strong is your sense of weekday?” on a scale of 1 (much weaker) to 5 (much stronger).

Figure 2 shows the distribution of SOW for the two studies. To check whether the experience of the days of the week had shifted between the first and second lockdown, we compared Sense of Weekday (SOW) ratings obtained in Study 2 (lockdown 2) with those obtained in Study 1 (lockdown 1). An independent samples t-test (t(1690) = − 6.25, p < 0.001; Cohen’s d = 0.332) indicates that people reported a stronger sense of weekday on average in the second lockdown (M = 2.420, SE = 0.0300, Mode = 3) than in the first (M = 2.123, SE = 0.032, Mode = 2).

**Figure 3:**
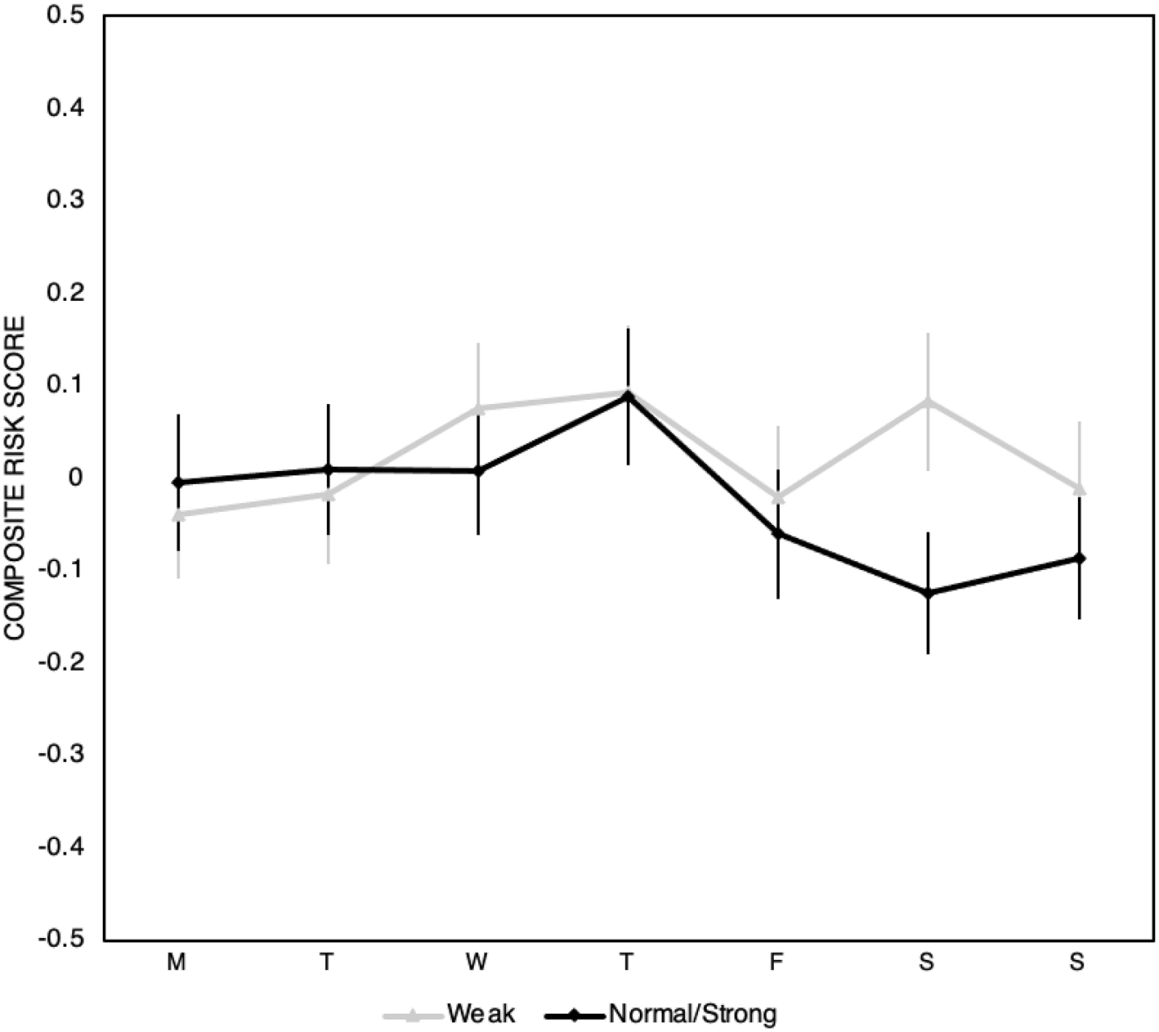
Composite risk score across weekdays separated by weak and strong sense of weekday. Error bars represent +/− SE.

Figure 3 shows the composite risk score separated by weekday for those who report a strong and those who report a weak SOW during the second lockdown. Table 2 shows the associated values.

**Table 2:** Composite risk score for Normal/Strong and Weak SOW across days of the week (mean, standard error, 95% CI).

Sense	Day of the week	Mean	Standard error	95% CI
Normal/Strong	Monday	− 0.004	0.074	[− 0.149, 0.140]
	Tuesday	0.0103	0.071	[− 0.130, 0.149]
	Wednesday	0.007	0.069	[− 0.127, 0.142]
	Thursday	0.088	0.074	[− 0.058, 0.234]
	Friday	− 0.061	0.069	[− 0.197, 0.076]
	Saturday	− 0.125	0.066	[− 0.255, 0.006]
	Sunday	− 0.087	0.067	[− 0.217, 0.044]
Weak	Monday	− 0.039	0.068	[− 0.173, 0.095]
	Tuesday	− 0.018	0.073	[− 0.161, 0.125]
	Wednesday	0.075	0.070	[− 0.061, 0.212]
	Thursday	0.092	0.072	[− 0.048, 0.233]
	Friday	− 0.021	0.075	[− 0.168, 0.127]
	Saturday	0.083	0.073	[− 0.060, 0.225]
	Sunday	− 0.011	0.071	[− 0.150, 0.128]

A linear regression for those with a Normal/Strong SOW of weekday on composite risk score (adjusted R^2^ = 6.884e-4) reveals no main effect of weekday. See Supplementary Materials Table G.1. for full reporting and Supplementary Material Table G.2. for post-hoc comparisons. Additionally, see and Supplementary Material Tables G.3.–G.4. for full reporting and post-hoc comparisons of a model including additional demographic controls (age, gender).

A linear regression for those with a Weak SOW of weekday on composite risk score (adjusted R^2^ = − 0.005) reveals no main effect of weekday. See Supplementary Materials Table G.5. for full reporting and Supplementary Material Table G.6. for post-hoc comparisons. Additionally, see and Supplementary Material Tables G.7.–G.8. for full reporting and post-hoc comparisons of a model including additional demographic controls (age, gender).

The analysis was repeated for each risk measure separately, again through separate analyses for those with Normal/Strong and Weak SOW (see Supplementary Material Fig. H for descriptives, and Fig. I and Tables I.1. to I.16 for details of analysis). As in Study 1, see Supplementary Material Fig. J and Tables J.1. to J.4. for comparison of the composite risk score as calculated with and without the inclusion of BART. Across all additional analyses, both for Normal/Strong and Weak SOW groups, there was no main effect of weekday.

## Discussion

### Study 1

This study makes a number of contributions. First and foremost, among those who reported a strong sense of weekday, we found a similar pattern of weekly fluctuations in risk tolerance to^13^. As with the original findings^13^, risk tolerance began high in the beginning of the week, reached its lowest point on Thursday, and rebounded on Friday. The similarity of this pattern across studies is especially interesting given the differences between studies. The original study was conducted with a student sample in a laboratory setting, using a repeated measures design. The current study was conducted with a general population sample in an online setting, using a between-subjects design. Conservation of the basic pattern across these design changes suggests a high degree of generalisability.

Interestingly, the only measures that did not show the pattern is the BART and the BEG, the two tasks measuring actual risk taking (compared to self-reported, such as the DOSPERT and SOEP). At first sight, this may seem surprising, as the BART is the measure with which the pattern was originally observed. We explore further peculiarities of BART in the "General discussion" section that may have contributed to this finding. However, it is interesting to note that the effect of weekday fell cleanly along the split between tasks measuring actual risk taking and risk attitudes. We hypothesise that this discrepancy between the DOSPERT/SOEP and BART/BEG may be due in part to the relationships between the different types of measures and risk-taking behaviour: in a direct comparison, risk-taking questionnaires (in the present study, comparable to the SOEP and DOSPERT) have been shown to have a higher test–retest reliability and correlation with actual risk-taking behaviour than lottery-choice tasks (such as the BEG)^52^. The choice of risk task in experimental work has long been a point of interest^53^, and we tentatively suggest that this difference in type of test may describe the present study’s findings.

In another extension to previous work, we also collected data on weekend days. For the Strong SOW group, risk tolerance on Saturday and Sunday was similar to that on Monday, Tuesday and Friday, suggesting that the observed pattern is better characterised as a midweek dip than as peaks that bookend the working week. Given the human origins of the weekly cycle, we are inclined to attribute weekly fluctuations in risk tolerance to human causes, such as semantic or emotional associations with the days of the week. In the next study, we had the opportunity to examine the impact of COVID-19 restrictions over the longer term, when such associations may have atrophied.

### Study 2

We found no evidence in Study 2 for the weekly cycle in risk tolerance seen in Study 1. Critically, the cycle was abolished even among people who retained a strong sense of weekday. We suggest that 30 weeks without normal reinforcement of weekday associations was enough to decouple mere knowledge of the current day from its usual ramifications.

### General discussion

In the current studies, we used the unique circumstance of the COVID-19 lockdown to examine connections between reported salience of the weekly cycle (perception) and weekly fluctuations in risk tolerance (behaviour). Our results corroborate the findings of previous studies: risk tolerance decreased from Monday to Thursday and increased on Friday. However, the current studies demonstrate this cycle using different measures of risk. They also identify conditions under which the weekly risk cycle emerges.

We begin by considering similarities between Study 1 and Study 2. In both studies, a portion of respondents reported that their sense of weekday was at least as strong as it had been before lockdown. Apparently, their sense of weekday was not perturbed by the onset (Study 1) or continuation (Study 2) of lockdown restrictions. There are at least two possible reasons for this resilience. The first appeals to situational factors^54^. For example, those reporting a strong sense of weekday might have continued their normal work pattern. The second appeals to dispositional factors. For example, the days of the week could be more salient to some people than to others. The latter suggests a more trait-like attribute, perhaps analogous to sense of direction. This analogy between sense of weekday and sense of direction seems potentially fruitful. A few studies have examined psychometric properties of sense of direction and identified clear personality correlates (e.g.^55^). Some aspects of previous findings suggest that sense of weekday could be amenable to similar analyses. For example, studies requiring participants to name the current day have shown broad distribution in performance^1,56^. As of yet however, no studies have taken an individual differences approach to the salience of the weekly cycle. One possible exception concerns studies of calendrical savants, who can rapidly report the weekday corresponding to a given date (e.g.^57^). Such individuals demonstrate that it is possible to be highly attuned to the days of the week. However, it is not clear whether this ability represents one extreme on a continuum of sensitivity or a qualitatively distinct skill.

We now turn to differences between Study 1 and Study 2. Even among participants who reported a strong sense of weekday, the weekly risk cycle was very different earlier during COVID-19 restrictions (Study 1) compared with later during the restrictions (Study 2). This finding shows that the weekly risk cycle is not reducible to sense of weekday and is dissociable from it. The absence of a weekly risk cycle in Weak SOW participants (Studies 1 & 2) suggests that a Strong SOW is *necessary* for the weekly risk cycle to occur. The absence of a weekly risk cycle in Strong SOW participants (Study 2 only) suggests that Strong SOW is not *sufficient*. Some other factor, present in Study 1 but not in Study 2, is also required for the weekly risk cycle to emerge. It is inevitable that the two studies differed in many ways that cannot be equated. For example, Study 1 was conducted in spring, whereas Study 2 was conducted in autumn; the participant samples contained different people. In view of such mismatches, we should be cautious in attributing divergent outcomes to any single cause. At the same time, part of the motivation behind this project was the temporal disorientation that people reported during COVID-19 restrictions, specifically concerning the days of the week^19,58,59^. Duration of disruption becomes key here. While participants in Study 1 had experienced only 4–5 weeks of disruption, participants in Study 2 had experienced 31–32 weeks of disruption. How might this factor be important? Our working hypothesis is that stereotypical weekday associations underpinning the weekly risk cycle require reinforcement. Normally, this reinforcement is supplied by the social environment—directly, as we adhere to weekly routines ourselves, and indirectly as we interact with others as they adhere to weekly routines. When this reinforcement is withdrawn (as during COVID-19 restrictions), weekday associations begin to decay suggesting a shift in what is understood as a ‘normal’ SOW, with association strength proportional to elapsed time. This is further supported by the larger proportion of individuals reporting a Normal/Strong SOW in Study 2, as we hypothesise the understanding of a ‘normal’ SOW shifted over the course of COVID-19 restrictions.

**Figure 4:**
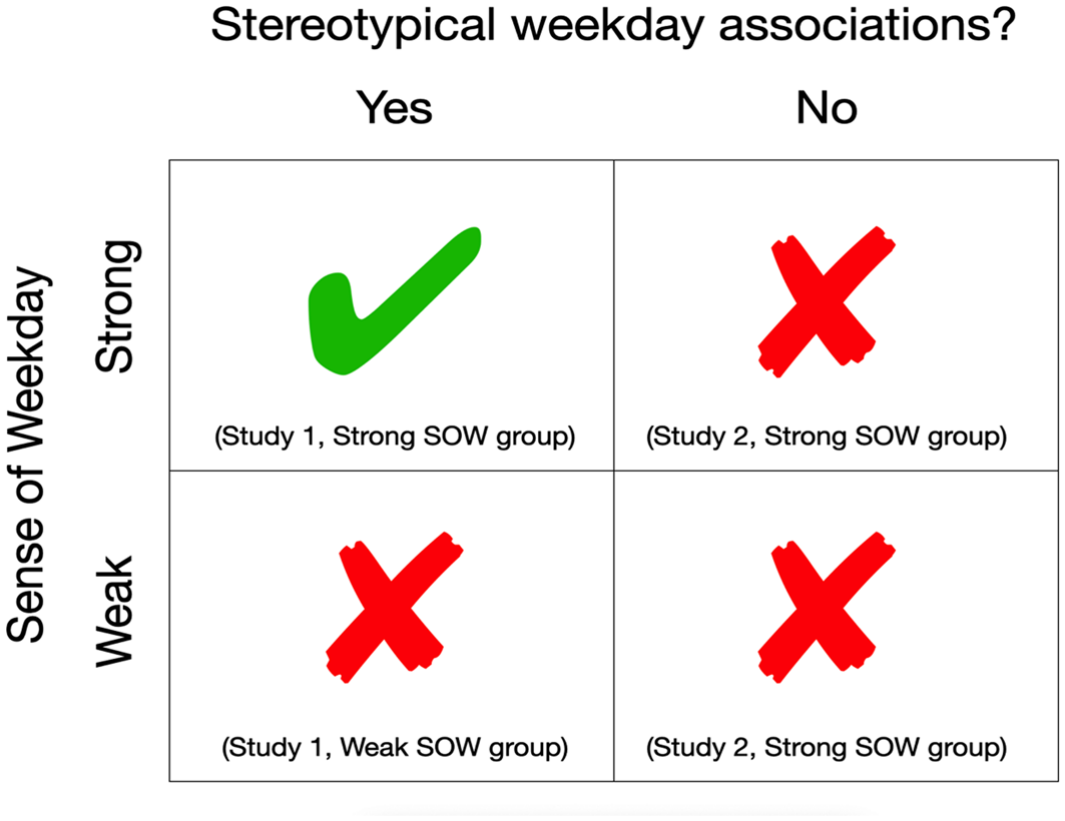
Factors affecting the weekly risk cycle. Rows refer to sense of weekday, which may be strong or weak. Columns refer to stereotypical weekday associations, which may or may not be maintained. Quadrants show the mapping of the current studies onto these factors. The weekly risk cycle occurs only when sense of weekday is strong and weekday associations are maintained (Top Left).

The upshot is that there are at least two ways in which the weekly risk cycle can fail. If sense of weekday is weak, then weekday will have little effect because the current day is not salient. This applies irrespective of weekday associations. If stereotypical weekday associations atrophy, then weekday will have little effect because the current day is not meaningful. This applies irrespective of sense of weekday. Figure 4 summarises our interpretation.

One further observation seems worth noting. In Study 2, there was no statistically significant effect of weekday in the Normal/Strong SOW group. In other words, risk scores were not statistically different from one day to the next. However, a separate question we can ask is: On which day of the week were risk scores most extreme? For the SOEP, the DOSPERT, and the BEG alike (but not the BART), the answer is Thursday. This observation is curious for two reasons. First, it seems improbable that the most extreme day should again be Thursday rather than some other day of the week. Second, for all three measures the deviation in Study 2 was in the opposite direction to the deviation in Study 1 (with Thursday being the most risk tolerant day rather than the most risk averse day). Again, the difference in Study 2 was not statistically significant.

A note on the different risk measures in this study. The first laboratory demonstration of a weekly risk cycle reported fluctuations in BART scores^13^. Our intention here was to use the same measure to examine the weekly risk cycle during lockdown. We also administered the DOSPERT, the SOEP, and the BEG to test whether the same weekly risk cycle was evident in other measures. As it turned out, the DOSPERT and the SOEP showed the weekly risk cycle. However, the BART and the BEG did not. How did we arrive at this puzzling outcome? The first noteworthy difference is that, by design, the DOSPERT and SOEP measure *risk attitudes*, while the BEG and the BART measure actual *risk taking* through use of tasks (gambles and balloon inflation, respectively).

Further, comparisons of BART designs provide some useful clues. Ferrey and Mishra, Xu et al.^60,61^ demonstrate that the sensitivity of the BART depends on reward structure. We made several changes to reward structure to accommodate online testing. For example, Sanders and Jenkins^13^ involved a laboratory setting, larger rewards, and a more concrete representation of the stakes. In contrast, the current version involved an online setting, smaller rewards, and a more abstract representation of the stakes. We introduced these changes in an effort to make data collection more efficient. However, we believe that they may have blunted the sensitivity of the test. Separate analyses, unrelated to weekday effects, support this interpretation. For example, scores from the current implementation of the BART did not correlate with scores on other risk measures^47^. Nor did they detect well-established sex differences in risk taking^62^. Given these reservations, we recognise that there is a case for setting aside the current BART data: incorporating an insensitive measure into the combined risk score can only dilute the pattern of interest. We choose to include them here to avoid selective reporting, to reflect our uncertainty in the source of the discrepancy, and to underscore the insightfulness of^61^ analysis. For the interested reader, we present combined risk scores that exclude the BART in Supplementary Materials. These alternative scores show the weekly risk cycle more emphatically (Study 1, Normal/Strong SOW), but otherwise support the same conclusions.

Despite the early stages of research in this area, there are already some clear predictions emerging from the work presented here. First, sense of weekday should reveal substantial individual differences, such that some people are more attuned to the weekly cycle than others. By analogy to sense of direction, we expect sense of weekday to be a trait-like attribute that generalises across different measures and is stable over time. Second, weekday associations should be malleable. This proposal could be tested by comparing weekday associations of people with unusual work patterns, for example, people who work weekends and take days off midweek (i.e., cross-sectional comparison). We expect that weekday associations in such groups will differ from stereotypical associations in systematic ways. Third, loss of weekday associations (or acquisition of new ones) should occur somewhere in a 4- to 30-week time window (the number of weeks between the two lockdown periods). A more precise time course could be established by studying transitions into or out of unusual weekly routines (i.e., longitudinal comparison as people retire, leave or enter a period of incarceration, start or leave work on an oil rig or cruise ship). Studying such transitions would also allow us to test directly for repulsion aftereffects when an entrained pattern ceases, namely whether suspension of an entrained weekly cycle, with its midweek dip in risk tolerance, might also induce a repulsion aftereffect, such that the midweek dip is temporarily reversed (i.e., a midweek boost in risk tolerance). Lastly, we believe there is scope to explore different stereotyped behavioural patterns associated with the day of the week beyond risk attitudes. Further explorations of a range of cognitive and individual traits fluctuating over days of the week could further add to this body of research.

For now, we show that the weekly cycle in risk tolerance generalises across several standard measures of risk. We identify two enabling conditions for the observed cycle: strong sense of weekday and stereotypical weekday associations. When both conditions were met, the weekly risk cycle was strong and consistent. Withdrawing either condition abolished the effect.

### Supplementary Information

Supplementary Information.

## Data Availability

The datasets generated and/or analysed during the current study are available in the OSF repository https://osf.io/h8rq9.
