# Supplementary material for: Weakened weekdays: lockdown disrupts the weekly cycle of risk tolerance
Source: Sci Rep. 2023 Nov 30;13:21147. doi: 10.1038/s41598-023-48395-9 (PMC10689774; doi:10.1038/s41598-023-48395-9)
Supplement: Supplementary file 1 — Supplementary Information. [file 41598_2023_48395_MOESM1_ESM.docx]

**Study 1:**

##

***Supplementary Table A:** Demographic breakdown by weekday and Sense of Week (SOW) in Study 1. Chi-square test was used to determine any deviations in observed frequencies of males in strong [χ2(6, N = 172) = 4.28, p = 0.64)] and weak [χ2(6, N = 106) = 6.99, p = 0.32)] groups. A t-test was used to determine whether there were significant variations in ages between the strong/normal and weak groups and was found to be non-significant [t(12) = -0.90, p= 0.39]. There were significantly more males in the Normal/Strong SOW group than in the Weak SOW group significant [t(517.9) = -2.446, p = 0.015].

| **Sense of weekday** | **Day of the Week** | **N** | **% Male** | **Average Age (***σ*_M_**)** |
| --- | --- | --- | --- | --- |
| **Strong, Normal** | *Monday* | 41 | 41.46 | 32.93 (1.53) |
|  | *Tuesday* | 43 | 30.23 | 33.58 (1.77) |
|  | *Wednesday* | 47 | 32.61 | 35.66 (1.75) |
|  | *Thursday* | 37 | 24.32 | 34.43 (2.15) |
|  | *Friday* | 36 | 52.78 | 32.08 (1.78) |
|  | *Saturday* | 34 | 44.11 | 31.76 (1.60) |
|  | *Sunday* | 39 | 43.59 | 32.74 (1.79) |
| **Weak** | *Monday* | 81 | 34.57 | 34.04 (1.30) |
|  | *Tuesday* | 80 | 25.93 | 33.86 (1.46) |
|  | *Wednesday* | 76 | 27.63 | 32.55 (1.28) |
|  | *Thursday* | 84 | 19.27 | 32.59 (1.42) |
|  | *Friday* | 86 | 34.88 | 31.21 (1.37) |
|  | *Saturday* | 94 | 31.91 | 33.21 (1.38) |
|  | *Sunday* | 83 | 46.66 | 31.60 (11.46) |

***Supplementary Table B.1.:**  Results of generalized linear model for Z-scored composite risk score for those with a Normal/Strong SOW of weekday only.

|  |  |  | **95% Confidence Interval** | | |  |  |  |
| --- | --- | --- | --- | --- | --- | --- | --- | --- |
| **Effect** | **Estimate** | **SE** | **Lower** | **Upper** | **β** | **df** | **t** | **p** |
| (Intercept) | 0.009 | 0.034 | -0.058 | 0.076 | 0 | 266 | 0.264 | 0.792 |
| Tue - Mon | 0.092 | 0.123 | -0.151 | 0.335 | 0.162 | 266 | 0.746 | 0.456 |
| Wed - Mon | -0.143 | 0.12 | -0.379 | 0.093 | -0.251 | 266 | -1.192 | 0.234 |
| Thu – Mon** | -0.355 | 0.127 | -0.604 | -0.105 | -0.623 | 266 | -2.802 | 0.005 |
| Fri - Mon | 0.036 | 0.128 | -0.215 | 0.287 | 0.063 | 266 | 0.283 | 0.777 |
| Sat - Mon | -0.141 | 0.131 | -0.398 | 0.116 | -0.248 | 266 | -1.082 | 0.28 |
| Sun - Mon | -0.037 | 0.125 | -0.283 | 0.209 | -0.065 | 266 | -0.295 | 0.768 |

*[* p < 0.05; ** p < 0.01, *** p < 0.001]*

***Supplementary Materials Table B.2.:**  Results of post-hoc comparisons for Z-scored composite risk score for those with a Normal/Strong SOW of weekday only.

| **Weekday** |  |  | **Difference** | **SE** | **t** | **df** | **p** | **p_bonferroni_** |
| --- | --- | --- | --- | --- | --- | --- | --- | --- |
| Fri | - | Sat | 0.177 | 0.135 | 1.318 | 266 | 0.189 | 1 |
| Fri | - | Sun | 0.073 | 0.129 | 0.565 | 266 | 0.572 | 1 |
| Mon | - | Fri | -0.036 | 0.128 | -0.283 | 266 | 0.777 | 1 |
| Mon | - | Sat | 0.141 | 0.131 | 1.082 | 266 | 0.28 | 1 |
| Mon | - | Sun | 0.037 | 0.125 | 0.295 | 266 | 0.768 | 1 |
| Mon | - | Wed | 0.143 | 0.12 | 1.192 | 266 | 0.234 | 1 |
| Mon | - | Thu | 0.355 | 0.127 | 2.802 | 266 | 0.005 | 0.115 |
| Mon | - | Tue | -0.092 | 0.123 | -0.746 | 266 | 0.456 | 1 |
| Sat | - | Sun | -0.104 | 0.132 | -0.791 | 266 | 0.43 | 1 |
| Wed | - | Fri | -0.179 | 0.124 | -1.441 | 266 | 0.151 | 1 |
| Wed | - | Sat | -0.002 | 0.127 | -0.013 | 266 | 0.99 | 1 |
| Wed | - | Sun | -0.106 | 0.122 | -0.873 | 266 | 0.384 | 1 |
| Wed | - | Thu | 0.212 | 0.123 | 1.718 | 266 | 0.087 | 1 |
| Thu | - | Fri | -0.391 | 0.131 | -2.99 | 266 | 0.003 | 0.064 |
| Thu | - | Sat | -0.214 | 0.134 | -1.597 | 266 | 0.112 | 1 |
| Thu | - | Sun | -0.318 | 0.128 | -2.481 | 266 | 0.014 | 0.288 |
| Tue | - | Fri | 0.056 | 0.128 | 0.439 | 266 | 0.661 | 1 |
| Tue | - | Sat | 0.233 | 0.131 | 1.787 | 266 | 0.075 | 1 |
| Tue | - | Sun | 0.129 | 0.125 | 1.032 | 266 | 0.303 | 1 |
| Tue | - | Wed | 0.235 | 0.12 | 1.959 | 266 | 0.051 | 1 |
| Tue | - | Thu* | 0.447 | 0.127 | 3.529 | 266 | < .001 | 0.01 |

*[* p < 0.05; ** p < 0.01, *** p < 0.001]*

***Supplementary Table B.3.:**  Results of generalized linear model for Z-scored composite risk score for those with a Normal/Strong SOW of weekday, age, and gender.

|  |  |  | **95% Confidence Interval** | |  |  |  |  |
| --- | --- | --- | --- | --- | --- | --- | --- | --- |
| **Effect** | **Estimate** | **SE** | **Lower** | **Upper** | **β** | **df** | **t** | **p** |
| (Intercept) | 0.034 | 0.034 | -0.033 | 0.101 | 0.000 | 264 | 0.994 | 0.321 |
| Male – Female** | 0.223 | 0.069 | 0.086 | 0.359 | 0.391 | 264 | 3.215 | 0.001 |
| Age* | -0.008 | 0.003 | -0.014 | -0.002 | -0.151 | 264 | -2.575 | 0.011 |
| Tue - Mon | 0.121 | 0.121 | -0.117 | 0.358 | 0.212 | 264 | 1.000 | 0.318 |
| Wed -Mon | -0.102 | 0.117 | -0.333 | 0.129 | -0.179 | 264 | -0.867 | 0.387 |
| Thu – Mon* | -0.305 | 0.124 | -0.550 | -0.060 | -0.536 | 264 | -2.456 | 0.015 |
| Fri - Mon | -0.003 | 0.125 | -0.249 | 0.242 | -0.006 | 264 | -0.026 | 0.979 |
| Sat - Mon | -0.154 | 0.127 | -0.405 | 0.097 | -0.271 | 264 | -1.211 | 0.227 |
| Sun - Mon | -0.043 | 0.122 | -0.283 | 0.197 | -0.076 | 264 | -0.353 | 0.724 |

*[* p < 0.05; ** p < 0.01, *** p < 0.001]*

***Supplementary Materials Table B.4.:** Results of post-hoc comparisons for Z-scored composite risk score for those with a Normal/Strong SOW of weekday, age, and gender.

| **Weekday** |  |  | **Difference** | **SE** | **t** | **df** | **p** | **p_bonferroni_** |
| --- | --- | --- | --- | --- | --- | --- | --- | --- |
| Fri | - | Sat | 0.151 | 0.132 | 1.149 | 264 | 0.252 | 1 |
| Fri | - | Sun | 0.040 | 0.126 | 0.315 | 264 | 0.753 | 1 |
| Mon | - | Fri | 0.003 | 0.125 | 0.026 | 264 | 0.979 | 1 |
| Mon | - | Sat | 0.154 | 0.128 | 1.211 | 264 | 0.227 | 1 |
| Mon | - | Sun | 0.043 | 0.122 | 0.353 | 264 | 0.724 | 1 |
| Mon | - | Wed | 0.102 | 0.118 | 0.867 | 264 | 0.387 | 1 |
| Mon | - | Thu | 0.305 | 0.124 | 2.456 | 264 | 0.015 | 0.309 |
| Mon | - | Tue | -0.121 | 0.121 | -1.000 | 264 | 0.318 | 1 |
| Sat | - | Sun | -0.111 | 0.129 | -0.864 | 264 | 0.389 | 1 |
| Wed | - | Fri | -0.099 | 0.123 | -0.802 | 264 | 0.423 | 1 |
| Wed | - | Sat | 0.052 | 0.125 | 0.419 | 264 | 0.675 | 1 |
| Wed | - | Sun | -0.059 | 0.119 | -0.494 | 264 | 0.622 | 1 |
| Wed | - | Thu | 0.203 | 0.121 | 1.686 | 264 | 0.093 | 1 |
| Thu | - | Fri | -0.302 | 0.130 | -2.329 | 264 | 0.021 | 0.433 |
| Thu | - | Sat | -0.151 | 0.131 | -1.147 | 264 | 0.252 | 1 |
| Thu | - | Sun | -0.262 | 0.126 | -2.082 | 264 | 0.038 | 0.805 |
| Tue | - | Fri | 0.124 | 0.126 | 0.986 | 264 | 0.325 | 1 |
| Tue | - | Sat | 0.275 | 0.128 | 2.151 | 264 | 0.032 | 0.681 |
| Tue | - | Sun | 0.164 | 0.122 | 1.339 | 264 | 0.182 | 1 |
| Tue | - | Wed | 0.222 | 0.117 | 1.899 | 264 | 0.059 | 1 |
| Tue | - | Thu* | 0.426 | 0.124 | 3.442 | 264 | < .001 | 0.014 |

*[* p < 0.05; ** p < 0.01, *** p < 0.001]*

***Supplementary Table B.5.:**  Results of generalized linear model for Z-scored composite risk score for those with a Weak SOW of weekday only.

|  | **Estimate** | **SE** | **Lower** | **Upper** | **β** | **df** | **t** | **p** |
| --- | --- | --- | --- | --- | --- | --- | --- | --- |
| (Intercept) | -7.397e−4 | 0.024 | -0.049 | 0.047 | 0 | 574 | -0.03 | 0.976 |
| Tue - Mon | -0.116 | 0.093 | -0.299 | 0.067 | -0.197 | 574 | -1.248 | 0.212 |
| Wed - Mon | -0.148 | 0.094 | -0.333 | 0.037 | -0.251 | 574 | -1.567 | 0.118 |
| Thu - Mon | 0.079 | 0.092 | -0.102 | 0.26 | 0.133 | 574 | 0.856 | 0.392 |
| Fri - Mon | -0.006 | 0.091 | -0.186 | 0.174 | -0.01 | 574 | -0.064 | 0.949 |
| Sat - Mon | 0.011 | 0.09 | -0.165 | 0.187 | 0.019 | 574 | 0.123 | 0.902 |
| Sun - Mon | -0.012 | 0.093 | -0.193 | 0.17 | -0.02 | 574 | -0.126 | 0.9 |

*[* p < 0.05; ** p < 0.01, *** p < 0.001]*

***Supplementary Materials Table B.6.:** Results of post-hoc comparisons for Z-scored composite risk score for those with a Weak SOW of weekday only.

| **Weekday** |  | **Weekday** | **Difference** | **SE** | **t** | **df** | **p** | **p_bonferroni_** |
| --- | --- | --- | --- | --- | --- | --- | --- | --- |
| Fri | - | Sat | -0.017 | 0.088 | -0.192 | 574 | 0.848 | 1 |
| Fri | - | Sun | 0.006 | 0.091 | 0.064 | 574 | 0.949 | 1 |
| Mon | - | Fri | 0.006 | 0.091 | 0.064 | 574 | 0.949 | 1 |
| Mon | - | Sat | -0.011 | 0.09 | -0.123 | 574 | 0.902 | 1 |
| Mon | - | Sun | 0.012 | 0.093 | 0.126 | 574 | 0.9 | 1 |
| Mon | - | Wed | 0.148 | 0.094 | 1.567 | 574 | 0.118 | 1 |
| Mon | - | Thu | -0.079 | 0.092 | -0.856 | 574 | 0.392 | 1 |
| Mon | - | Tue | 0.116 | 0.093 | 1.248 | 574 | 0.212 | 1 |
| Sat | - | Sun | 0.023 | 0.089 | 0.254 | 574 | 0.8 | 1 |
| Wed | - | Fri | -0.142 | 0.093 | -1.532 | 574 | 0.126 | 1 |
| Wed | - | Sat | -0.159 | 0.091 | -1.745 | 574 | 0.082 | 1 |
| Wed | - | Sun | -0.136 | 0.094 | -1.453 | 574 | 0.147 | 1 |
| Wed | - | Thu | -0.227 | 0.093 | -2.43 | 574 | 0.015 | 0.323 |
| Thu | - | Fri | 0.085 | 0.09 | 0.936 | 574 | 0.35 | 1 |
| Thu | - | Sat | 0.068 | 0.089 | 0.764 | 574 | 0.445 | 1 |
| Thu | - | Sun | 0.09 | 0.091 | 0.988 | 574 | 0.323 | 1 |
| Tue | - | Fri | -0.11 | 0.091 | -1.207 | 574 | 0.228 | 1 |
| Tue | - | Sat | -0.127 | 0.09 | -1.417 | 574 | 0.157 | 1 |
| Tue | - | Sun | -0.105 | 0.093 | -1.13 | 574 | 0.259 | 1 |
| Tue | - | Wed | 0.032 | 0.094 | 0.335 | 574 | 0.738 | 1 |
| Tue | - | Thu | -0.195 | 0.092 | -2.119 | 574 | 0.035 | 0.725 |

*[* p < 0.05; ** p < 0.01, *** p < 0.001]*

***Supplementary Table B.7.:**  Results of generalized linear model for Z-scored composite risk score for those with a Weak SOW of weekday, age, and gender.

|  |  |  | | **95% Confidence Interval** | | | |  | |  | |  | |
| --- | --- | --- | --- | --- | --- | --- | --- | --- | --- | --- | --- | --- | --- |
| **Effect** | **Estimate** | **SE** | **Lower** | | **Upper** | **β** | **df** | | **t** | | **p** | |  |
| (Intercept) | 0.078 | 0.025 | 0.029 | | 0.127 | 0.000 | 571 | | 3.124 | | 0.002 | |  |
| Male – Female*** | 0.380 | 0.050 | 0.282 | | 0.479 | 0.643 | 571 | | 7.582 | | < .001 | |  |
| Age*** | -0.011 | 0.002 | -0.014 | | -0.007 | -0.233 | 571 | | -6.035 | | < .001 | |  |
| Tue - Mon | -0.086 | 0.087 | -0.256 | | 0.084 | -0.146 | 571 | | -0.993 | | 0.321 | |  |
| Wed - Mon | -0.137 | 0.088 | -0.309 | | 0.035 | -0.232 | 571 | | -1.564 | | 0.118 | |  |
| Thu - Mon | 0.125 | 0.086 | -0.044 | | 0.294 | 0.211 | 571 | | 1.449 | | 0.148 | |  |
| Fri - Mon | -0.037 | 0.085 | -0.204 | | 0.130 | -0.063 | 571 | | -0.439 | | 0.661 | |  |
| Sat - Mon | 0.013 | 0.083 | -0.151 | | 0.177 | 0.022 | 571 | | 0.154 | | 0.878 | |  |
| Sun - Mon | -0.026 | 0.086 | -0.195 | | 0.143 | -0.044 | 571 | | -0.301 | | 0.764 | |  |

*[* p < 0.05; ** p < 0.01, *** p < 0.001]*

***Supplementary Materials Table B.8.:** Results of post-hoc comparisons for Z-scored composite risk score for those with a Weak SOW of weekday, age, and gender.

| **Weekday** |  | **Weekday** | **Difference** | **SE** | **t** | **df** | **p** | **p_bonferroni_** |
| --- | --- | --- | --- | --- | --- | --- | --- | --- |
| Fri | - | Sat | -0.050 | 0.082 | -0.612 | 571 | 0.541 | 1 |
| Fri | - | Sun | -0.011 | 0.084 | -0.136 | 571 | 0.892 | 1 |
| Mon | - | Fri | 0.037 | 0.085 | 0.439 | 571 | 0.661 | 1 |
| Mon | - | Sat | -0.013 | 0.083 | -0.154 | 571 | 0.878 | 1 |
| Mon | - | Sun | 0.026 | 0.086 | 0.301 | 571 | 0.764 | 1 |
| Mon | - | Wed | 0.137 | 0.088 | 1.564 | 571 | 0.118 | 1 |
| Mon | - | Thu | -0.125 | 0.086 | -1.449 | 571 | 0.148 | 1 |
| Mon | - | Tue | 0.086 | 0.087 | 0.993 | 571 | 0.321 | 1 |
| Sat | - | Sun | 0.039 | 0.083 | 0.467 | 571 | 0.641 | 1 |
| Wed | - | Fri | -0.100 | 0.086 | -1.157 | 571 | 0.248 | 1 |
| Wed | - | Sat | -0.150 | 0.085 | -1.772 | 571 | 0.077 | 1 |
| Wed | - | Sun | -0.111 | 0.087 | -1.277 | 571 | 0.202 | 1 |
| Wed | - | Thu | -0.262 | 0.087 | -3.011 | 571 | 0.003 | 0.057 |
| Thu | - | Fri | 0.162 | 0.085 | 1.916 | 571 | 0.056 | 1 |
| Thu | - | Sat | 0.112 | 0.083 | 1.350 | 571 | 0.178 | 1 |
| Thu | - | Sun | 0.151 | 0.085 | 1.763 | 571 | 0.078 | 1 |
| Tue | - | Fri | -0.049 | 0.085 | -0.571 | 571 | 0.569 | 1 |
| Tue | - | Sat | -0.099 | 0.083 | -1.184 | 571 | 0.237 | 1 |
| Tue | - | Sun | -0.060 | 0.086 | -0.698 | 571 | 0.486 | 1 |
| Tue | - | Wed | 0.051 | 0.088 | 0.584 | 571 | 0.559 | 1 |
| Tue | - | Thu | -0.211 | 0.086 | -2.455 | 571 | 0.014 | 0.302 |

*[* p < 0.05; ** p < 0.01, *** p < 0.001]*

**Supplementary Material Table C:** Individual risk measurement descriptives by weekday and Sense of Week (SOW) in Study 1.

|  |  |  | **N** | **Mean** | ***σ*_M_** |
| --- | --- | --- | --- | --- | --- |
| **SOEP** | **Strong, Normal** | *Monday* | 41 | 0.251 | 0.14 |
|  |  | *Tuesday* | 42 | 0.165 | 0.141 |
|  |  | *Wednesday* | 47 | -0.149 | 0.145 |
|  |  | *Thursday* | 37 | -0.296 | 0.162 |
|  |  | *Friday* | 38 | 0.192 | 0.133 |
|  |  | *Saturday* | 34 | -0.174 | 0.186 |
|  |  | *Sunday* | 39 | -0.0519 | 0.164 |
|  | **Weak** | *Monday* | 81 | -0.0338 | 0.112 |
|  |  | *Tuesday* | 80 | -0.0767 | 0.118 |
|  |  | *Wednesday* | 76 | -0.221 | 0.12 |
|  |  | *Thursday* | 84 | 0.22 | 0.107 |
|  |  | *Friday* | 86 | 0.0773 | 0.113 |
|  |  | *Saturday* | 94 | 0.0313 | 0.107 |
|  |  | *Sunday* | 83 | 0.0132 | 0.0958 |
| **DOSPERT** | **Strong, Normal** | *Monday* | 41 | -0.0301 | 0.152 |
|  |  | *Tuesday* | 43 | 0.132 | 0.151 |
|  |  | *Wednesday* | 47 | -0.0772 | 0.154 |
|  |  | *Thursday* | 37 | -0.495 | 0.117 |
|  |  | *Friday* | 38 | 0.179 | 0.174 |
|  |  | *Saturday* | 34 | -0.0112 | 0.161 |
|  |  | *Sunday* | 39 | 0.015 | 0.166 |
|  | **Weak** | *Monday* | 81 | 0.115 | 0.116 |
|  |  | *Tuesday* | 80 | -0.123 | 0.112 |
|  |  | *Wednesday* | 76 | -0.175 | 0.116 |
|  |  | *Thursday* | 84 | 0.206 | 0.104 |
|  |  | *Friday* | 86 | 0.0952 | 0.106 |
|  |  | *Saturday* | 94 | 0.0224 | 0.105 |
|  |  | *Sunday* | 83 | -0.0351 | 0.112 |
| **BEG** | **Strong, Normal** | *Monday* | 41 | 0.0407 | 0.162 |
|  |  | *Tuesday* | 43 | 0.0233 | 0.154 |
|  |  | *Wednesday* | 46 | 0.00678 | 0.132 |
|  |  | *Thursday* | 37 | -0.275 | 0.145 |
|  |  | *Friday* | 36 | -0.142 | 0.16 |
|  |  | *Saturday* | 33 | 0.0175 | 0.157 |
|  |  | *Sunday* | 39 | -0.197 | 0.177 |
|  | **Weak** | *Monday* | 80 | 0.136 | 0.119 |
|  |  | *Tuesday* | 80 | -0.0597 | 0.115 |
|  |  | *Wednesday* | 76 | -0.0865 | 0.108 |
|  |  | *Thursday* | 84 | -0.00556 | 0.113 |
|  |  | *Friday* | 86 | 0.195 | 0.1 |
|  |  | *Saturday* | 93 | 0.195 | 0.109 |
|  |  | *Sunday* | 82 | 0.11 | 0.114 |
| **BART** | **Strong, Normal** | *Monday* | 41 | 0.00711 | 0.131 |
|  |  | *Tuesday* | 43 | 0.292 | 0.164 |
|  |  | *Wednesday* | 47 | -0.0278 | 0.153 |
|  |  | *Thursday* | 37 | 0.248 | 0.132 |
|  |  | *Friday* | 38 | -0.262 | 0.148 |
|  |  | *Saturday* | 34 | -0.158 | 0.154 |
|  |  | *Sunday* | 39 | 0.000184 | 0.177 |
|  | **Weak** | *Monday* | 81 | -0.041 | 0.108 |
|  |  | *Tuesday* | 80 | -0.00473 | 0.117 |
|  |  | *Wednesday* | 76 | -0.0762 | 0.132 |
|  |  | *Thursday* | 84 | -0.0909 | 0.0951 |
|  |  | *Friday* | 86 | 0.0356 | 0.107 |
|  |  | *Saturday* | 94 | 0.0125 | 0.104 |
|  |  | *Sunday* | 83 | 0.101 | 0.117 |

**Supplementary Material Figure D:** The mean scores for each of the four main risk measurements across participants, separated out between a weak (rating of 1 or 2 on a scale of 1 to 5) versus normal or weak (rating of 3, 4, or 5 on the same scale) sense of the week, in Study 1, all normalized using z-scoring. A) SOEP General, B) DOSPERT General, C) BEG, D) Normalized BART scores, scored as per Lejuez et al. (2002) methodology. Error bars represent +/- SE.

| 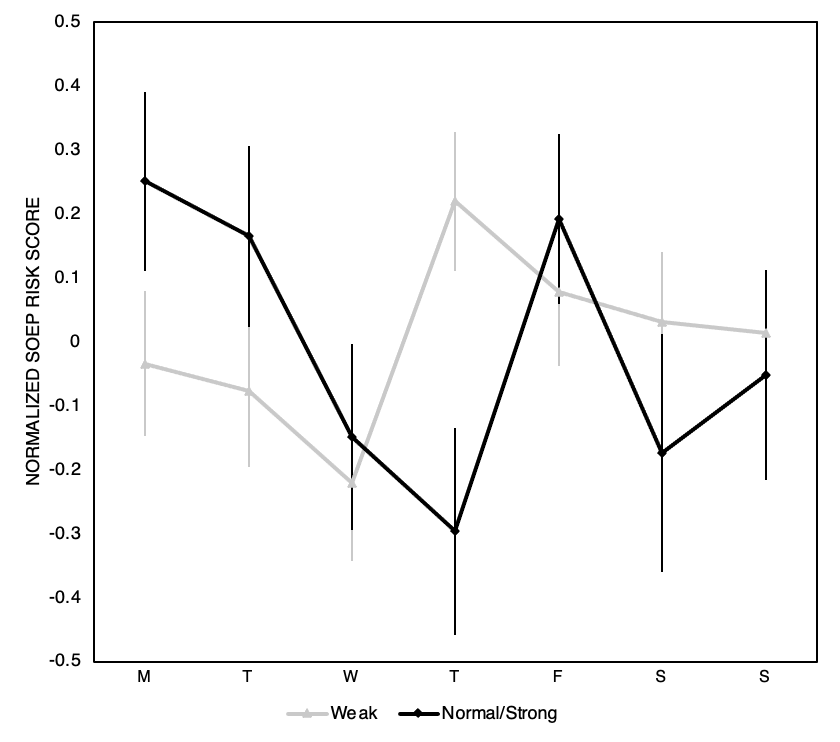 | 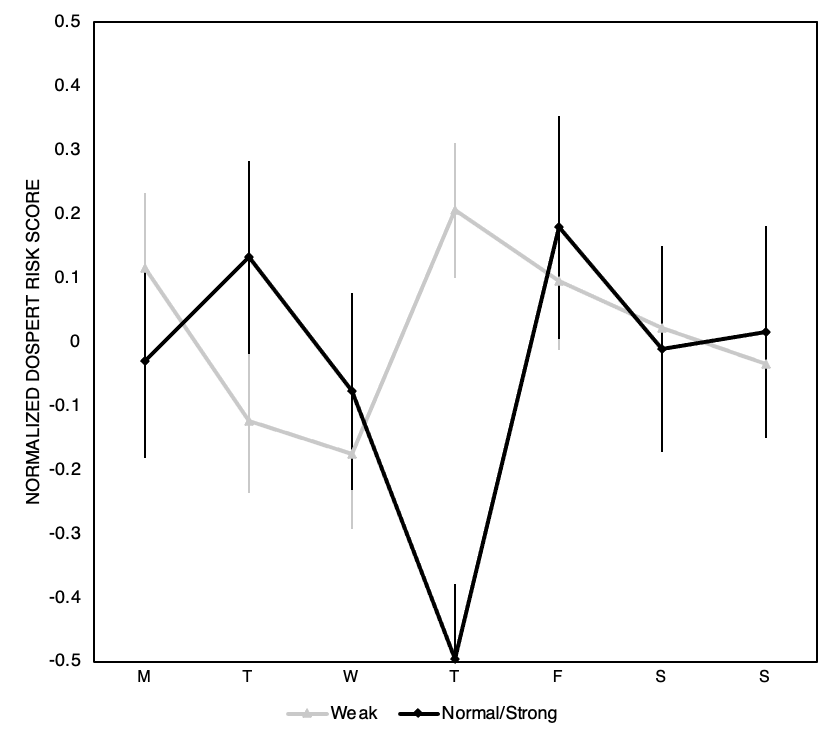 |
| --- | --- |
| 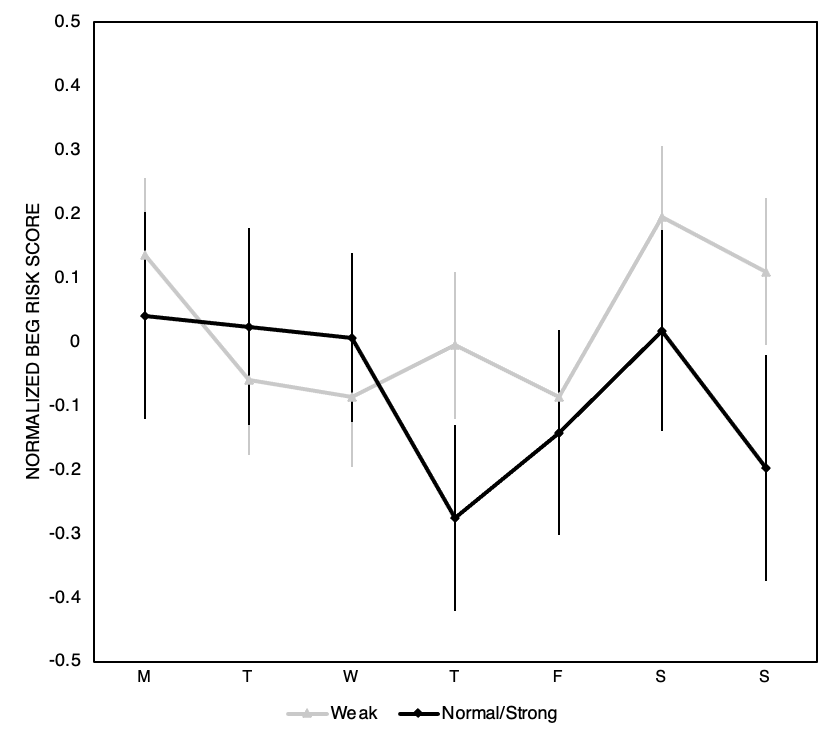 | 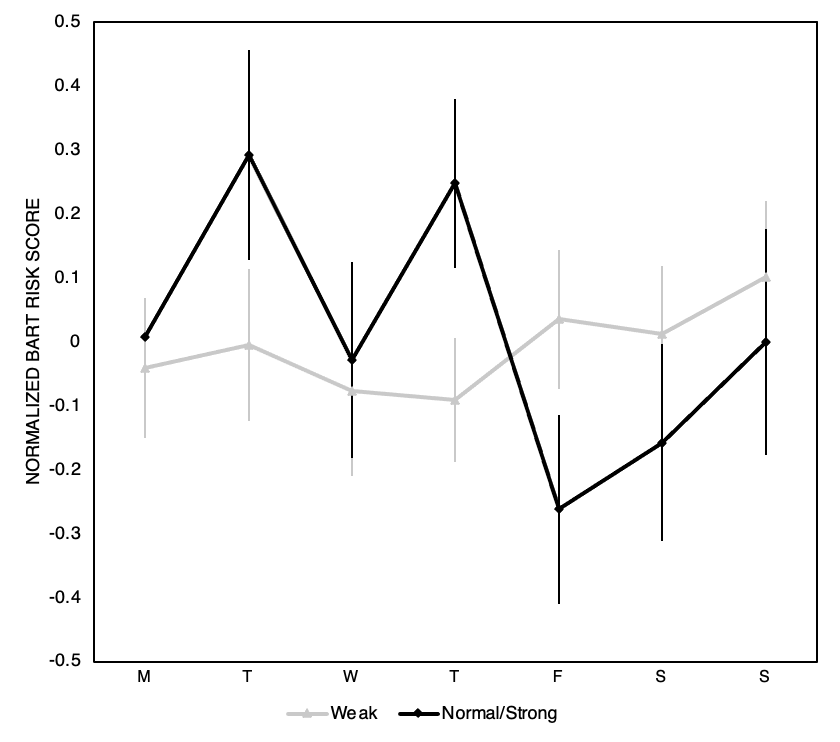 |

***Supplementary Table D.1.:**  Results of generalized linear model for Z-scored SOEP for those with a Normal/Strong SOW of weekday only.

|  |  |  | **95% Confidence Interval** | | |  |  |  |
| --- | --- | --- | --- | --- | --- | --- | --- | --- |
| **Effect** | **Estimate** | **SE** | **Lower** | **Upper** | **β** | **df** | **t** | **p** |
| (Intercept) | -0.004 | 0.06 | -0.122 | 0.113 | 0 | 271 | -0.069 | 0.945 |
| Tue - Mon | -0.089 | 0.217 | -0.517 | 0.339 | -0.089 | 271 | -0.409 | 0.683 |
| Wed - Mon | -0.412 | 0.212 | -0.828 | 0.005 | -0.412 | 271 | -1.944 | 0.053 |
| Thu - Mon* | -0.563 | 0.225 | -1.005 | -0.121 | -0.563 | 271 | -2.505 | 0.013 |
| Fri - Mon | -0.061 | 0.223 | -0.5 | 0.378 | -0.061 | 271 | -0.273 | 0.785 |
| Sat - Mon | -0.437 | 0.23 | -0.889 | 0.015 | -0.437 | 271 | -1.902 | 0.058 |
| Sun - Mon | -0.312 | 0.222 | -0.748 | 0.125 | -0.312 | 271 | -1.407 | 0.161 |

***Supplementary Materials Table D.2.:** Results of post-hoc comparisons for Z-scored SOEP risk score for those with a Normal/Strong SOW of weekday only.

| **Weekday** |  | **Weekday** | **Difference** | **SE** | **t** | **df** | **p** | **p_bonferroni_** |
| --- | --- | --- | --- | --- | --- | --- | --- | --- |
| Fri | - | Sat | 0.376 | 0.234 | 1.608 | 271 | 0.109 | 1 |
| Fri | - | Sun | 0.251 | 0.226 | 1.11 | 271 | 0.268 | 1 |
| Mon | - | Fri | 0.061 | 0.223 | 0.273 | 271 | 0.785 | 1 |
| Mon | - | Sat | 0.437 | 0.23 | 1.902 | 271 | 0.058 | 1 |
| Mon | - | Sun | 0.312 | 0.222 | 1.407 | 271 | 0.161 | 1 |
| Mon | - | Wed | 0.412 | 0.212 | 1.944 | 271 | 0.053 | 1 |
| Mon | - | Thu | 0.563 | 0.225 | 2.505 | 271 | 0.013 | 0.269 |
| Mon | - | Tue | 0.089 | 0.217 | 0.409 | 271 | 0.683 | 1 |
| Sat | - | Sun | -0.125 | 0.232 | -0.539 | 271 | 0.59 | 1 |
| Wed | - | Fri | -0.351 | 0.216 | -1.622 | 271 | 0.106 | 1 |
| Wed | - | Sat | 0.025 | 0.223 | 0.114 | 271 | 0.91 | 1 |
| Wed | - | Sun | -0.1 | 0.215 | -0.466 | 271 | 0.642 | 1 |
| Wed | - | Thu | 0.151 | 0.218 | 0.694 | 271 | 0.488 | 1 |
| Thu | - | Fri | -0.502 | 0.229 | -2.193 | 271 | 0.029 | 0.612 |
| Thu | - | Sat | -0.126 | 0.235 | -0.535 | 271 | 0.593 | 1 |
| Thu | - | Sun | -0.251 | 0.227 | -1.104 | 271 | 0.27 | 1 |
| Tue | - | Fri | -0.028 | 0.222 | -0.126 | 271 | 0.9 | 1 |
| Tue | - | Sat | 0.348 | 0.228 | 1.523 | 271 | 0.129 | 1 |
| Tue | - | Sun | 0.223 | 0.22 | 1.011 | 271 | 0.313 | 1 |
| Tue | - | Wed | 0.323 | 0.21 | 1.534 | 271 | 0.126 | 1 |
| Tue | - | Thu | 0.474 | 0.223 | 2.122 | 271 | 0.035 | 0.73 |

*[* p < 0.05; ** p < 0.01, *** p < 0.001]*

***Supplementary Table D.3.:**  Results of generalized linear model for Z-scored SOEP for those with a Normal/Strong SOW of weekday, age, and gender.

|  |  |  | **95% Confidence Interval** | | |  |  |  |
| --- | --- | --- | --- | --- | --- | --- | --- | --- |
| **Effect** | **Estimate** | **SE** | **Lower** | **Upper** | **β** | **df** | **t** | **p** |
| (Intercept) | 0.04 | 0.06 | -0.079 | 0.158 | 0 | 269 | 0.663 | 0.508 |
| Male - Female | 0.387 | 0.122 | 0.146 | 0.627 | 0.387 | 269 | 3.168 | 0.002 |
| Tue - Mon | -0.041 | 0.214 | -0.462 | 0.379 | -0.041 | 269 | -0.193 | 0.847 |
| Wed - Mon | -0.355 | 0.208 | -0.765 | 0.055 | -0.355 | 269 | -1.704 | 0.089 |
| Thu – Mon* | -0.481 | 0.221 | -0.917 | -0.045 | -0.481 | 269 | -2.173 | 0.031 |
| Fri - Mon | -0.103 | 0.219 | -0.534 | 0.329 | -0.103 | 269 | -0.469 | 0.64 |
| Sat – Mon* | -0.459 | 0.225 | -0.903 | -0.015 | -0.459 | 269 | -2.037 | 0.043 |
| Sun - Mon | -0.322 | 0.217 | -0.749 | 0.106 | -0.322 | 269 | -1.481 | 0.14 |
| Age | -0.01 | 0.005 | -0.021 | 1.56E-04 | -0.114 | 269 | -1.939 | 0.054 |

*[* p < 0.05; ** p < 0.01, *** p < 0.001]*

***Supplementary Materials Table D.4.:** Results of post-hoc comparisons for Z-scored SOEP risk score for those with a Normal/Strong SOW of weekday, age, and gender.

| **Weekday** |  | **Weekday** | **Difference** | **SE** | **t** | **df** | **p** | **p_bonferroni_** |
| --- | --- | --- | --- | --- | --- | --- | --- | --- |
| Fri | - | Sat | 0.356 | 0.229 | 1.554 | 269 | 0.121 | 1 |
| Fri | - | Sun | 0.219 | 0.222 | 0.989 | 269 | 0.324 | 1 |
| Mon | - | Fri | 0.103 | 0.219 | 0.469 | 269 | 0.64 | 1 |
| Mon | - | Sat | 0.459 | 0.225 | 2.037 | 269 | 0.043 | 0.896 |
| Mon | - | Sun | 0.322 | 0.217 | 1.481 | 269 | 0.14 | 1 |
| Mon | - | Wed | 0.355 | 0.208 | 1.704 | 269 | 0.089 | 1 |
| Mon | - | Thu | 0.481 | 0.221 | 2.173 | 269 | 0.031 | 0.644 |
| Mon | - | Tue | 0.041 | 0.214 | 0.193 | 269 | 0.847 | 1 |
| Sat | - | Sun | -0.137 | 0.228 | -0.602 | 269 | 0.547 | 1 |
| Wed | - | Fri | -0.252 | 0.214 | -1.181 | 269 | 0.239 | 1 |
| Wed | - | Sat | 0.104 | 0.22 | 0.473 | 269 | 0.637 | 1 |
| Wed | - | Sun | -0.033 | 0.211 | -0.157 | 269 | 0.875 | 1 |
| Wed | - | Thu | 0.126 | 0.214 | 0.59 | 269 | 0.556 | 1 |
| Thu | - | Fri | -0.378 | 0.227 | -1.668 | 269 | 0.097 | 1 |
| Thu | - | Sat | -0.022 | 0.233 | -0.095 | 269 | 0.924 | 1 |
| Thu | - | Sun | -0.159 | 0.224 | -0.71 | 269 | 0.478 | 1 |
| Tue | - | Fri | 0.061 | 0.219 | 0.281 | 269 | 0.779 | 1 |
| Tue | - | Sat | 0.418 | 0.225 | 1.858 | 269 | 0.064 | 1 |
| Tue | - | Sun | 0.281 | 0.217 | 1.295 | 269 | 0.196 | 1 |
| Tue | - | Wed | 0.314 | 0.207 | 1.52 | 269 | 0.13 | 1 |
| Tue | - | Thu | 0.44 | 0.219 | 2.007 | 269 | 0.046 | 0.96 |

*[* p < 0.05; ** p < 0.01, *** p < 0.001]*

***Supplementary Table D.5.:**  Results of generalized linear model for Z-scored SOEP for those with a Weak SOW of weekday only.

|  |  |  | **95% Confidence Interval** | | |  |  |  |
| --- | --- | --- | --- | --- | --- | --- | --- | --- |
| **Effect** | **Estimate** | **SE** | **Lower** | **Upper** | **β** | **df** | **t** | **p** |
| (Intercept) | -0.004 | 0.041 | -0.086 | 0.077 | 0 | 577 | -0.108 | 0.914 |
| Tue - Mon | -0.042 | 0.157 | -0.351 | 0.266 | -0.042 | 577 | -0.27 | 0.787 |
| Wed - Mon | -0.185 | 0.159 | -0.498 | 0.128 | -0.185 | 577 | -1.161 | 0.246 |
| Thu - Mon | 0.251 | 0.155 | -0.054 | 0.556 | 0.251 | 577 | 1.615 | 0.107 |
| Fri - Mon | 0.11 | 0.154 | -0.194 | 0.413 | 0.11 | 577 | 0.711 | 0.477 |
| Sat - Mon | 0.064 | 0.151 | -0.233 | 0.361 | 0.064 | 577 | 0.426 | 0.671 |
| Sun - Mon | 0.047 | 0.156 | -0.259 | 0.353 | 0.047 | 577 | 0.299 | 0.765 |

*[* p < 0.05; ** p < 0.01, *** p < 0.001]*

***Supplementary Materials Table D.6.:** Results of post-hoc comparisons for Z-scored SOEP risk score for those with a Weak SOW of weekday only.

| **Weekday** |  | **Weekday** | **Difference** | **SE** | **t** | **df** | **p** | **p_bonferroni_** |
| --- | --- | --- | --- | --- | --- | --- | --- | --- |
| Fri | - | Sat | 0.045 | 0.149 | 0.306 | 577 | 0.76 | 1 |
| Fri | - | Sun | 0.063 | 0.154 | 0.412 | 577 | 0.68 | 1 |
| Mon | - | Fri | -0.11 | 0.154 | -0.711 | 577 | 0.477 | 1 |
| Mon | - | Sat | -0.064 | 0.151 | -0.426 | 577 | 0.671 | 1 |
| Mon | - | Sun | -0.047 | 0.156 | -0.299 | 577 | 0.765 | 1 |
| Mon | - | Wed | 0.185 | 0.159 | 1.161 | 577 | 0.246 | 1 |
| Mon | - | Thu | -0.251 | 0.155 | -1.615 | 577 | 0.107 | 1 |
| Mon | - | Tue | 0.042 | 0.157 | 0.27 | 577 | 0.787 | 1 |
| Sat | - | Sun | 0.018 | 0.15 | 0.119 | 577 | 0.906 | 1 |
| Wed | - | Fri | -0.295 | 0.157 | -1.877 | 577 | 0.061 | 1 |
| Wed | - | Sat | -0.249 | 0.154 | -1.62 | 577 | 0.106 | 1 |
| Wed | - | Sun | -0.231 | 0.158 | -1.461 | 577 | 0.144 | 1 |
| Wed | - | Thu | -0.436 | 0.158 | -2.76 | 577 | 0.006 | 0.125 |
| Thu | - | Fri | 0.141 | 0.153 | 0.922 | 577 | 0.357 | 1 |
| Thu | - | Sat | 0.187 | 0.15 | 1.246 | 577 | 0.213 | 1 |
| Thu | - | Sun | 0.204 | 0.154 | 1.324 | 577 | 0.186 | 1 |
| Tue | - | Fri | -0.152 | 0.155 | -0.983 | 577 | 0.326 | 1 |
| Tue | - | Sat | -0.107 | 0.152 | -0.704 | 577 | 0.482 | 1 |
| Tue | - | Sun | -0.089 | 0.156 | -0.569 | 577 | 0.569 | 1 |
| Tue | - | Wed | 0.142 | 0.16 | 0.891 | 577 | 0.373 | 1 |
| Tue | - | Thu | -0.293 | 0.156 | -1.883 | 577 | 0.06 | 1 |

*[* p < 0.05; ** p < 0.01, *** p < 0.001]*

***Supplementary Table D.7.:**  Results of generalized linear model for Z-scored SOEP for those with a Weak SOW of weekday, age, and gender.

|  |  |  | **95% Confidence Interval** | | |  |  |  |
| --- | --- | --- | --- | --- | --- | --- | --- | --- |
| **Effect** | **Estimate** | **SE** | **Lower** | **Upper** | **β** | **df** | **t** | **p** |
| (Intercept) | 0.102 | 0.043 | 0.017 | 0.187 | 0 | 574 | 2.355 | 0.019 |
| Male – Female*** | 0.518 | 0.087 | 0.347 | 0.688 | 0.518 | 574 | 5.966 | < .001 |
| Age*** | -0.016 | 0.003 | -0.022 | -0.01 | -0.205 | 574 | -5.199 | < .001 |
| Tue - Mon | -0.002 | 0.15 | -0.297 | 0.292 | -0.002 | 574 | -0.015 | 0.988 |
| Wed - Mon | -0.173 | 0.152 | -0.471 | 0.125 | -0.173 | 574 | -1.14 | 0.255 |
| Thu – Mon* | 0.304 | 0.149 | 0.011 | 0.597 | 0.304 | 574 | 2.04 | 0.042 |
| Fri - Mon | 0.062 | 0.147 | -0.227 | 0.352 | 0.062 | 574 | 0.422 | 0.673 |
| Sat - Mon | 0.065 | 0.144 | -0.218 | 0.348 | 0.065 | 574 | 0.449 | 0.653 |
| Sun - Mon | 0.024 | 0.149 | -0.268 | 0.316 | 0.024 | 574 | 0.16 | 0.873 |

*[* p < 0.05; ** p < 0.01, *** p < 0.001]*

***Supplementary Materials Table D.8.:** Results of post-hoc comparisons for Z-scored SOEP risk score for those with a Weak SOW of weekday, age, and gender.

| **Weekday** |  | **Weekday** | **Difference** | **SE** | **t** | **df** | **p** | **p_bonferroni_** |
| --- | --- | --- | --- | --- | --- | --- | --- | --- |
| Fri | - | Sat | -0.002 | 0.142 | -0.017 | 574 | 0.986 | 1 |
| Fri | - | Sun | 0.038 | 0.146 | 0.263 | 574 | 0.792 | 1 |
| Mon | - | Fri | -0.062 | 0.147 | -0.422 | 574 | 0.673 | 1 |
| Mon | - | Sat | -0.065 | 0.144 | -0.449 | 574 | 0.653 | 1 |
| Mon | - | Sun | -0.024 | 0.149 | -0.16 | 574 | 0.873 | 1 |
| Mon | - | Wed | 0.173 | 0.152 | 1.14 | 574 | 0.255 | 1 |
| Mon | - | Thu | -0.304 | 0.149 | -2.04 | 574 | 0.042 | 0.877 |
| Mon | - | Tue | 0.002 | 0.15 | 0.015 | 574 | 0.988 | 1 |
| Sat | - | Sun | 0.041 | 0.143 | 0.286 | 574 | 0.775 | 1 |
| Wed | - | Fri | -0.235 | 0.15 | -1.572 | 574 | 0.117 | 1 |
| Wed | - | Sat | -0.238 | 0.147 | -1.622 | 574 | 0.105 | 1 |
| Wed | - | Sun | -0.197 | 0.151 | -1.305 | 574 | 0.192 | 1 |
| Wed | - | Thu* | -0.477 | 0.151 | -3.16 | 574 | 0.002 | 0.035 |
| Thu | - | Fri | 0.242 | 0.147 | 1.647 | 574 | 0.1 | 1 |
| Thu | - | Sat | 0.239 | 0.144 | 1.668 | 574 | 0.096 | 1 |
| Thu | - | Sun | 0.28 | 0.148 | 1.896 | 574 | 0.058 | 1 |
| Tue | - | Fri | -0.064 | 0.148 | -0.436 | 574 | 0.663 | 1 |
| Tue | - | Sat | -0.067 | 0.145 | -0.463 | 574 | 0.644 | 1 |
| Tue | - | Sun | -0.026 | 0.149 | -0.174 | 574 | 0.862 | 1 |
| Tue | - | Wed | 0.171 | 0.152 | 1.123 | 574 | 0.262 | 1 |
| Tue | - | Thu | -0.306 | 0.149 | -2.055 | 574 | 0.04 | 0.846 |

*[* p < 0.05; ** p < 0.01, *** p < 0.001]*

***Supplementary Table D.9.:**  Results of generalized linear model for Z-scored DOSPERT for those with a Normal/Strong SOW of weekday only.

|  |  |  | **95% Confidence Interval** | | |  |  |  |
| --- | --- | --- | --- | --- | --- | --- | --- | --- |
| **Effect** | **Estimate** | **SE** | **Lower** | **Upper** | **β** | **df** | **t** | **p** |
| (Intercept) | -0.004 | 0.06 | -0.121 | 0.114 | 0 | 272 | -0.059 | 0.953 |
| Tue - Mon | 0.164 | 0.216 | -0.261 | 0.59 | 0.164 | 272 | 0.76 | 0.448 |
| Wed - Mon | -0.048 | 0.212 | -0.464 | 0.369 | -0.048 | 272 | -0.225 | 0.822 |
| Thu - Mon* | -0.47 | 0.225 | -0.912 | -0.028 | -0.47 | 272 | -2.095 | 0.037 |
| Fri - Mon | 0.211 | 0.223 | -0.228 | 0.65 | 0.211 | 272 | 0.947 | 0.344 |
| Sat - Mon | 0.019 | 0.23 | -0.433 | 0.471 | 0.019 | 272 | 0.083 | 0.934 |
| Sun - Mon | 0.046 | 0.222 | -0.391 | 0.482 | 0.046 | 272 | 0.206 | 0.837 |

*[* p < 0.05; ** p < 0.01, *** p < 0.001]*

***Supplementary Materials Table D.10.:** Results of post-hoc comparisons for Z-scored DOSPERT risk score for those with a Normal/Strong SOW of weekday only.

| **Weekday** |  | **Weekday** | **Difference** | **SE** | **t** | **df** | **p** | **p_bonferroni_** |
| --- | --- | --- | --- | --- | --- | --- | --- | --- |
| Fri | - | Sat | 0.192 | 0.234 | 0.822 | 272 | 0.412 | 1 |
| Fri | - | Sun | 0.166 | 0.226 | 0.734 | 272 | 0.464 | 1 |
| Mon | - | Fri | -0.211 | 0.223 | -0.947 | 272 | 0.344 | 1 |
| Mon | - | Sat | -0.019 | 0.23 | -0.083 | 272 | 0.934 | 1 |
| Mon | - | Sun | -0.046 | 0.222 | -0.206 | 272 | 0.837 | 1 |
| Mon | - | Wed | 0.048 | 0.212 | 0.225 | 272 | 0.822 | 1 |
| Mon | - | Thu | 0.47 | 0.225 | 2.095 | 272 | 0.037 | 0.78 |
| Mon | - | Tue | -0.164 | 0.216 | -0.76 | 272 | 0.448 | 1 |
| Sat | - | Sun | -0.026 | 0.232 | -0.114 | 272 | 0.909 | 1 |
| Wed | - | Fri | -0.259 | 0.216 | -1.199 | 272 | 0.232 | 1 |
| Wed | - | Sat | -0.067 | 0.223 | -0.299 | 272 | 0.765 | 1 |
| Wed | - | Sun | -0.093 | 0.215 | -0.435 | 272 | 0.664 | 1 |
| Wed | - | Thu | 0.423 | 0.218 | 1.942 | 272 | 0.053 | 1 |
| Thu | - | Fri | -0.682 | 0.229 | -2.98 | 272 | 0.003 | 0.066 |
| Thu | - | Sat | -0.489 | 0.235 | -2.08 | 272 | 0.038 | 0.807 |
| Thu | - | Sun | -0.516 | 0.227 | -2.27 | 272 | 0.024 | 0.504 |
| Tue | - | Fri | -0.047 | 0.22 | -0.213 | 272 | 0.832 | 1 |
| Tue | - | Sat | 0.145 | 0.227 | 0.639 | 272 | 0.523 | 1 |
| Tue | - | Sun | 0.119 | 0.219 | 0.543 | 272 | 0.588 | 1 |
| Tue | - | Wed | 0.212 | 0.209 | 1.015 | 272 | 0.311 | 1 |
| Tue | - | Thu | 0.635 | 0.222 | 2.858 | 272 | 0.005 | 0.096 |

*[* p < 0.05; ** p < 0.01, *** p < 0.001]*

***Supplementary Materials Table D.11.:** Results of generalized linear model for Z-scored DOSPERT risk score for those with a Normal/Strong SOW of weekday, age, and gender.

|  |  |  | **95% Confidence Interval** | | |  |  |  |
| --- | --- | --- | --- | --- | --- | --- | --- | --- |
| **Effect** | **Estimate** | **SE** | **Lower** | **Upper** | **β** | **df** | **t** | **p** |
| (Intercept) | 0.035 | 0.060 | -0.083 | 0.152 | 0.000 | 270 | 0.583 | 0.560 |
| Age*** | -0.018 | 0.005 | -0.028 | -0.007 | -0.196 | 270 | -3.362 | < .001 |
| Male – Female** | 0.340 | 0.121 | 0.102 | 0.578 | 0.340 | 270 | 2.810 | 0.005 |
| Tue - Mon | 0.214 | 0.211 | -0.201 | 0.629 | 0.214 | 270 | 1.016 | 0.311 |
| Wed - Mon | 0.025 | 0.206 | -0.381 | 0.432 | 0.025 | 270 | 0.123 | 0.902 |
| Thu - Mon | -0.386 | 0.219 | -0.818 | 0.046 | -0.386 | 270 | -1.757 | 0.080 |
| Fri - Mon | 0.167 | 0.217 | -0.260 | 0.595 | 0.167 | 270 | 0.771 | 0.441 |
| Sat - Mon | -0.010 | 0.223 | -0.450 | 0.430 | -0.010 | 270 | -0.046 | 0.963 |
| Sun - Mon | 0.035 | 0.215 | -0.389 | 0.459 | 0.035 | 270 | 0.163 | 0.871 |

*[* p < 0.05; ** p < 0.01, *** p < 0.001]*

***Supplementary Materials Table D.12.:** Results of post-hoc comparisons for Z-scored DOSPERT risk score for those with a Normal/Strong SOW of weekday, age, and gender.

| **Weekday** |  |  | **Difference** | **SE** | **t** | **df** | **p** | **p_bonferroni_** |
| --- | --- | --- | --- | --- | --- | --- | --- | --- |
| Fri | - | Sat | 0.178 | 0.227 | 0.782 | 270 | 0.435 | 1 |
| Fri | - | Sun | 0.132 | 0.220 | 0.602 | 270 | 0.547 | 1 |
| Mon | - | Fri | -0.167 | 0.217 | -0.771 | 270 | 0.441 | 1 |
| Mon | - | Sat | 0.010 | 0.223 | 0.046 | 270 | 0.963 | 1 |
| Mon | - | Sun | -0.035 | 0.215 | -0.163 | 270 | 0.871 | 1 |
| Mon | - | Wed | -0.025 | 0.207 | -0.123 | 270 | 0.902 | 1 |
| Mon | - | Thu | 0.386 | 0.220 | 1.757 | 270 | 0.08 | 1 |
| Mon | - | Tue | -0.214 | 0.211 | -1.016 | 270 | 0.311 | 1 |
| Sat | - | Sun | -0.045 | 0.226 | -0.201 | 270 | 0.841 | 1 |
| Wed | - | Fri | -0.142 | 0.212 | -0.670 | 270 | 0.503 | 1 |
| Wed | - | Sat | 0.036 | 0.218 | 0.164 | 270 | 0.87 | 1 |
| Wed | - | Sun | -0.010 | 0.210 | -0.046 | 270 | 0.963 | 1 |
| Wed | - | Thu | 0.411 | 0.212 | 1.939 | 270 | 0.053 | 1 |
| Thu | - | Fri | -0.553 | 0.225 | -2.459 | 270 | 0.015 | 0.306 |
| Thu | - | Sat | -0.375 | 0.231 | -1.629 | 270 | 0.105 | 1 |
| Thu | - | Sun | -0.421 | 0.222 | -1.892 | 270 | 0.06 | 1 |
| Tue | - | Fri | 0.047 | 0.216 | 0.216 | 270 | 0.829 | 1 |
| Tue | - | Sat | 0.224 | 0.222 | 1.011 | 270 | 0.313 | 1 |
| Tue | - | Sun | 0.179 | 0.214 | 0.837 | 270 | 0.403 | 1 |
| Tue | - | Wed | 0.189 | 0.204 | 0.927 | 270 | 0.355 | 1 |
| Tue | - | Thu | 0.600 | 0.216 | 2.776 | 270 | 0.006 | 0.124 |

*[* p < 0.05; ** p < 0.01, *** p < 0.001]*

***Supplementary Table D.13.:**  Results of generalized linear model for Z-scored DOSPERT for those with a Weak SOW of weekday only.

|  |  |  | **95% Confidence Interval** | | |  |  |  |
| --- | --- | --- | --- | --- | --- | --- | --- | --- |
| **Effect** | **Estimate** | **SE** | **Lower** | **Upper** | **β** | **df** | **t** | **p** |
| (Intercept) | -0.003 | 0.041 | -0.085 | 0.078 | 0 | 577 | -0.085 | 0.933 |
| Tue - Mon | -0.236 | 0.157 | -0.545 | 0.073 | -0.236 | 577 | -1.501 | 0.134 |
| Wed - Mon | -0.287 | 0.159 | -0.6 | 0.026 | -0.287 | 577 | -1.804 | 0.072 |
| Thu - Mon | 0.091 | 0.155 | -0.214 | 0.396 | 0.091 | 577 | 0.587 | 0.557 |
| Fri - Mon | -0.019 | 0.154 | -0.323 | 0.284 | -0.019 | 577 | -0.125 | 0.901 |
| Sat - Mon | -0.092 | 0.151 | -0.389 | 0.206 | -0.092 | 577 | -0.605 | 0.545 |
| Sun - Mon | -0.149 | 0.156 | -0.455 | 0.157 | -0.149 | 577 | -0.954 | 0.34 |

*[* p < 0.05; ** p < 0.01, *** p < 0.001]*

***Supplementary Materials Table D.14.:** Results of post-hoc comparisons for Z-scored SOEP risk score for those with a Weak SOW of weekday only.

|  |  | **Weekday** | **Difference** | **SE** | **t** | **df** | **p** | **p_bonferroni_** |
| --- | --- | --- | --- | --- | --- | --- | --- | --- |
| Fri | - | Sat | 0.072 | 0.149 | 0.486 | 577 | 0.627 | 1 |
| Fri | - | Sun | 0.129 | 0.153 | 0.843 | 577 | 0.399 | 1 |
| Mon | - | Fri | 0.019 | 0.154 | 0.125 | 577 | 0.901 | 1 |
| Mon | - | Sat | 0.092 | 0.151 | 0.605 | 577 | 0.545 | 1 |
| Mon | - | Sun | 0.149 | 0.156 | 0.954 | 577 | 0.34 | 1 |
| Mon | - | Wed | 0.287 | 0.159 | 1.804 | 577 | 0.072 | 1 |
| Mon | - | Thu | -0.091 | 0.155 | -0.587 | 577 | 0.557 | 1 |
| Mon | - | Tue | 0.236 | 0.157 | 1.501 | 577 | 0.134 | 1 |
| Sat | - | Sun | 0.057 | 0.15 | 0.38 | 577 | 0.704 | 1 |
| Wed | - | Fri | -0.268 | 0.157 | -1.707 | 577 | 0.088 | 1 |
| Wed | - | Sat | -0.196 | 0.154 | -1.273 | 577 | 0.204 | 1 |
| Wed | - | Sun | -0.139 | 0.158 | -0.876 | 577 | 0.382 | 1 |
| Wed | - | Thu | -0.379 | 0.158 | -2.397 | 577 | 0.017 | 0.354 |
| Thu | - | Fri | 0.11 | 0.153 | 0.722 | 577 | 0.471 | 1 |
| Thu | - | Sat | 0.183 | 0.15 | 1.22 | 577 | 0.223 | 1 |
| Thu | - | Sun | 0.24 | 0.154 | 1.554 | 577 | 0.121 | 1 |
| Tue | - | Fri | -0.217 | 0.155 | -1.399 | 577 | 0.162 | 1 |
| Tue | - | Sat | -0.144 | 0.152 | -0.952 | 577 | 0.342 | 1 |
| Tue | - | Sun | -0.087 | 0.156 | -0.559 | 577 | 0.577 | 1 |
| Tue | - | Wed | 0.051 | 0.16 | 0.321 | 577 | 0.748 | 1 |
| Tue | - | Thu | -0.327 | 0.156 | -2.1 | 577 | 0.036 | 0.76 |

*[* p < 0.05; ** p < 0.01, *** p < 0.001]*

***Supplementary Table D.15:**  Results of generalized linear model for Z-scored DOSPERT for those with a Weak SOW of weekday, age, and gender.

|  |  |  | **95% Confidence Interval** | | |  |  |  |
| --- | --- | --- | --- | --- | --- | --- | --- | --- |
| **Effect** | **Estimate** | **SE** | **Lower** | **Upper** | **β** | **df** | **t** | **p** |
| (Intercept) | 0.110 | 0.043 | 0.026 | 0.194 | 0.000 | 574 | 2.584 | 0.01 |
| Age*** | -0.019 | 0.003 | -0.025 | -0.013 | -0.243 | 574 | -6.249 | < .001 |
| Male – Female*** | 0.548 | 0.086 | 0.380 | 0.717 | 0.548 | 574 | 6.404 | < .001 |
| Tue - Mon | -0.194 | 0.148 | -0.484 | 0.097 | -0.194 | 574 | -1.310 | 0.191 |
| Wed - Mon | -0.278 | 0.150 | -0.572 | 0.016 | -0.278 | 574 | -1.855 | 0.064 |
| Thu - Mon | 0.152 | 0.147 | -0.136 | 0.441 | 0.152 | 574 | 1.037 | 0.300 |
| Fri - Mon | -0.075 | 0.145 | -0.361 | 0.210 | -0.075 | 574 | -0.519 | 0.604 |
| Sat - Mon | -0.093 | 0.142 | -0.372 | 0.186 | -0.093 | 574 | -0.654 | 0.514 |
| Sun - Mon | -0.178 | 0.147 | -0.466 | 0.110 | -0.178 | 574 | -1.213 | 0.226 |

*[* p < 0.05; ** p < 0.01, *** p < 0.001]*

***Supplementary Materials Table D.16.:** Results of post-hoc comparisons for Z-scored DOSPERT risk score for those with a Weak SOW of weekday, age, and gender.

| **Weekday** |  | **Weekday** | **Difference** | **SE** | **t** | **df** | **p** | **p_bonferroni_** |
| --- | --- | --- | --- | --- | --- | --- | --- | --- |
| Fri | - | Sat | 0.017 | 0.140 | 0.124 | 574 | 0.901 | 1 |
| Fri | - | Sun | 0.102 | 0.144 | 0.710 | 574 | 0.478 | 1 |
| Mon | - | Fri | 0.075 | 0.145 | 0.519 | 574 | 0.604 | 1 |
| Mon | - | Sat | 0.093 | 0.142 | 0.654 | 574 | 0.514 | 1 |
| Mon | - | Sun | 0.178 | 0.147 | 1.213 | 574 | 0.226 | 1 |
| Mon | - | Wed | 0.278 | 0.150 | 1.855 | 574 | 0.064 | 1 |
| Mon | - | Thu | -0.152 | 0.147 | -1.037 | 574 | 0.3 | 1 |
| Mon | - | Tue | 0.194 | 0.148 | 1.310 | 574 | 0.191 | 1 |
| Sat | - | Sun | 0.085 | 0.141 | 0.602 | 574 | 0.548 | 1 |
| Wed | - | Fri | -0.202 | 0.148 | -1.371 | 574 | 0.171 | 1 |
| Wed | - | Sat | -0.185 | 0.145 | -1.280 | 574 | 0.201 | 1 |
| Wed | - | Sun | -0.100 | 0.149 | -0.673 | 574 | 0.501 | 1 |
| Wed | - | Thu | -0.430 | 0.149 | -2.889 | 574 | 0.004 | 0.084 |
| Thu | - | Fri | 0.228 | 0.145 | 1.573 | 574 | 0.116 | 1 |
| Thu | - | Sat | 0.245 | 0.142 | 1.733 | 574 | 0.084 | 1 |
| Thu | - | Sun | 0.330 | 0.146 | 2.264 | 574 | 0.024 | 0.503 |
| Tue | - | Fri | -0.118 | 0.146 | -0.810 | 574 | 0.418 | 1 |
| Tue | - | Sat | -0.101 | 0.143 | -0.707 | 574 | 0.48 | 1 |
| Tue | - | Sun | -0.016 | 0.147 | -0.108 | 574 | 0.914 | 1 |
| Tue | - | Wed | 0.084 | 0.150 | 0.561 | 574 | 0.575 | 1 |
| Tue | - | Thu | -0.346 | 0.147 | -2.355 | 574 | 0.019 | 0.396 |

*[* p < 0.05; ** p < 0.01, *** p < 0.001]*

***Supplementary Table D.17:**  Results of generalized linear model for Z-scored BEG for those with a Normal/Strong SOW of weekday only.

|  |  |  | **95% Confidence Interval** | | |  |  |  |
| --- | --- | --- | --- | --- | --- | --- | --- | --- |
| **Effect** | **Estimate** | **SE** | **Lower** | **Upper** | **β** | **df** | **t** | **p** |
| (Intercept) | -0.005 | 0.061 | -0.124 | 0.115 | 0 | 268 | -0.077 | 0.939 |
| Tue - Mon | -0.018 | 0.219 | -0.449 | 0.413 | -0.018 | 268 | -0.082 | 0.935 |
| Wed - Mon | -0.035 | 0.216 | -0.459 | 0.389 | -0.035 | 268 | -0.162 | 0.871 |
| Thu - Mon | -0.325 | 0.228 | -0.773 | 0.123 | -0.325 | 268 | -1.428 | 0.154 |
| Fri - Mon | -0.189 | 0.229 | -0.64 | 0.263 | -0.189 | 268 | -0.823 | 0.411 |
| Sat - Mon | -0.024 | 0.235 | -0.486 | 0.438 | -0.024 | 268 | -0.102 | 0.919 |
| Sun - Mon | -0.245 | 0.225 | -0.687 | 0.197 | -0.245 | 268 | -1.093 | 0.276 |

*[* p < 0.05; ** p < 0.01, *** p < 0.001]*

***Supplementary Materials Table D.18.:** Results of post-hoc comparisons for Z-scored BEG risk score for those with a Normal/Strong SOW of weekday only.

| **Weekday** |  | **Weekday** | **Difference** | **SE** | **t** | **df** | **p** | **p_bonferroni_** |
| --- | --- | --- | --- | --- | --- | --- | --- | --- |
| Fri | - | Sat | -0.165 | 0.242 | -0.681 | 268 | 0.496 | 1 |
| Fri | - | Sun | 0.057 | 0.232 | 0.244 | 268 | 0.807 | 1 |
| Mon | - | Fri | 0.189 | 0.229 | 0.823 | 268 | 0.411 | 1 |
| Mon | - | Sat | 0.024 | 0.235 | 0.102 | 268 | 0.919 | 1 |
| Mon | - | Sun | 0.245 | 0.225 | 1.093 | 268 | 0.276 | 1 |
| Mon | - | Wed | 0.035 | 0.216 | 0.162 | 268 | 0.871 | 1 |
| Mon | - | Thu | 0.325 | 0.228 | 1.428 | 268 | 0.154 | 1 |
| Mon | - | Tue | 0.018 | 0.219 | 0.082 | 268 | 0.935 | 1 |
| Sat | - | Sun | 0.221 | 0.237 | 0.933 | 268 | 0.352 | 1 |
| Wed | - | Fri | 0.154 | 0.223 | 0.688 | 268 | 0.492 | 1 |
| Wed | - | Sat | -0.011 | 0.229 | -0.048 | 268 | 0.961 | 1 |
| Wed | - | Sun | 0.21 | 0.218 | 0.963 | 268 | 0.337 | 1 |
| Wed | - | Thu | 0.29 | 0.222 | 1.309 | 268 | 0.192 | 1 |
| Thu | - | Fri | -0.136 | 0.235 | -0.58 | 268 | 0.562 | 1 |
| Thu | - | Sat | -0.301 | 0.24 | -1.253 | 268 | 0.211 | 1 |
| Thu | - | Sun | -0.08 | 0.23 | -0.346 | 268 | 0.73 | 1 |
| Tue | - | Fri | 0.171 | 0.227 | 0.753 | 268 | 0.452 | 1 |
| Tue | - | Sat | 0.006 | 0.232 | 0.025 | 268 | 0.98 | 1 |
| Tue | - | Sun | 0.227 | 0.222 | 1.024 | 268 | 0.307 | 1 |
| Tue | - | Wed | 0.017 | 0.213 | 0.08 | 268 | 0.936 | 1 |
| Tue | - | Thu | 0.307 | 0.225 | 1.364 | 268 | 0.174 | 1 |

*[* p < 0.05; ** p < 0.01, *** p < 0.001]*

***Supplementary Table D.19:**  Results of generalized linear model for Z-scored BEG for those with a Normal/Strong SOW of weekday, age, and gender.

|  |  |  | **95% Confidence Interval** | | | |  | |  | |  | |
| --- | --- | --- | --- | --- | --- | --- | --- | --- | --- | --- | --- | --- |
| **Effect** | **Estimate** | **SE** | **Lower** | **Upper** | **β** | **df** | | **t** | | **p** | |  |
| (Intercept) | 0.018 | 0.063 | -0.105 | 0.142 | 0.000 | 266 | | 0.293 | | 0.770 | |  |
| Age | 0.002 | 0.006 | -0.009 | 0.013 | 0.021 | 266 | | 0.341 | | 0.734 | |  |
| Male - Female | 0.193 | 0.127 | -0.058 | 0.444 | 0.193 | 266 | | 1.515 | | 0.131 | |  |
| Tue - Mon | 0.002 | 0.219 | -0.429 | 0.434 | 0.002 | 266 | | 0.011 | | 0.991 | |  |
| Wed - Mon | -0.023 | 0.216 | -0.449 | 0.403 | -0.023 | 266 | | -0.107 | | 0.915 | |  |
| Thu - Mon | -0.295 | 0.229 | -0.745 | 0.155 | -0.295 | 266 | | -1.289 | | 0.198 | |  |
| Fri - Mon | -0.207 | 0.230 | -0.659 | 0.245 | -0.207 | 266 | | -0.901 | | 0.368 | |  |
| Sat - Mon | -0.023 | 0.235 | -0.485 | 0.439 | -0.023 | 266 | | -0.098 | | 0.922 | |  |
| Sun - Mon | -0.249 | 0.224 | -0.691 | 0.193 | -0.249 | 266 | | -1.110 | | 0.268 | |  |

*[* p < 0.05; ** p < 0.01, *** p < 0.001]*

***Supplementary Materials Table D.20.:** Results of post-hoc comparisons for Z-scored BEG risk score for those with a Normal/Strong SOW of weekday, age, and gender.

| **Weekday** |  | **Weekday** | **Difference** | **SE** | **t** | **df** | **p** | **p_bonferroni_** |
| --- | --- | --- | --- | --- | --- | --- | --- | --- |
| Fri | - | Sat | -0.184 | 0.242 | -0.760 | 266 | 0.448 | 1 |
| Fri | - | Sun | 0.042 | 0.232 | 0.181 | 266 | 0.857 | 1 |
| Mon | - | Fri | 0.207 | 0.230 | 0.901 | 266 | 0.368 | 1 |
| Mon | - | Sat | 0.023 | 0.235 | 0.098 | 266 | 0.922 | 1 |
| Mon | - | Sun | 0.249 | 0.224 | 1.110 | 266 | 0.268 | 1 |
| Mon | - | Wed | 0.023 | 0.216 | 0.107 | 266 | 0.915 | 1 |
| Mon | - | Thu | 0.295 | 0.229 | 1.289 | 266 | 0.198 | 1 |
| Mon | - | Tue | -0.002 | 0.219 | -0.011 | 266 | 0.991 | 1 |
| Sat | - | Sun | 0.226 | 0.237 | 0.952 | 266 | 0.342 | 1 |
| Wed | - | Fri | 0.184 | 0.226 | 0.813 | 266 | 0.417 | 1 |
| Wed | - | Sat | 0.000 | 0.230 | 0.000 | 266 | 1 | 1 |
| Wed | - | Sun | 0.226 | 0.219 | 1.030 | 266 | 0.304 | 1 |
| Wed | - | Thu | 0.272 | 0.222 | 1.225 | 266 | 0.222 | 1 |
| Thu | - | Fri | -0.088 | 0.239 | -0.368 | 266 | 0.713 | 1 |
| Thu | - | Sat | -0.272 | 0.242 | -1.123 | 266 | 0.262 | 1 |
| Thu | - | Sun | -0.046 | 0.232 | -0.197 | 266 | 0.844 | 1 |
| Tue | - | Fri | 0.210 | 0.229 | 0.915 | 266 | 0.361 | 1 |
| Tue | - | Sat | 0.026 | 0.233 | 0.110 | 266 | 0.913 | 1 |
| Tue | - | Sun | 0.252 | 0.223 | 1.131 | 266 | 0.259 | 1 |
| Tue | - | Wed | 0.026 | 0.213 | 0.120 | 266 | 0.905 | 1 |
| Tue | - | Thu | 0.297 | 0.225 | 1.321 | 266 | 0.188 | 1 |

*[* p < 0.05; ** p < 0.01, *** p < 0.001]*

***Supplementary Table D.21:**  Results of generalized linear model for Z-scored BEG risk score for those with a Weak SOW of weekday only.

|  |  |  | **95% Confidence Interval** | | |  |  |  |
| --- | --- | --- | --- | --- | --- | --- | --- | --- |
| **Effect** | **Estimate** | **SE** | **Lower** | **Upper** | **β** | **df** | **t** | **p** |
| (Intercept) | -0.003 | 0.042 | -0.085 | 0.078 | 0 | 574 | -0.08 | 0.936 |
| Tue - Mon | -0.193 | 0.158 | -0.504 | 0.117 | -0.193 | 574 | -1.225 | 0.221 |
| Wed - Mon | -0.22 | 0.16 | -0.534 | 0.094 | -0.22 | 574 | -1.374 | 0.17 |
| Thu - Mon | -0.14 | 0.156 | -0.447 | 0.167 | -0.14 | 574 | -0.897 | 0.37 |
| Fri - Mon | -0.22 | 0.155 | -0.524 | 0.085 | -0.22 | 574 | -1.415 | 0.158 |
| Sat - Mon | 0.058 | 0.152 | -0.241 | 0.358 | 0.058 | 574 | 0.382 | 0.702 |
| Sun - Mon | -0.026 | 0.157 | -0.335 | 0.282 | -0.026 | 574 | -0.168 | 0.866 |

*[* p < 0.05; ** p < 0.01, *** p < 0.001]*

***Supplementary Materials Table D.22.:** Results of post-hoc comparisons for Z-scored BEG risk score for those with a Weak SOW of weekday only.

| **Weekday** |  | **Weekday** | **Difference** | **SE** | **t** | **df** | **p** | **p_bonferroni_** |
| --- | --- | --- | --- | --- | --- | --- | --- | --- |
| Fri | - | Sat | -0.278 | 0.15 | -1.858 | 574 | 0.064 | 1 |
| Fri | - | Sun | -0.193 | 0.154 | -1.252 | 574 | 0.211 | 1 |
| Mon | - | Fri | 0.22 | 0.155 | 1.415 | 574 | 0.158 | 1 |
| Mon | - | Sat | -0.058 | 0.152 | -0.382 | 574 | 0.702 | 1 |
| Mon | - | Sun | 0.026 | 0.157 | 0.168 | 574 | 0.866 | 1 |
| Mon | - | Wed | 0.22 | 0.16 | 1.374 | 574 | 0.17 | 1 |
| Mon | - | Thu | 0.14 | 0.156 | 0.897 | 574 | 0.37 | 1 |
| Mon | - | Tue | 0.193 | 0.158 | 1.225 | 574 | 0.221 | 1 |
| Sat | - | Sun | 0.085 | 0.151 | 0.559 | 574 | 0.576 | 1 |
| Wed | - | Fri | -3.789e−4 | 0.157 | -0.002 | 574 | 0.998 | 1 |
| Wed | - | Sat | -0.278 | 0.155 | -1.8 | 574 | 0.072 | 1 |
| Wed | - | Sun | -0.194 | 0.159 | -1.216 | 574 | 0.224 | 1 |
| Wed | - | Thu | -0.08 | 0.158 | -0.505 | 574 | 0.614 | 1 |
| Thu | - | Fri | 0.08 | 0.153 | 0.519 | 574 | 0.604 | 1 |
| Thu | - | Sat | -0.198 | 0.15 | -1.318 | 574 | 0.188 | 1 |
| Thu | - | Sun | -0.114 | 0.155 | -0.732 | 574 | 0.464 | 1 |
| Tue | - | Fri | 0.026 | 0.155 | 0.168 | 574 | 0.867 | 1 |
| Tue | - | Sat | -0.252 | 0.152 | -1.652 | 574 | 0.099 | 1 |
| Tue | - | Sun | -0.167 | 0.157 | -1.064 | 574 | 0.288 | 1 |
| Tue | - | Wed | 0.026 | 0.16 | 0.165 | 574 | 0.869 | 1 |
| Tue | - | Thu | -0.053 | 0.156 | -0.342 | 574 | 0.732 | 1 |

*[* p < 0.05; ** p < 0.01, *** p < 0.001]*

***Supplementary Table D.23:**  Results of generalized linear model for Z-scored BEG risk score for those with a Weak SOW of weekday, age, and gender.

|  |  |  | **95% Confidence Interval** | | |  |  |  |
| --- | --- | --- | --- | --- | --- | --- | --- | --- |
| **Effect** | **Estimate** | **SE** | **Lower** | **Upper** | **β** | **df** | **t** | **p** |
| (Intercept) | 0.053 | 0.045 | -0.036 | 0.142 | 0.000 | 571 | 1.170 | 0.243 |
| Age | 0.002 | 0.003 | -0.005 | 0.008 | 0.019 | 571 | 0.466 | 0.642 |
| Male – Female** | 0.269 | 0.091 | 0.090 | 0.447 | 0.268 | 571 | 2.949 | 0.003 |
| Tue - Mon | -0.174 | 0.157 | -0.483 | 0.135 | -0.174 | 571 | -1.104 | 0.270 |
| Wed - Mon | -0.202 | 0.159 | -0.515 | 0.111 | -0.202 | 571 | -1.266 | 0.206 |
| Thu - Mon | -0.092 | 0.156 | -0.399 | 0.215 | -0.092 | 571 | -0.588 | 0.557 |
| Fri - Mon | -0.219 | 0.155 | -0.522 | 0.085 | -0.219 | 571 | -1.415 | 0.158 |
| Sat - Mon | 0.063 | 0.152 | -0.235 | 0.361 | 0.063 | 571 | 0.414 | 0.679 |
| Sun - Mon | -0.018 | 0.156 | -0.325 | 0.289 | -0.018 | 571 | -0.114 | 0.909 |

*[* p < 0.05; ** p < 0.01, *** p < 0.001]*

***Supplementary Materials Table D.24.:** Results of post-hoc comparisons for Z-scored BEG risk score for those with a Weak SOW of weekday, age, and gender

| **Weekday** |  | **Weekday** | **Difference** | **SE** | **t** | **df** | **p** | **p_bonferroni_** |
| --- | --- | --- | --- | --- | --- | --- | --- | --- |
| Fri | - | Sat | -0.282 | 0.149 | -1.892 | 571 | 0.059 | 1 |
| Fri | - | Sun | -0.201 | 0.153 | -1.310 | 571 | 0.191 | 1 |
| Mon | - | Fri | 0.219 | 0.155 | 1.415 | 571 | 0.158 | 1 |
| Mon | - | Sat | -0.063 | 0.152 | -0.414 | 571 | 0.679 | 1 |
| Mon | - | Sun | 0.018 | 0.156 | 0.114 | 571 | 0.909 | 1 |
| Mon | - | Wed | 0.202 | 0.159 | 1.266 | 571 | 0.206 | 1 |
| Mon | - | Thu | 0.092 | 0.156 | 0.588 | 571 | 0.557 | 1 |
| Mon | - | Tue | 0.174 | 0.157 | 1.104 | 571 | 0.27 | 1 |
| Sat | - | Sun | 0.081 | 0.151 | 0.535 | 571 | 0.593 | 1 |
| Wed | - | Fri | 0.017 | 0.157 | 0.109 | 571 | 0.913 | 1 |
| Wed | - | Sat | -0.265 | 0.154 | -1.721 | 571 | 0.086 | 1 |
| Wed | - | Sun | -0.184 | 0.158 | -1.162 | 571 | 0.246 | 1 |
| Wed | - | Thu | -0.110 | 0.158 | -0.696 | 571 | 0.487 | 1 |
| Thu | - | Fri | 0.127 | 0.154 | 0.826 | 571 | 0.409 | 1 |
| Thu | - | Sat | -0.155 | 0.151 | -1.027 | 571 | 0.305 | 1 |
| Thu | - | Sun | -0.074 | 0.155 | -0.477 | 571 | 0.634 | 1 |
| Tue | - | Fri | 0.045 | 0.155 | 0.292 | 571 | 0.77 | 1 |
| Tue | - | Sat | -0.236 | 0.152 | -1.559 | 571 | 0.12 | 1 |
| Tue | - | Sun | -0.156 | 0.156 | -0.996 | 571 | 0.32 | 1 |
| Tue | - | Wed | 0.028 | 0.159 | 0.177 | 571 | 0.86 | 1 |
| Tue | - | Thu | -0.082 | 0.156 | -0.524 | 571 | 0.6 | 1 |

*[* p < 0.05; ** p < 0.01, *** p < 0.001]*

***Supplementary Table D.25:**  Results of generalized linear model for Z-scored BART for those with a Normal/Strong SOW of weekday only.

|  |  |  | **95% Confidence Interval** | | |  |  |  |
| --- | --- | --- | --- | --- | --- | --- | --- | --- |
| **Effect** | **Estimate** | **SE** | **Lower** | **Upper** | **β** | **df** | **t** | **p** |
| (Intercept) | -0.005 | 0.06 | -0.123 | 0.112 | 0 | 272 | -0.088 | 0.93 |
| Tue - Mon | 0.293 | 0.217 | -0.134 | 0.719 | 0.293 | 272 | 1.351 | 0.178 |
| Wed - Mon | -0.036 | 0.212 | -0.454 | 0.382 | -0.036 | 272 | -0.169 | 0.866 |
| Thu - Mon | 0.247 | 0.225 | -0.196 | 0.69 | 0.247 | 272 | 1.098 | 0.273 |
| Fri - Mon | -0.276 | 0.224 | -0.717 | 0.164 | -0.276 | 272 | -1.236 | 0.217 |
| Sat - Mon | -0.17 | 0.23 | -0.623 | 0.284 | -0.17 | 272 | -0.737 | 0.462 |
| Sun - Mon | -0.007 | 0.222 | -0.444 | 0.43 | -0.007 | 272 | -0.032 | 0.974 |

*[* p < 0.05; ** p < 0.01, *** p < 0.001]*

***Supplementary Materials Table D.26.:** Results of post-hoc comparisons for Z-scored BART risk score for those with a Normal/Strong SOW of weekday only.

| **Weekday** |  | **Weekday** | **Difference** | **SE** | **t** | **df** | **p** | **p_bonferroni_** |
| --- | --- | --- | --- | --- | --- | --- | --- | --- |
| Fri | - | Sat | -0.107 | 0.234 | -0.455 | 272 | 0.65 | 1 |
| Fri | - | Sun | -0.269 | 0.226 | -1.19 | 272 | 0.235 | 1 |
| Mon | - | Fri | 0.276 | 0.224 | 1.236 | 272 | 0.217 | 1 |
| Mon | - | Sat | 0.17 | 0.23 | 0.737 | 272 | 0.462 | 1 |
| Mon | - | Sun | 0.007 | 0.222 | 0.032 | 272 | 0.974 | 1 |
| Mon | - | Wed | 0.036 | 0.212 | 0.169 | 272 | 0.866 | 1 |
| Mon | - | Thu | -0.247 | 0.225 | -1.098 | 272 | 0.273 | 1 |
| Mon | - | Tue | -0.293 | 0.217 | -1.351 | 272 | 0.178 | 1 |
| Sat | - | Sun | -0.163 | 0.233 | -0.698 | 272 | 0.486 | 1 |
| Wed | - | Fri | 0.241 | 0.217 | 1.111 | 272 | 0.268 | 1 |
| Wed | - | Sat | 0.134 | 0.224 | 0.599 | 272 | 0.549 | 1 |
| Wed | - | Sun | -0.029 | 0.215 | -0.134 | 272 | 0.894 | 1 |
| Wed | - | Thu | -0.283 | 0.218 | -1.297 | 272 | 0.196 | 1 |
| Thu | - | Fri | 0.524 | 0.229 | 2.283 | 272 | 0.023 | 0.487 |
| Thu | - | Sat | 0.417 | 0.236 | 1.768 | 272 | 0.078 | 1 |
| Thu | - | Sun | 0.254 | 0.228 | 1.116 | 272 | 0.265 | 1 |
| Tue | - | Fri | 0.569 | 0.221 | 2.574 | 272 | 0.011 | 0.222 |
| Tue | - | Sat | 0.463 | 0.228 | 2.03 | 272 | 0.043 | 0.91 |
| Tue | - | Sun | 0.3 | 0.22 | 1.366 | 272 | 0.173 | 1 |
| Tue | - | Wed | 0.329 | 0.21 | 1.568 | 272 | 0.118 | 1 |
| Tue | - | Thu | 0.046 | 0.223 | 0.205 | 272 | 0.838 | 1 |

*[* p < 0.05; ** p < 0.01, *** p < 0.001]*

***Supplementary Table D.27:**  Results of generalized linear model for Z-scored BART for those with a Normal/Strong SOW of weekday, age, and gender.

|  |  |  | **95% Confidence Interval** | | |  |  |  |
| --- | --- | --- | --- | --- | --- | --- | --- | --- |
| **Effect** | **Estimate** | **SE** | **Lower** | **Upper** | **β** | **df** | **t** | **p** |
| (Intercept) | 0.017 | 0.061 | -0.104 | 0.138 | 0.000 | 270 | 0.277 | 0.782 |
| Male - Female | 0.191 | 0.125 | -0.054 | 0.437 | 0.191 | 270 | 1.536 | 0.126 |
| Age | -0.002 | 0.005 | -0.012 | 0.009 | -0.021 | 270 | -0.352 | 0.725 |
| Tue - Mon | 0.315 | 0.217 | -0.112 | 0.743 | 0.315 | 270 | 1.453 | 0.147 |
| Wed - Mon | -0.016 | 0.213 | -0.435 | 0.402 | -0.016 | 270 | -0.077 | 0.938 |
| Thu - Mon | 0.283 | 0.226 | -0.163 | 0.728 | 0.283 | 270 | 1.250 | 0.212 |
| Fri - Mon | -0.294 | 0.224 | -0.735 | 0.146 | -0.294 | 270 | -1.315 | 0.189 |
| Sat - Mon | -0.177 | 0.230 | -0.630 | 0.276 | -0.177 | 270 | -0.769 | 0.443 |
| Sun - Mon | -0.012 | 0.222 | -0.448 | 0.425 | -0.012 | 270 | -0.052 | 0.959 |

*[* p < 0.05; ** p < 0.01, *** p < 0.001]*

***Supplementary Materials Table D.28.:** Results of post-hoc comparisons for Z-scored BART risk score for those with a Normal/Strong SOW of weekday, age, and gender.

| **Weekday** |  |  | **Difference** | **SE** | **t** | **df** | **p** | **p_bonferroni_** |
| --- | --- | --- | --- | --- | --- | --- | --- | --- |
| Fri | - | Sat | -0.117 | 0.234 | -0.500 | 270 | 0.617 | 1 |
| Fri | - | Sun | -0.283 | 0.226 | -1.249 | 270 | 0.213 | 1 |
| Mon | - | Fri | 0.294 | 0.224 | 1.315 | 270 | 0.189 | 1 |
| Mon | - | Sat | 0.177 | 0.230 | 0.769 | 270 | 0.443 | 1 |
| Mon | - | Sun | 0.012 | 0.222 | 0.052 | 270 | 0.959 | 1 |
| Mon | - | Wed | 0.016 | 0.213 | 0.077 | 270 | 0.938 | 1 |
| Mon | - | Thu | -0.283 | 0.226 | -1.250 | 270 | 0.212 | 1 |
| Mon | - | Tue | -0.315 | 0.217 | -1.453 | 270 | 0.147 | 1 |
| Sat | - | Sun | -0.166 | 0.233 | -0.711 | 270 | 0.478 | 1 |
| Wed | - | Fri | 0.278 | 0.218 | 1.272 | 270 | 0.204 | 1 |
| Wed | - | Sat | 0.161 | 0.225 | 0.714 | 270 | 0.476 | 1 |
| Wed | - | Sun | -0.005 | 0.216 | -0.023 | 270 | 0.982 | 1 |
| Wed | - | Thu | -0.299 | 0.219 | -1.370 | 270 | 0.172 | 1 |
| Thu | - | Fri | 0.577 | 0.232 | 2.489 | 270 | 0.013 | 0.282 |
| Thu | - | Sat | 0.460 | 0.238 | 1.936 | 270 | 0.054 | 1 |
| Thu | - | Sun | 0.294 | 0.229 | 1.284 | 270 | 0.2 | 1 |
| Tue | - | Fri | 0.610 | 0.223 | 2.741 | 270 | 0.007 | 0.137 |
| Tue | - | Sat | 0.493 | 0.229 | 2.154 | 270 | 0.032 | 0.674 |
| Tue | - | Sun | 0.327 | 0.220 | 1.486 | 270 | 0.139 | 1 |
| Tue | - | Wed | 0.332 | 0.210 | 1.583 | 270 | 0.115 | 1 |
| Tue | - | Thu | 0.033 | 0.223 | 0.147 | 270 | 0.884 | 1 |

*[* p < 0.05; ** p < 0.01, *** p < 0.001]*

***Supplementary Table D.29:**  Results of generalized linear model for Z-scored BART for those with a Weak SOW of weekday only.

|  |  |  | **95% Confidence Interval** | | |  |  |  |
| --- | --- | --- | --- | --- | --- | --- | --- | --- |
| **Effect** | **Estimate** | **SE** | **Lower** | **Upper** | **β** | **df** | **t** | **p** |
| (Intercept) | -0.001 | 0.042 | -0.083 | 0.08 | 0 | 577 | -0.033 | 0.974 |
| Tue - Mon | 0.036 | 0.158 | -0.275 | 0.346 | 0.036 | 577 | 0.227 | 0.821 |
| Wed - Mon | -0.035 | 0.16 | -0.349 | 0.28 | -0.035 | 577 | -0.216 | 0.829 |
| Thu - Mon | -0.049 | 0.156 | -0.356 | 0.258 | -0.049 | 577 | -0.315 | 0.753 |
| Fri - Mon | 0.076 | 0.155 | -0.23 | 0.381 | 0.076 | 577 | 0.487 | 0.627 |
| Sat - Mon | 0.053 | 0.152 | -0.246 | 0.352 | 0.053 | 577 | 0.348 | 0.728 |
| Sun - Mon | 0.14 | 0.157 | -0.168 | 0.448 | 0.14 | 577 | 0.893 | 0.372 |

*[* p < 0.05; ** p < 0.01, *** p < 0.001]*

***Supplementary Materials Table D.30.:** Results of post-hoc comparisons for Z-scored BART risk score for those with a Weak SOW of weekday only.

| **Weekday** |  | **Weekday** | **Difference** | **SE** | **t** | **df** | **p** | **p_bonferroni_** |
| --- | --- | --- | --- | --- | --- | --- | --- | --- |
| Fri | - | Sat | 0.023 | 0.15 | 0.152 | 577 | 0.879 | 1 |
| Fri | - | Sun | -0.064 | 0.154 | -0.416 | 577 | 0.677 | 1 |
| Mon | - | Fri | -0.076 | 0.155 | -0.487 | 577 | 0.627 | 1 |
| Mon | - | Sat | -0.053 | 0.152 | -0.348 | 577 | 0.728 | 1 |
| Mon | - | Sun | -0.14 | 0.157 | -0.893 | 577 | 0.372 | 1 |
| Mon | - | Wed | 0.035 | 0.16 | 0.216 | 577 | 0.829 | 1 |
| Mon | - | Thu | 0.049 | 0.156 | 0.315 | 577 | 0.753 | 1 |
| Mon | - | Tue | -0.036 | 0.158 | -0.227 | 577 | 0.821 | 1 |
| Sat | - | Sun | -0.087 | 0.151 | -0.576 | 577 | 0.565 | 1 |
| Wed | - | Fri | -0.11 | 0.158 | -0.698 | 577 | 0.485 | 1 |
| Wed | - | Sat | -0.088 | 0.155 | -0.566 | 577 | 0.572 | 1 |
| Wed | - | Sun | -0.175 | 0.159 | -1.096 | 577 | 0.274 | 1 |
| Wed | - | Thu | 0.015 | 0.159 | 0.091 | 577 | 0.927 | 1 |
| Thu | - | Fri | -0.125 | 0.154 | -0.811 | 577 | 0.418 | 1 |
| Thu | - | Sat | -0.102 | 0.151 | -0.677 | 577 | 0.498 | 1 |
| Thu | - | Sun | -0.189 | 0.155 | -1.218 | 577 | 0.224 | 1 |
| Tue | - | Fri | -0.04 | 0.156 | -0.255 | 577 | 0.799 | 1 |
| Tue | - | Sat | -0.017 | 0.153 | -0.112 | 577 | 0.911 | 1 |
| Tue | - | Sun | -0.104 | 0.157 | -0.662 | 577 | 0.508 | 1 |
| Tue | - | Wed | 0.071 | 0.161 | 0.439 | 577 | 0.661 | 1 |
| Tue | - | Thu | 0.085 | 0.157 | 0.542 | 577 | 0.588 | 1 |

*[* p < 0.05; ** p < 0.01, *** p < 0.001]*

***Supplementary Table D.31:**  Results of generalized linear model for Z-scored BART for those with a Weak SOW of weekday, age, and gender.

|  |  |  | **95% Confidence Interval** | | |  |  |  |
| --- | --- | --- | --- | --- | --- | --- | --- | --- |
| **Effect** | **Estimate** | **SE** | **Lower** | **Upper** | **β** | **df** | **t** | **p** |
| (Intercept) | 0.034 | 0.046 | -0.056 | 0.124 | 0.000 | 574 | 0.747 | 0.455 |
| Male - Female | 0.172 | 0.092 | -0.008 | 0.352 | 0.172 | 574 | 1.878 | 0.061 |
| Age | -0.003 | 0.003 | -0.009 | 0.004 | -0.032 | 574 | -0.774 | 0.439 |
| Tue - Mon | 0.050 | 0.158 | -0.261 | 0.360 | 0.050 | 574 | 0.314 | 0.754 |
| Wed - Mon | -0.027 | 0.160 | -0.341 | 0.288 | -0.027 | 574 | -0.166 | 0.869 |
| Thu - Mon | -0.026 | 0.157 | -0.335 | 0.283 | -0.026 | 574 | -0.166 | 0.868 |
| Fri - Mon | 0.068 | 0.155 | -0.238 | 0.373 | 0.068 | 574 | 0.436 | 0.663 |
| Sat - Mon | 0.055 | 0.152 | -0.243 | 0.354 | 0.055 | 574 | 0.364 | 0.716 |
| Sun - Mon | 0.139 | 0.157 | -0.169 | 0.447 | 0.139 | 574 | 0.888 | 0.375 |

*[* p < 0.05; ** p < 0.01, *** p < 0.001]*

***Supplementary Materials Table D.32.:** Results of post-hoc comparisons for Z-scored BART risk score for those with a Weak SOW of weekday, age, and gender

| **Weekday** |  |  | **Difference** | **SE** | **t** | **df** | **p** | **p_bonferroni_** |
| --- | --- | --- | --- | --- | --- | --- | --- | --- |
| Fri | - | Sat | 0.013 | 0.150 | 0.084 | 574 | 0.933 | 1 |
| Fri | - | Sun | -0.071 | 0.154 | -0.463 | 574 | 0.644 | 1 |
| Mon | - | Fri | -0.068 | 0.156 | -0.436 | 574 | 0.663 | 1 |
| Mon | - | Sat | -0.055 | 0.152 | -0.364 | 574 | 0.716 | 1 |
| Mon | - | Sun | -0.139 | 0.157 | -0.888 | 574 | 0.375 | 1 |
| Mon | - | Wed | 0.027 | 0.160 | 0.166 | 574 | 0.869 | 1 |
| Mon | - | Thu | 0.026 | 0.157 | 0.166 | 574 | 0.868 | 1 |
| Mon | - | Tue | -0.050 | 0.158 | -0.314 | 574 | 0.754 | 1 |
| Sat | - | Sun | -0.084 | 0.151 | -0.555 | 574 | 0.579 | 1 |
| Wed | - | Fri | -0.094 | 0.158 | -0.597 | 574 | 0.551 | 1 |
| Wed | - | Sat | -0.082 | 0.155 | -0.529 | 574 | 0.597 | 1 |
| Wed | - | Sun | -0.166 | 0.159 | -1.041 | 574 | 0.298 | 1 |
| Wed | - | Thu | -3.638e−4 | 0.159 | -0.002 | 574 | 0.998 | 1 |
| Thu | - | Fri | -0.094 | 0.155 | -0.607 | 574 | 0.544 | 1 |
| Thu | - | Sat | -0.081 | 0.151 | -0.538 | 574 | 0.591 | 1 |
| Thu | - | Sun | -0.165 | 0.156 | -1.060 | 574 | 0.29 | 1 |
| Tue | - | Fri | -0.018 | 0.156 | -0.116 | 574 | 0.907 | 1 |
| Tue | - | Sat | -0.006 | 0.153 | -0.037 | 574 | 0.971 | 1 |
| Tue | - | Sun | -0.090 | 0.157 | -0.569 | 574 | 0.569 | 1 |
| Tue | - | Wed | 0.076 | 0.161 | 0.475 | 574 | 0.635 | 1 |
| Tue | - | Thu | 0.076 | 0.157 | 0.482 | 574 | 0.63 | 1 |

*[* p < 0.05; ** p < 0.01, *** p < 0.001]*

**Supplementary Material Figure E:** Comparison of risk score calculated as in main text and calculated in the same manner but without inclusion of the BART score. Error bars represent +/- SE.

**
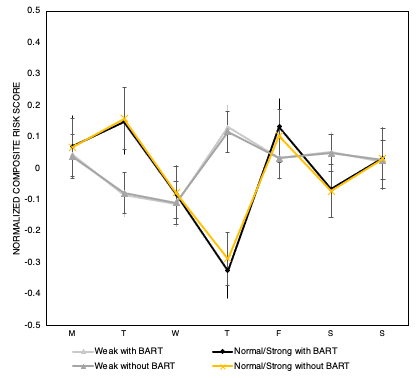
**

***Supplementary Table E.1:**  Results of generalized linear model for composite risk score, without BART, for those with a Normal/Strong SOW of weekday only.

|  |  |  | **95% Confidence Interval** | | |  |  |
| --- | --- | --- | --- | --- | --- | --- | --- |
| **Effect** | **Estimate** | **SE** | **Lower** | **Upper** | **df** | **t** | **p** |
| (Intercept) | 0.01 | 0.036 | -0.061 | 0.081 | 266 | 0.28 | 0.779 |
| Tue - Mon | 0.078 | 0.13 | -0.178 | 0.334 | 266 | 0.598 | 0.55 |
| Wed - Mon | -0.152 | 0.127 | -0.401 | 0.097 | 266 | -1.201 | 0.231 |
| Thu – Mon** | -0.398 | 0.134 | -0.661 | -0.135 | 266 | -2.977 | 0.003 |
| Fri - Mon | 0.06 | 0.135 | -0.205 | 0.325 | 266 | 0.446 | 0.656 |
| Sat - Mon | -0.139 | 0.138 | -0.411 | 0.132 | 266 | -1.01 | 0.313 |
| Sun - Mon | -0.039 | 0.132 | -0.299 | 0.221 | 266 | -0.296 | 0.767 |

*[* p < 0.05; ** p < 0.01, *** p < 0.001]*

***Supplementary Materials Table E.2.:** Results of post-hoc comparisons for composite risk score, without BART, for those with a Normal/Strong SOW of weekday only.

| **Weekday** |  | **Weekday** | **Difference** | **SE** | **t** | **df** | **p** | **p_bonferroni_** |
| --- | --- | --- | --- | --- | --- | --- | --- | --- |
| Fri | - | Sat | 0.199 | 0.142 | 1.403 | 266 | 0.162 | 1 |
| Fri | - | Sun | 0.099 | 0.136 | 0.727 | 266 | 0.468 | 1 |
| Mon | - | Fri | -0.06 | 0.135 | -0.446 | 266 | 0.656 | 1 |
| Mon | - | Sat | 0.139 | 0.138 | 1.01 | 266 | 0.313 | 1 |
| Mon | - | Sun | 0.039 | 0.132 | 0.296 | 266 | 0.767 | 1 |
| Mon | - | Wed | 0.152 | 0.127 | 1.201 | 266 | 0.231 | 1 |
| Mon | - | Thu | 0.398 | 0.134 | 2.977 | 266 | 0.003 | 0.067 |
| Mon | - | Tue | -0.078 | 0.13 | -0.598 | 266 | 0.55 | 1 |
| Sat | - | Sun | -0.1 | 0.139 | -0.719 | 266 | 0.473 | 1 |
| Wed | - | Fri | -0.212 | 0.131 | -1.617 | 266 | 0.107 | 1 |
| Wed | - | Sat | -0.013 | 0.134 | -0.095 | 266 | 0.924 | 1 |
| Wed | - | Sun | -0.113 | 0.128 | -0.881 | 266 | 0.379 | 1 |
| Wed | - | Thu | 0.246 | 0.13 | 1.889 | 266 | 0.06 | 1 |
| Thu | - | Fri* | -0.458 | 0.138 | -3.319 | 266 | 0.001 | 0.022 |
| Thu | - | Sat | -0.259 | 0.141 | -1.833 | 266 | 0.068 | 1 |
| Thu | - | Sun | -0.359 | 0.135 | -2.653 | 266 | 0.008 | 0.178 |
| Tue | - | Fri | 0.018 | 0.135 | 0.132 | 266 | 0.895 | 1 |
| Tue | - | Sat | 0.217 | 0.138 | 1.575 | 266 | 0.116 | 1 |
| Tue | - | Sun | 0.117 | 0.132 | 0.887 | 266 | 0.376 | 1 |
| Tue | - | Wed | 0.23 | 0.127 | 1.816 | 266 | 0.07 | 1 |
| Tue | - | Thu** | 0.476 | 0.134 | 3.56 | 266 | < .001 | 0.009 |

*[* p < 0.05; ** p < 0.01, *** p < 0.001]*

***Supplementary Table E.3:**  Results of generalized linear model for composite risk score, without BART, for those with a Normal/Strong SOW of weekday, age, and gender.

|  |  |  | **95% Confidence Interval** | | | |  | |  | |
| --- | --- | --- | --- | --- | --- | --- | --- | --- | --- | --- |
| **Effect** | **Estimate** | **SE** | **Lower** | **Upper** | **df** | **t** | | **p** | |  |
| (Intercept) | 0.035 | 0.036 | -0.036 | 0.106 | 264 | 0.970 | | 0.333 | |  |
| Male – Female** | 0.223 | 0.073 | 0.079 | 0.367 | 264 | 3.048 | | 0.003 | |  |
| Age* | -0.008 | 0.003 | -0.014 | -0.002 | 264 | -2.550 | | 0.011 | |  |
| Tue - Mon | 0.107 | 0.127 | -0.144 | 0.358 | 264 | 0.837 | | 0.403 | |  |
| Wed - Mon | -0.110 | 0.124 | -0.355 | 0.135 | 264 | -0.885 | | 0.377 | |  |
| Thu – Mon** | -0.347 | 0.131 | -0.606 | -0.089 | 264 | -2.645 | | 0.009 | |  |
| Fri - Mon | 0.020 | 0.132 | -0.240 | 0.280 | 264 | 0.151 | | 0.880 | |  |
| Sat - Mon | -0.153 | 0.135 | -0.418 | 0.113 | 264 | -1.133 | | 0.258 | |  |
| Sun - Mon | -0.045 | 0.129 | -0.299 | 0.208 | 264 | -0.351 | | 0.726 | |  |

*[* p < 0.05; ** p < 0.01, *** p < 0.001]*

***Supplementary Materials Table E.4:** Results of post-hoc comparisons for composite risk score, without BART, for those with a Normal/Strong SOW of weekday, age, and gender.

| **Weekday** |  |  | **Difference** | **SE** | **t** | **df** | **p** | **p_bonferroni_** |
| --- | --- | --- | --- | --- | --- | --- | --- | --- |
| Fri | - | Sat | 0.173 | 0.139 | 1.242 | 264 | 0.215 | 1 |
| Fri | - | Sun | 0.065 | 0.133 | 0.488 | 264 | 0.626 | 1 |
| Mon | - | Fri | -0.020 | 0.132 | -0.151 | 264 | 0.88 | 1 |
| Mon | - | Sat | 0.153 | 0.135 | 1.133 | 264 | 0.258 | 1 |
| Mon | - | Sun | 0.045 | 0.129 | 0.351 | 264 | 0.726 | 1 |
| Mon | - | Wed | 0.110 | 0.124 | 0.885 | 264 | 0.377 | 1 |
| Mon | - | Thu | 0.347 | 0.131 | 2.645 | 264 | 0.009 | 0.182 |
| Mon | - | Tue | -0.107 | 0.128 | -0.837 | 264 | 0.403 | 1 |
| Sat | - | Sun | -0.108 | 0.136 | -0.789 | 264 | 0.431 | 1 |
| Wed | - | Fri | -0.130 | 0.130 | -0.999 | 264 | 0.319 | 1 |
| Wed | - | Sat | 0.043 | 0.132 | 0.324 | 264 | 0.746 | 1 |
| Wed | - | Sun | -0.065 | 0.126 | -0.513 | 264 | 0.608 | 1 |
| Wed | - | Thu | 0.238 | 0.127 | 1.864 | 264 | 0.063 | 1 |
| Thu | - | Fri | -0.367 | 0.137 | -2.681 | 264 | 0.008 | 0.164 |
| Thu | - | Sat | -0.195 | 0.139 | -1.401 | 264 | 0.162 | 1 |
| Thu | - | Sun | -0.302 | 0.133 | -2.270 | 264 | 0.024 | 0.504 |
| Tue | - | Fri | 0.087 | 0.133 | 0.654 | 264 | 0.514 | 1 |
| Tue | - | Sat | 0.259 | 0.135 | 1.920 | 264 | 0.056 | 1 |
| Tue | - | Sun | 0.152 | 0.129 | 1.176 | 264 | 0.241 | 1 |
| Tue | - | Wed | 0.217 | 0.124 | 1.749 | 264 | 0.081 | 1 |
| Tue | - | Thu* | 0.454 | 0.131 | 3.474 | 264 | < .001 | 0.013 |

*[* p < 0.05; ** p < 0.01, *** p < 0.001]*

***Supplementary Table E.5:**  Results of generalized linear model for composite risk score, without BART, for those with a Weak SOW of weekday only.

|  |  |  | **95% Confidence Interval** | | |  |  |
| --- | --- | --- | --- | --- | --- | --- | --- |
| **Effect** | **Estimate** | **SE** | **Lower** | **Upper** | **df** | **t** | **p** |
| (Intercept) | -6.345e−4 | 0.026 | -0.051 | 0.05 | 574 | -0.025 | 0.98 |
| Tue - Mon | -0.128 | 0.098 | -0.321 | 0.066 | 574 | -1.298 | 0.195 |
| Wed - Mon | -0.157 | 0.1 | -0.353 | 0.039 | 574 | -1.57 | 0.117 |
| Thu - Mon | 0.087 | 0.097 | -0.104 | 0.278 | 574 | 0.896 | 0.371 |
| Fri - Mon | -0.012 | 0.097 | -0.202 | 0.178 | 574 | -0.128 | 0.898 |
| Sat - Mon | 0.007 | 0.095 | -0.179 | 0.194 | 574 | 0.078 | 0.938 |
| Sun - Mon | -0.024 | 0.098 | -0.216 | 0.169 | 574 | -0.24 | 0.81 |

*[* p < 0.05; ** p < 0.01, *** p < 0.001]*

***Supplementary Materials Table E.6:** Results of post-hoc comparisons for composite risk score, without BART, for those with a Weak SOW of weekday only.

| **Weekday** |  | **Weekday** | **Difference** | **SE** | **t** | **df** | **p** | **p_bonferroni_** |
| --- | --- | --- | --- | --- | --- | --- | --- | --- |
| Fri | - | Sat | -0.02 | 0.093 | -0.212 | 574 | 0.832 | 1 |
| Fri | - | Sun | 0.011 | 0.096 | 0.116 | 574 | 0.908 | 1 |
| Mon | - | Fri | 0.012 | 0.097 | 0.128 | 574 | 0.898 | 1 |
| Mon | - | Sat | -0.007 | 0.095 | -0.078 | 574 | 0.938 | 1 |
| Mon | - | Sun | 0.024 | 0.098 | 0.24 | 574 | 0.81 | 1 |
| Mon | - | Wed | 0.157 | 0.1 | 1.57 | 574 | 0.117 | 1 |
| Mon | - | Thu | -0.087 | 0.097 | -0.896 | 574 | 0.371 | 1 |
| Mon | - | Tue | 0.128 | 0.098 | 1.298 | 574 | 0.195 | 1 |
| Sat | - | Sun | 0.031 | 0.094 | 0.328 | 574 | 0.743 | 1 |
| Wed | - | Fri | -0.144 | 0.098 | -1.472 | 574 | 0.142 | 1 |
| Wed | - | Sat | -0.164 | 0.096 | -1.704 | 574 | 0.089 | 1 |
| Wed | - | Sun | -0.133 | 0.099 | -1.343 | 574 | 0.18 | 1 |
| Wed | - | Thu | -0.244 | 0.099 | -2.473 | 574 | 0.014 | 0.287 |
| Thu | - | Fri | 0.1 | 0.096 | 1.042 | 574 | 0.298 | 1 |
| Thu | - | Sat | 0.08 | 0.094 | 0.851 | 574 | 0.395 | 1 |
| Thu | - | Sun | 0.111 | 0.097 | 1.145 | 574 | 0.253 | 1 |
| Tue | - | Fri | -0.115 | 0.097 | -1.193 | 574 | 0.233 | 1 |
| Tue | - | Sat | -0.135 | 0.095 | -1.424 | 574 | 0.155 | 1 |
| Tue | - | Sun | -0.104 | 0.098 | -1.066 | 574 | 0.287 | 1 |
| Tue | - | Wed | 0.029 | 0.1 | 0.289 | 574 | 0.773 | 1 |
| Tue | - | Thu | -0.215 | 0.097 | -2.21 | 574 | 0.028 | 0.578 |

*[* p < 0.05; ** p < 0.01, *** p < 0.001]*

***Supplementary Table E.7:** Results of generalized linear model for composite risk score, without BART, for those with a Weak SOW of weekday, age, and gender.

|  |  |  | **95% Confidence Interval** | | |  | |  |
| --- | --- | --- | --- | --- | --- | --- | --- | --- |
| **Effect** | **Estimate** | **SE** | **Lower** | **Upper** | **df** | | **t** | **p** |
| (Intercept) | 0.081 | 0.026 | 0.029 | 0.133 | 571 | | 3.072 | 0.002 |
| Male – Female*** | 0.395 | 0.053 | 0.291 | 0.500 | 571 | | 7.443 | < .001 |
| Age*** | -0.012 | 0.002 | -0.015 | -0.008 | 571 | | -5.999 | < .001 |
| Tue - Mon | -0.096 | 0.092 | -0.276 | 0.084 | 571 | | -1.050 | 0.294 |
| Wed - Mon | -0.146 | 0.093 | -0.328 | 0.037 | 571 | | -1.568 | 0.117 |
| Thu - Mon | 0.135 | 0.091 | -0.044 | 0.314 | 571 | | 1.479 | 0.140 |
| Fri - Mon | -0.045 | 0.090 | -0.223 | 0.132 | 571 | | -0.505 | 0.614 |
| Sat - Mon | 0.009 | 0.088 | -0.164 | 0.183 | 571 | | 0.104 | 0.917 |
| Sun - Mon | -0.039 | 0.091 | -0.218 | 0.140 | 571 | | -0.424 | 0.672 |

*[* p < 0.05; ** p < 0.01, *** p < 0.001]*

***Supplementary Materials Table E.8.:** Results of post-hoc comparisons for composite risk score, without BART, for those with a Weak SOW of weekday, age, and gender.

| **Weekday** |  |  | **Difference** | **SE** | **t** | **df** | **p** | **p_bonferroni_** |
| --- | --- | --- | --- | --- | --- | --- | --- | --- |
| Fri | - | Sat | -0.055 | 0.087 | -0.630 | 571 | 0.529 | 1 |
| Fri | - | Sun | -0.007 | 0.089 | -0.077 | 571 | 0.939 | 1 |
| Mon | - | Fri | 0.045 | 0.090 | 0.505 | 571 | 0.614 | 1 |
| Mon | - | Sat | -0.009 | 0.088 | -0.104 | 571 | 0.917 | 1 |
| Mon | - | Sun | 0.039 | 0.091 | 0.424 | 571 | 0.672 | 1 |
| Mon | - | Wed | 0.146 | 0.093 | 1.568 | 571 | 0.117 | 1 |
| Mon | - | Thu | -0.135 | 0.091 | -1.479 | 571 | 0.14 | 1 |
| Mon | - | Tue | 0.096 | 0.092 | 1.050 | 571 | 0.294 | 1 |
| Sat | - | Sun | 0.048 | 0.088 | 0.545 | 571 | 0.586 | 1 |
| Wed | - | Fri | -0.100 | 0.091 | -1.097 | 571 | 0.273 | 1 |
| Wed | - | Sat | -0.155 | 0.090 | -1.728 | 571 | 0.085 | 1 |
| Wed | - | Sun | -0.107 | 0.092 | -1.159 | 571 | 0.247 | 1 |
| Wed | - | Thu | -0.281 | 0.092 | -3.045 | 571 | 0.002 | 0.051 |
| Thu | - | Fri | 0.180 | 0.090 | 2.013 | 571 | 0.045 | 0.937 |
| Thu | - | Sat | 0.126 | 0.088 | 1.431 | 571 | 0.153 | 1 |
| Thu | - | Sun | 0.174 | 0.091 | 1.917 | 571 | 0.056 | 1 |
| Tue | - | Fri | -0.051 | 0.090 | -0.563 | 571 | 0.574 | 1 |
| Tue | - | Sat | -0.106 | 0.088 | -1.193 | 571 | 0.233 | 1 |
| Tue | - | Sun | -0.058 | 0.091 | -0.632 | 571 | 0.528 | 1 |
| Tue | - | Wed | 0.049 | 0.093 | 0.532 | 571 | 0.595 | 1 |
| Tue | - | Thu | -0.231 | 0.091 | -2.543 | 571 | 0.011 | 0.237 |

*[* p < 0.05; ** p < 0.01, *** p < 0.001]*

**Study 2:**

##

**Supplementary Table F:** Chi-square test was used to determine any deviations in observed frequencies of males in strong [χ2(6, N = 1119) = 5.65, p = 0.46)] and weak [χ2(6, N = 114) = 6.47, p = 0.37)] groups. A t-test was used to determine whether there were significant variations in ages between the strong/normal and weak groups and was found to be significant [t(12) = 2.39, p= 0.03]. There were not significantly more males in the Normal/Strong SOW group than in the Weak SOW group [t(819.1) = -0.972, p = 0.332.

| **Sense of weekday** | **Day of the Week** | **N** | **% Male** | **Average Age (***σ*_M_**)** |
| --- | --- | --- | --- | --- |
| **Strong, Normal** | *Monday* | 64 | 31.25 | 35.67 (1.43) |
|  | *Tuesday* | 56 | 33.93 | 31.27 (1.30) |
|  | *Wednesday* | 56 | 35.71 | 33.14 (1.53) |
|  | *Thursday* | 59 | 22.03 | 34.27 (1.72) |
|  | *Friday* | 50 | 20.00 | 35.64 (1.69) |
|  | *Saturday* | 62 | 32.26 | 32.69 (1.39) |
|  | *Sunday* | 54 | 31.48 | 33.15 (1.50) |
| **Weak** | *Monday* | 54 | 27.78 | 31.43 (1.77) |
|  | *Tuesday* | 61 | 18.03 | 33.84 (1.59) |
|  | *Wednesday* | 64 | 29.69 | 30.34 (1.19) |
|  | *Thursday* | 58 | 24.14 | 32.40 (1.53) |
|  | *Friday* | 68 | 27.94 | 32.26 (1.39) |
|  | *Saturday* | 56 | 23.21 | 32.34 (1.55) |
|  | *Sunday* | 67 | 34.33 | 30.39 (1.52) |

***Supplementary Table G.1:**  Results of generalized linear model for Z-scored composite risk score for those with a Normal/Strong SOW of weekday only.

|  |  |  | **95% Confidence Interval** | | |  |  |  |
| --- | --- | --- | --- | --- | --- | --- | --- | --- |
| **Effect** | **Estimate** | **SE** | **Lower** | **Upper** | **β** | **df** | **t** | **p** |
| (Intercept) | -0.025 | 0.027 | -0.077 | 0.028 | 0 | 394 | -0.919 | 0.359 |
| Tue - Mon | 0.014 | 0.098 | -0.178 | 0.206 | 0.026 | 394 | 0.141 | 0.888 |
| Wed - Mon | 0.011 | 0.098 | -0.181 | 0.203 | 0.021 | 394 | 0.117 | 0.907 |
| Thu - Mon | 0.092 | 0.096 | -0.097 | 0.282 | 0.173 | 394 | 0.958 | 0.339 |
| Fri - Mon | -0.056 | 0.101 | -0.254 | 0.142 | -0.105 | 394 | -0.558 | 0.577 |
| Sat - Mon | -0.12 | 0.095 | -0.307 | 0.067 | -0.225 | 394 | -1.264 | 0.207 |
| Sun - Mon | -0.083 | 0.099 | -0.276 | 0.111 | -0.155 | 394 | -0.837 | 0.403 |

*[* p < 0.05; ** p < 0.01, *** p < 0.001]*

***Supplementary Materials Table G.2.:** Results of post-hoc comparisons for Z-scored composite risk score for those with a Normal/Strong SOW of weekday only.

| **Weekday** |  | **Weekday** | **Difference** | **SE** | **t** | **df** | **p** | **p_bonferroni_** |
| --- | --- | --- | --- | --- | --- | --- | --- | --- |
| Fri | - | Sat | 0.064 | 0.101 | 0.631 | 394 | 0.529 | 1 |
| Fri | - | Sun | 0.026 | 0.105 | 0.251 | 394 | 0.802 | 1 |
| Mon | - | Fri | 0.056 | 0.101 | 0.558 | 394 | 0.577 | 1 |
| Mon | - | Sat | 0.12 | 0.095 | 1.264 | 394 | 0.207 | 1 |
| Mon | - | Sun | 0.083 | 0.099 | 0.837 | 394 | 0.403 | 1 |
| Mon | - | Wed | -0.011 | 0.098 | -0.117 | 394 | 0.907 | 1 |
| Mon | - | Thu | -0.092 | 0.096 | -0.958 | 394 | 0.339 | 1 |
| Mon | - | Tue | -0.014 | 0.098 | -0.141 | 394 | 0.888 | 1 |
| Sat | - | Sun | -0.038 | 0.099 | -0.379 | 394 | 0.705 | 1 |
| Wed | - | Fri | 0.068 | 0.104 | 0.652 | 394 | 0.515 | 1 |
| Wed | - | Sat | 0.132 | 0.098 | 1.338 | 394 | 0.182 | 1 |
| Wed | - | Sun | 0.094 | 0.102 | 0.923 | 394 | 0.357 | 1 |
| Wed | - | Thu | -0.081 | 0.1 | -0.812 | 394 | 0.418 | 1 |
| Thu | - | Fri | 0.148 | 0.103 | 1.447 | 394 | 0.149 | 1 |
| Thu | - | Sat | 0.212 | 0.097 | 2.189 | 394 | 0.029 | 0.613 |
| Thu | - | Sun | 0.175 | 0.1 | 1.739 | 394 | 0.083 | 1 |
| Tue | - | Fri | 0.07 | 0.104 | 0.674 | 394 | 0.501 | 1 |
| Tue | - | Sat | 0.134 | 0.098 | 1.362 | 394 | 0.174 | 1 |
| Tue | - | Sun | 0.096 | 0.102 | 0.946 | 394 | 0.345 | 1 |
| Tue | - | Wed | 0.002 | 0.101 | 0.023 | 394 | 0.982 | 1 |
| Tue | - | Thu | -0.078 | 0.1 | -0.788 | 394 | 0.431 | 1 |

*[* p < 0.05; ** p < 0.01, *** p < 0.001]*

***Supplementary Table G.3:**  Results of generalized linear model for Z-scored composite risk score for those with a Normal/Strong SOW of weekday, age, and gender.

|  |  |  | **95% Confidence Interval** | | |  |  |  |
| --- | --- | --- | --- | --- | --- | --- | --- | --- |
| **Effect** | **Estimate** | **SE** | **Lower** | **Upper** | **β** | **df** | **t** | **p** |
| (Intercept) | 0.043 | 0.028 | -0.012 | 0.097 | 0.000 | 392 | 1.549 | 0.122 |
| Age *** | -0.009 | 0.002 | -0.013 | -0.004 | -0.189 | 392 | -3.984 | < .001 |
| Male – Female *** | 0.328 | 0.055 | 0.220 | 0.437 | 0.615 | 392 | 5.939 | < .001 |
| Tue - Mon | -0.034 | 0.092 | -0.215 | 0.148 | -0.063 | 392 | -0.367 | 0.714 |
| Wed - Mon | -0.026 | 0.092 | -0.206 | 0.155 | -0.048 | 392 | -0.277 | 0.782 |
| Thu - Mon | 0.110 | 0.091 | -0.068 | 0.289 | 0.206 | 392 | 1.214 | 0.226 |
| Fri - Mon | -0.020 | 0.095 | -0.206 | 0.167 | -0.037 | 392 | -0.206 | 0.837 |
| Sat - Mon | -0.150 | 0.090 | -0.326 | 0.027 | -0.281 | 392 | -1.670 | 0.096 |
| Sun - Mon | -0.106 | 0.093 | -0.288 | 0.077 | -0.198 | 392 | -1.136 | 0.257 |

*[* p < 0.05; ** p < 0.01, *** p < 0.001]*

***Supplementary Materials Table G.4:** Results of post-hoc comparisons for Z-scored composite risk score for those with a Normal/Strong SOW of weekday, age, and gender.

| **Weekday** | | | **Difference** | **SE** | **t** | **df** | **p** | **p_bonferroni_** |
| --- | --- | --- | --- | --- | --- | --- | --- | --- |
| Fri | - | Sat | 0.130 | 0.096 | 1.359 | 392 | 0.175 | 1 |
| Fri | - | Sun | 0.086 | 0.099 | 0.870 | 392 | 0.385 | 1 |
| Mon | - | Fri | 0.020 | 0.095 | 0.206 | 392 | 0.837 | 1 |
| Mon | - | Sat | 0.150 | 0.090 | 1.670 | 392 | 0.096 | 1 |
| Mon | - | Sun | 0.106 | 0.093 | 1.136 | 392 | 0.257 | 1 |
| Mon | - | Wed | 0.026 | 0.092 | 0.277 | 392 | 0.782 | 1 |
| Mon | - | Thu | -0.110 | 0.091 | -1.214 | 392 | 0.226 | 1 |
| Mon | - | Tue | 0.034 | 0.092 | 0.367 | 392 | 0.714 | 1 |
| Sat | - | Sun | -0.044 | 0.093 | -0.474 | 392 | 0.636 | 1 |
| Wed | - | Fri | -0.006 | 0.098 | -0.061 | 392 | 0.952 | 1 |
| Wed | - | Sat | 0.124 | 0.093 | 1.343 | 392 | 0.18 | 1 |
| Wed | - | Sun | 0.080 | 0.096 | 0.836 | 392 | 0.404 | 1 |
| Wed | - | Thu | -0.136 | 0.094 | -1.444 | 392 | 0.149 | 1 |
| Thu | - | Fri | 0.130 | 0.097 | 1.344 | 392 | 0.18 | 1 |
| Thu | - | Sat | 0.260 | 0.091 | 2.841 | 392 | 0.005 | 0.099 |
| Thu | - | Sun | 0.216 | 0.095 | 2.278 | 392 | 0.023 | 0.488 |
| Tue | - | Fri | -0.014 | 0.098 | -0.145 | 392 | 0.884 | 1 |
| Tue | - | Sat | 0.116 | 0.093 | 1.252 | 392 | 0.211 | 1 |
| Tue | - | Sun | 0.072 | 0.096 | 0.748 | 392 | 0.455 | 1 |
| Tue | - | Wed | -0.008 | 0.095 | -0.088 | 392 | 0.93 | 1 |
| Tue | - | Thu | -0.144 | 0.094 | -1.531 | 392 | 0.126 | 1 |

*[* p < 0.05; ** p < 0.01, *** p < 0.001]*

***Supplementary Table G.5:**  Results of generalized linear model for Z-scored composite risk score for those with a Weak SOW of weekday only.

|  |  |  | **95% Confidence Interval** | | |  |  |  |
| --- | --- | --- | --- | --- | --- | --- | --- | --- |
| **Effect** | **Estimate** | **SE** | **Lower** | **Upper** | **β** | **df** | **t** | **p** |
| (Intercept) | 0.023 | 0.027 | -0.031 | 0.077 | 0 | 421 | 0.847 | 0.398 |
| Tue - Mon | 0.021 | 0.105 | -0.186 | 0.228 | 0.037 | 421 | 0.198 | 0.843 |
| Wed - Mon | 0.114 | 0.104 | -0.09 | 0.319 | 0.203 | 421 | 1.096 | 0.274 |
| Thu - Mon | 0.131 | 0.106 | -0.078 | 0.34 | 0.233 | 421 | 1.231 | 0.219 |
| Fri - Mon | 0.018 | 0.103 | -0.184 | 0.22 | 0.032 | 421 | 0.175 | 0.861 |
| Sat - Mon | 0.122 | 0.107 | -0.089 | 0.333 | 0.217 | 421 | 1.132 | 0.258 |
| Sun - Mon | 0.027 | 0.103 | -0.175 | 0.23 | 0.049 | 421 | 0.267 | 0.79 |

*[* p < 0.05; ** p < 0.01, *** p < 0.001]*

***Supplementary Materials Table G.6:** Results of post-hoc comparisons for Z-scored composite risk score for those with a Weak SOW of weekday only.

| **Weekday** |  | **Weekday** | **Difference** | **SE** | **t** | **df** | **p** | **p_bonferroni_** |
| --- | --- | --- | --- | --- | --- | --- | --- | --- |
| Fri | - | Sat | -0.104 | 0.102 | -1.02 | 421 | 0.308 | 1 |
| Fri | - | Sun | -0.009 | 0.097 | -0.098 | 421 | 0.922 | 1 |
| Mon | - | Fri | -0.018 | 0.103 | -0.175 | 421 | 0.861 | 1 |
| Mon | - | Sat | -0.122 | 0.107 | -1.132 | 421 | 0.258 | 1 |
| Mon | - | Sun | -0.027 | 0.103 | -0.267 | 421 | 0.79 | 1 |
| Mon | - | Wed | -0.114 | 0.104 | -1.096 | 421 | 0.274 | 1 |
| Mon | - | Thu | -0.131 | 0.106 | -1.231 | 421 | 0.219 | 1 |
| Mon | - | Tue | -0.021 | 0.105 | -0.198 | 421 | 0.843 | 1 |
| Sat | - | Sun | 0.094 | 0.102 | 0.924 | 421 | 0.356 | 1 |
| Wed | - | Fri | 0.096 | 0.098 | 0.979 | 421 | 0.328 | 1 |
| Wed | - | Sat | -0.008 | 0.103 | -0.074 | 421 | 0.941 | 1 |
| Wed | - | Sun | 0.087 | 0.098 | 0.88 | 421 | 0.38 | 1 |
| Wed | - | Thu | -0.017 | 0.102 | -0.167 | 421 | 0.868 | 1 |
| Thu | - | Fri | 0.113 | 0.101 | 1.123 | 421 | 0.262 | 1 |
| Thu | - | Sat | 0.009 | 0.106 | 0.089 | 421 | 0.929 | 1 |
| Thu | - | Sun | 0.104 | 0.101 | 1.026 | 421 | 0.306 | 1 |
| Tue | - | Fri | 0.003 | 0.099 | 0.029 | 421 | 0.977 | 1 |
| Tue | - | Sat | -0.101 | 0.104 | -0.967 | 421 | 0.334 | 1 |
| Tue | - | Sun | -0.007 | 0.1 | -0.066 | 421 | 0.947 | 1 |
| Tue | - | Wed | -0.093 | 0.101 | -0.925 | 421 | 0.356 | 1 |
| Tue | - | Thu | -0.11 | 0.103 | -1.067 | 421 | 0.287 | 1 |

*[* p < 0.05; ** p < 0.01, *** p < 0.001]*

***Supplementary Table G.7:** Results of generalized linear model for Z-scored composite risk score for those with a Weak SOW of weekday, age, and gender.

|  |  |  | **95% Confidence Interval** | | |  | | |  | |  |  |
| --- | --- | --- | --- | --- | --- | --- | --- | --- | --- | --- | --- | --- |
| **Effect** | **Estimate** | **SE** | **Lower** | **Upper** | **β** | | **df** | **t** | | **p** | |  |
| (Intercept) | 0.092 | 0.030 | 0.034 | 0.151 | 0.000 | | 419 | 3.113 | | 0.002 | |  |
| Age *** | -0.008 | 0.002 | -0.013 | -0.004 | -0.176 | | 419 | -3.762 | | < .001 | |  |
| Male - Female *** | 0.293 | 0.059 | 0.176 | 0.410 | 0.522 | | 419 | 4.936 | | < .001 | |  |
| Tue - Mon | 0.070 | 0.101 | -0.129 | 0.269 | 0.124 | | 419 | 0.690 | | 0.491 | |  |
| Wed - Mon | 0.099 | 0.100 | -0.097 | 0.296 | 0.177 | | 419 | 0.995 | | 0.320 | |  |
| Thu - Mon | 0.150 | 0.102 | -0.051 | 0.351 | 0.267 | | 419 | 1.468 | | 0.143 | |  |
| Fri - Mon | 0.025 | 0.098 | -0.169 | 0.218 | 0.044 | | 419 | 0.250 | | 0.803 | |  |
| Sat - Mon | 0.143 | 0.103 | -0.060 | 0.345 | 0.254 | | 419 | 1.385 | | 0.167 | |  |
| Sun - Mon | -5.191e−4 | 0.099 | -0.195 | 0.194 | -9.243e−4 | | 419 | -0.005 | | 0.996 | |  |

*[* p < 0.05; ** p < 0.01, *** p < 0.001]*

***Supplementary Materials Table G.8.:** Results of post-hoc comparisons for Z-scored composite risk score for those with a Weak SOW of weekday, age, and gender.

| **DOW** |  |  | **Difference** | **SE** | **t** | **df** | **p** | **p_bonferroni_** |
| --- | --- | --- | --- | --- | --- | --- | --- | --- |
| Fri | - | Sat | -0.118 | 0.097 | -1.212 | 419 | 0.226 | 1 |
| Fri | - | Sun | 0.025 | 0.093 | 0.270 | 419 | 0.788 | 1 |
| Mon | - | Fri | -0.025 | 0.098 | -0.250 | 419 | 0.803 | 1 |
| Mon | - | Sat | -0.143 | 0.103 | -1.385 | 419 | 0.167 | 1 |
| Mon | - | Sun | 0.001 | 0.099 | 0.005 | 419 | 0.996 | 1 |
| Mon | - | Wed | -0.099 | 0.100 | -0.995 | 419 | 0.32 | 1 |
| Mon | - | Thu | -0.150 | 0.102 | -1.468 | 419 | 0.143 | 1 |
| Mon | - | Tue | -0.070 | 0.101 | -0.690 | 419 | 0.491 | 1 |
| Sat | - | Sun | 0.143 | 0.098 | 1.460 | 419 | 0.145 | 1 |
| Wed | - | Fri | 0.075 | 0.094 | 0.794 | 419 | 0.428 | 1 |
| Wed | - | Sat | -0.043 | 0.099 | -0.439 | 419 | 0.661 | 1 |
| Wed | - | Sun | 0.100 | 0.094 | 1.057 | 419 | 0.291 | 1 |
| Wed | - | Thu | -0.051 | 0.098 | -0.516 | 419 | 0.606 | 1 |
| Thu | - | Fri | 0.125 | 0.097 | 1.298 | 419 | 0.195 | 1 |
| Thu | - | Sat | 0.007 | 0.101 | 0.071 | 419 | 0.943 | 1 |
| Thu | - | Sun | 0.150 | 0.097 | 1.549 | 419 | 0.122 | 1 |
| Tue | - | Fri | 0.045 | 0.095 | 0.473 | 419 | 0.636 | 1 |
| Tue | - | Sat | -0.073 | 0.100 | -0.729 | 419 | 0.466 | 1 |
| Tue | - | Sun | 0.070 | 0.096 | 0.730 | 419 | 0.466 | 1 |
| Tue | - | Wed | -0.030 | 0.097 | -0.304 | 419 | 0.761 | 1 |
| Tue | - | Thu | -0.080 | 0.099 | -0.808 | 419 | 0.419 | 1 |

*[* p < 0.05; ** p < 0.01, *** p < 0.001]*

**Supplementary Table H:** Individual risk measurement descriptives by weekday and Sense of Week (SOW) in Study 2.

|  |  |  | **N** | **Mean** | ***σ*_M_** |
| --- | --- | --- | --- | --- | --- |
| **SOEP** | **Strong, Normal** | *Monday* | 64 | 0.07421 | 0.1177 |
|  |  | *Tuesday* | 56 | -0.03577 | 0.1402 |
|  |  | *Wednesday* | 56 | -0.03577 | 0.1211 |
|  |  | *Thursday* | 59 | 0.2507 | 0.1262 |
|  |  | *Friday* | 50 | -0.08084 | 0.1477 |
|  |  | *Saturday* | 62 | -0.07610 | 0.1243 |
|  |  | *Sunday* | 54 | 0.08371 | 0.1225 |
|  | **Weak** | *Monday* | 54 | 0.01168 | 0.1519 |
|  |  | *Tuesday* | 61 | -0.05576 | 0.1271 |
|  |  | *Wednesday* | 64 | 0.04720 | 0.1266 |
|  |  | *Thursday* | 58 | -0.08739 | 0.1344 |
|  |  | *Friday* | 68 | -0.09024 | 0.1266 |
|  |  | *Saturday* | 56 | 0.1572 | 0.1343 |
|  |  | *Sunday* | 67 | -0.1364 | 0.1292 |
| **DOSPERT** | **Strong, Normal** | *Monday* | 64 | -0.04650 | 0.08794 |
|  |  | *Tuesday* | 56 | -0.003154 | 0.08736 |
|  |  | *Wednesday* | 56 | -0.01669 | 0.08081 |
|  |  | *Thursday* | 59 | 0.09547 | 0.1018 |
|  |  | *Friday* | 50 | -0.1398 | 0.07726 |
|  |  | *Saturday* | 62 | -0.1684 | 0.07874 |
|  |  | *Sunday* | 54 | -0.1328 | 0.0742 |
|  | **Weak** | *Monday* | 54 | -0.03739 | 0.08648 |
|  |  | *Tuesday* | 61 | 0.03215 | 0.08893 |
|  |  | *Wednesday* | 64 | 0.1611 | 0.0857 |
|  |  | *Thursday* | 58 | 0.07381 | 0.07683 |
|  |  | *Friday* | 68 | -0.007734 | 0.08947 |
|  |  | *Saturday* | 56 | 0.1042 | 0.09423 |
|  |  | *Sunday* | 66 | 0.05519 | 0.08926 |
| **BEG** | **Strong, Normal** | *Monday* | 64 | 0.07248 | 0.1291 |
|  |  | *Tuesday* | 55 | 0.01554 | 0.1258 |
|  |  | *Wednesday* | 55 | 0.02722 | 0.1296 |
|  |  | *Thursday* | 57 | 0.1612 | 0.1292 |
|  |  | *Friday* | 48 | 0.07248 | 0.1387 |
|  |  | *Saturday* | 62 | -0.1425 | 0.1246 |
|  |  | *Sunday* | 54 | -0.0435 | 0.1435 |
|  | **Weak** | *Monday* | 54 | -0.09108 | 0.1348 |
|  |  | *Tuesday* | 60 | -0.1577 | 0.1272 |
|  |  | *Wednesday* | 64 | -0.1082 | 0.1257 |
|  |  | *Thursday* | 58 | 0.2469 | 0.1395 |
|  |  | *Friday* | 68 | -0.1117 | 0.1115 |
|  |  | *Saturday* | 56 | 0.1298 | 0.1452 |
|  |  | *Sunday* | 67 | -0.0174 | 0.1278 |
| **BART** | **Strong, Normal** | *Monday* | 64 | 0.02275 | 0.1352 |
|  |  | *Tuesday* | 56 | -0.04724 | 0.125 |
|  |  | *Wednesday* | 56 | -0.005911 | 0.1547 |
|  |  | *Thursday* | 59 | -0.06381 | 0.1262 |
|  |  | *Friday* | 50 | -0.08459 | 0.123 |
|  |  | *Saturday* | 62 | 0.05096 | 0.1377 |
|  |  | *Sunday* | 54 | -0.2678 | 0.1336 |
|  | **Weak** | *Monday* | 54 | 0.01505 | 0.1417 |
|  |  | *Tuesday* | 61 | -0.1489 | 0.1233 |
|  |  | *Wednesday* | 64 | 0.1929 | 0.1296 |
|  |  | *Thursday* | 58 | 0.1485 | 0.1199 |
|  |  | *Friday* | 68 | 0.2019 | 0.1221 |
|  |  | *Saturday* | 56 | -0.03155 | 0.1344 |
|  |  | *Sunday* | 67 | -0.05719 | 0.1099 |

**Supplementary Material Figure I:** The mean scores for each of the four main risk measurements across participants, separated out between a weak (rating of 1 or 2 on a scale of 1 to 5) versus normal or weak (rating of 3, 4, or 5 on the same scale) sense of the week, in Study 2, all normalized using z-scoring. A) SOEP General, B) DOSPERT General, C) BEG, D) Normalized BART scores, scored as per Lejuez et al. (2002) methodology. Error bars represent +/- SE.

| 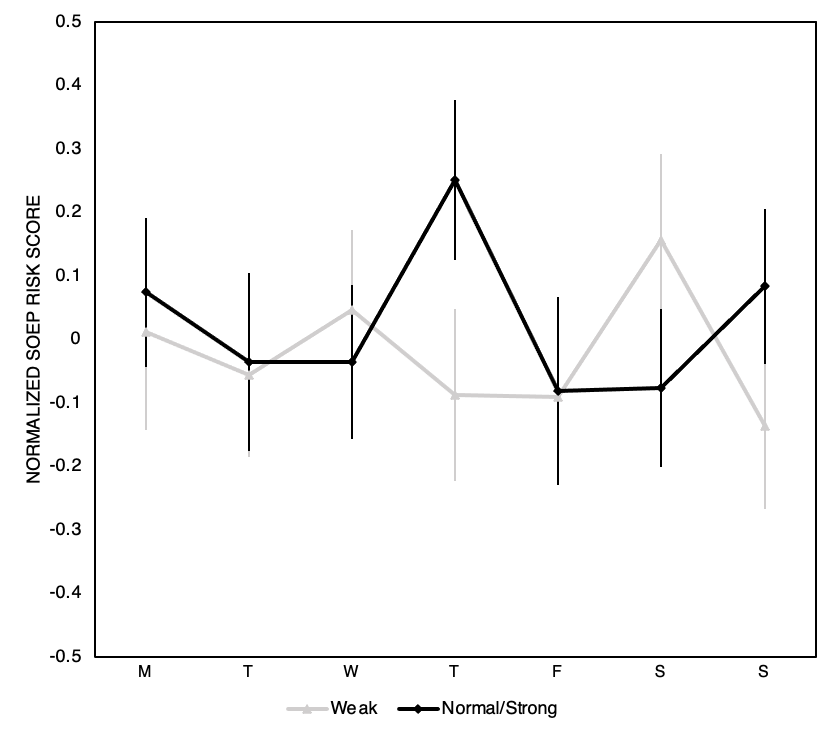 | 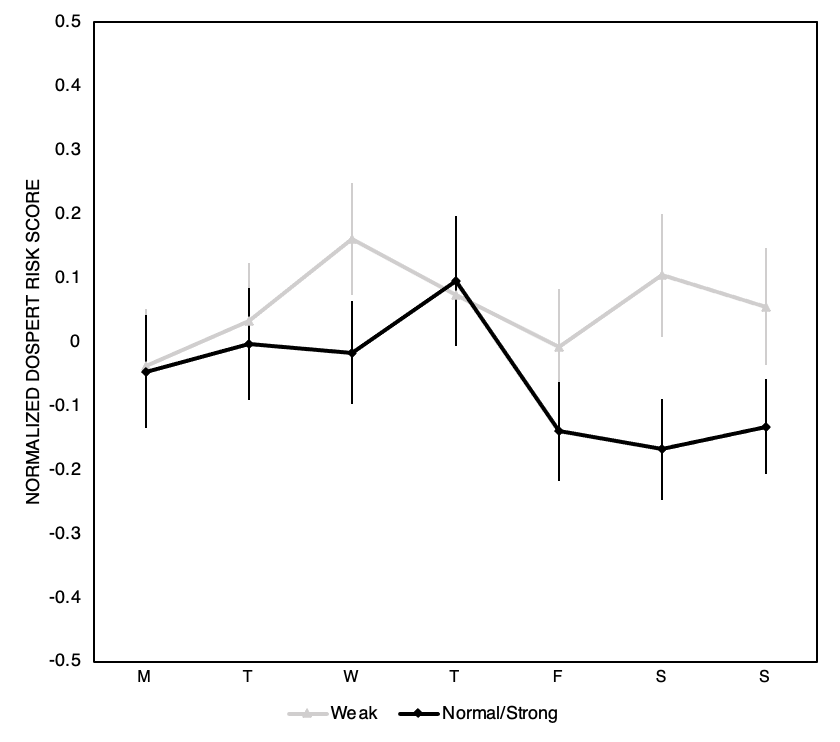 |
| --- | --- |
| 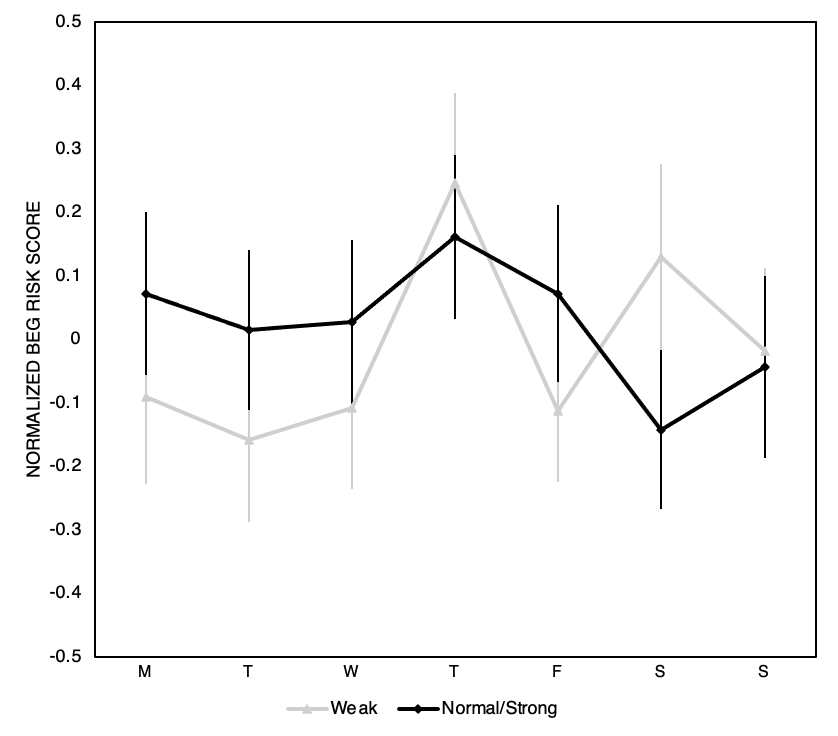 | 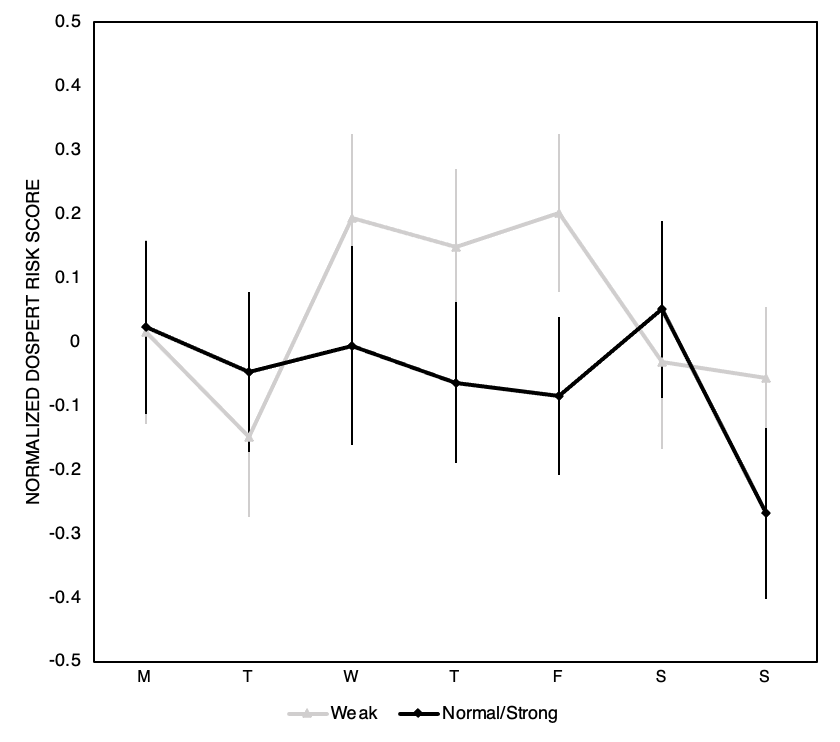  NORMALIZED BART RISK SCORE |

***Supplementary Table G.1:**  Results of generalized linear model for Z-scored SOEP for those with a Normal/Strong SOW of weekday only.

|  |  |  | **95% Confidence Interval** | | |  |  |  |
| --- | --- | --- | --- | --- | --- | --- | --- | --- |
| **Effect** | **Estimate** | **SE** | **Lower** | **Upper** | **β** | **df** | **t** | **p** |
| (Intercept) | 0.026 | 0.049 | -0.07 | 0.121 | 0 | 394 | 0.53 | 0.597 |
| Tue - Mon | -0.11 | 0.178 | -0.459 | 0.239 | -0.113 | 394 | -0.619 | 0.536 |
| Wed - Mon | -0.11 | 0.178 | -0.459 | 0.239 | -0.113 | 394 | -0.619 | 0.536 |
| Thu - Mon | 0.176 | 0.175 | -0.168 | 0.521 | 0.182 | 394 | 1.008 | 0.314 |
| Fri - Mon | -0.155 | 0.183 | -0.515 | 0.205 | -0.16 | 394 | -0.847 | 0.398 |
| Sat - Mon | -0.15 | 0.173 | -0.49 | 0.19 | -0.155 | 394 | -0.869 | 0.385 |
| Sun - Mon | 0.01 | 0.179 | -0.343 | 0.362 | 0.01 | 394 | 0.053 | 0.958 |

*[* p < 0.05; ** p < 0.01, *** p < 0.001]*

***Supplementary Materials Table G.2.:** Results of post-hoc comparisons for Z-scored SOEP for those with a Normal/Strong SOW of weekday only.

| **Weekday** |  | **Weekday** | **Difference** | **SE** | **t** | **df** | **p** | **p_bonferroni_** |
| --- | --- | --- | --- | --- | --- | --- | --- | --- |
| Fri | - | Sat | -0.005 | 0.184 | -0.026 | 394 | 0.98 | 1 |
| Fri | - | Sun | -0.165 | 0.19 | -0.864 | 394 | 0.388 | 1 |
| Mon | - | Fri | 0.155 | 0.183 | 0.847 | 394 | 0.398 | 1 |
| Mon | - | Sat | 0.15 | 0.173 | 0.869 | 394 | 0.385 | 1 |
| Mon | - | Sun | -0.01 | 0.179 | -0.053 | 394 | 0.958 | 1 |
| Mon | - | Wed | 0.11 | 0.178 | 0.619 | 394 | 0.536 | 1 |
| Mon | - | Thu | -0.176 | 0.175 | -1.008 | 394 | 0.314 | 1 |
| Mon | - | Tue | 0.11 | 0.178 | 0.619 | 394 | 0.536 | 1 |
| Sat | - | Sun | -0.16 | 0.181 | -0.885 | 394 | 0.377 | 1 |
| Wed | - | Fri | 0.045 | 0.189 | 0.239 | 394 | 0.811 | 1 |
| Wed | - | Sat | 0.04 | 0.179 | 0.225 | 394 | 0.822 | 1 |
| Wed | - | Sun | -0.119 | 0.185 | -0.646 | 394 | 0.519 | 1 |
| Wed | - | Thu | -0.286 | 0.181 | -1.583 | 394 | 0.114 | 1 |
| Thu | - | Fri | 0.332 | 0.186 | 1.778 | 394 | 0.076 | 1 |
| Thu | - | Sat | 0.327 | 0.176 | 1.852 | 394 | 0.065 | 1 |
| Thu | - | Sun | 0.167 | 0.183 | 0.914 | 394 | 0.361 | 1 |
| Tue | - | Fri | 0.045 | 0.189 | 0.239 | 394 | 0.811 | 1 |
| Tue | - | Sat | 0.04 | 0.179 | 0.225 | 394 | 0.822 | 1 |
| Tue | - | Sun | -0.119 | 0.185 | -0.646 | 394 | 0.519 | 1 |
| Tue | - | Wed | 3.57E-11 | 0.183 | 1.95E-10 | 394 | 1 | 1 |
| Tue | - | Thu | -0.286 | 0.181 | -1.583 | 394 | 0.114 | 1 |

*[* p < 0.05; ** p < 0.01, *** p < 0.001]*

***Supplementary Table G.3:**  Results of generalized linear model for Z-scored SOEP for those with a Normal/Strong SOW of weekday, age, and gender.

|  |  |  | **95% Confidence Interval** | | |  | |  | |  | |  | |
| --- | --- | --- | --- | --- | --- | --- | --- | --- | --- | --- | --- | --- | --- |
| **Effect** | **Estimate** | **SE** | **Lower** | **Upper** | **β** | | **df** | | **t** | | **p** | |  |
| (Intercept) | 0.106 | 0.052 | 0.005 | 0.208 | 0.000 | | 392 | | 2.063 | | 0.04 | |  |
| Age *** | -0.015 | 0.004 | -0.023 | -0.007 | -0.175 | | 392 | | -3.580 | | < .001 | |  |
| Male - Female *** | 0.394 | 0.103 | 0.191 | 0.598 | 0.407 | | 392 | | 3.808 | | < .001 | |  |
| Tue - Mon | -0.186 | 0.173 | -0.525 | 0.154 | -0.192 | | 392 | | -1.076 | | 0.283 | |  |
| Wed - Mon | -0.165 | 0.172 | -0.504 | 0.173 | -0.170 | | 392 | | -0.959 | | 0.338 | |  |
| Thu - Mon | 0.192 | 0.170 | -0.142 | 0.526 | 0.198 | | 392 | | 1.131 | | 0.259 | |  |
| Fri - Mon | -0.111 | 0.178 | -0.460 | 0.238 | -0.115 | | 392 | | -0.626 | | 0.532 | |  |
| Sat - Mon | -0.198 | 0.168 | -0.528 | 0.131 | -0.205 | | 392 | | -1.183 | | 0.238 | |  |
| Sun - Mon | -0.029 | 0.174 | -0.370 | 0.313 | -0.030 | | 392 | | -0.166 | | 0.868 | |  |

*[* p < 0.05; ** p < 0.01, *** p < 0.001]*

***Supplementary Materials Table G.4:** Results of post-hoc comparisons for Z-scored SOEP for those with a Normal/Strong SOW of weekday, age, and gender.

| **Weekday** |  |  | **Difference** | **SE** | **t** | **df** | **p** | **p_bonferroni_** |
| --- | --- | --- | --- | --- | --- | --- | --- | --- |
| Fri | - | Sat | 0.087 | 0.179 | 0.487 | 392 | 0.627 | 1 |
| Fri | - | Sun | -0.082 | 0.185 | -0.445 | 392 | 0.656 | 1 |
| Mon | - | Fri | 0.111 | 0.178 | 0.626 | 392 | 0.532 | 1 |
| Mon | - | Sat | 0.198 | 0.168 | 1.183 | 392 | 0.238 | 1 |
| Mon | - | Sun | 0.029 | 0.174 | 0.166 | 392 | 0.868 | 1 |
| Mon | - | Wed | 0.165 | 0.172 | 0.959 | 392 | 0.338 | 1 |
| Mon | - | Thu | -0.192 | 0.170 | -1.131 | 392 | 0.259 | 1 |
| Mon | - | Tue | 0.186 | 0.173 | 1.076 | 392 | 0.283 | 1 |
| Sat | - | Sun | -0.170 | 0.175 | -0.971 | 392 | 0.332 | 1 |
| Wed | - | Fri | -0.054 | 0.184 | -0.293 | 392 | 0.769 | 1 |
| Wed | - | Sat | 0.033 | 0.173 | 0.193 | 392 | 0.847 | 1 |
| Wed | - | Sun | -0.136 | 0.179 | -0.761 | 392 | 0.447 | 1 |
| Wed | - | Thu | -0.357 | 0.176 | -2.032 | 392 | 0.043 | 0.9 |
| Thu | - | Fri | 0.303 | 0.181 | 1.679 | 392 | 0.094 | 1 |
| Thu | - | Sat | 0.390 | 0.171 | 2.281 | 392 | 0.023 | 0.485 |
| Thu | - | Sun | 0.221 | 0.177 | 1.247 | 392 | 0.213 | 1 |
| Tue | - | Fri | -0.075 | 0.184 | -0.406 | 392 | 0.685 | 1 |
| Tue | - | Sat | 0.013 | 0.173 | 0.073 | 392 | 0.942 | 1 |
| Tue | - | Sun | -0.157 | 0.179 | -0.876 | 392 | 0.382 | 1 |
| Tue | - | Wed | -0.021 | 0.178 | -0.117 | 392 | 0.907 | 1 |
| Tue | - | Thu | -0.378 | 0.176 | -2.147 | 392 | 0.032 | 0.68 |

*[* p < 0.05; ** p < 0.01, *** p < 0.001]*

***Supplementary Table G.5:**  Results of generalized linear model for Z-scored SOEP for those with a Weak SOW of weekday only.

|  |  |  | **95% Confidence Interval** | | |  |  |  |
| --- | --- | --- | --- | --- | --- | --- | --- | --- |
| **Effect** | **Estimate** | **SE** | **Lower** | **Upper** | **β** | **df** | **t** | **p** |
| (Intercept) | -0.022 | 0.05 | -0.12 | 0.076 | 0 | 421 | -0.439 | 0.661 |
| Tue - Mon | -0.067 | 0.193 | -0.446 | 0.311 | -0.066 | 421 | -0.35 | 0.727 |
| Wed - Mon | 0.036 | 0.191 | -0.339 | 0.41 | 0.035 | 421 | 0.186 | 0.852 |
| Thu - Mon | -0.099 | 0.195 | -0.482 | 0.284 | -0.096 | 421 | -0.508 | 0.612 |
| Fri - Mon | -0.102 | 0.188 | -0.471 | 0.268 | -0.099 | 421 | -0.542 | 0.588 |
| Sat - Mon | 0.145 | 0.197 | -0.241 | 0.532 | 0.141 | 421 | 0.74 | 0.46 |
| Sun - Mon | -0.148 | 0.189 | -0.519 | 0.223 | -0.144 | 421 | -0.785 | 0.433 |

*[* p < 0.05; ** p < 0.01, *** p < 0.001]*

***Supplementary Materials Table G.6:** Results of post-hoc comparisons for Z-scored SOEP for those with a Weak SOW of weekday only.

| **Weekday** |  | **Weekday** | **Difference** | **SE** | **t** | **df** | **p** | **p_bonferroni_** |
| --- | --- | --- | --- | --- | --- | --- | --- | --- |
| Fri | - | Sat | -0.247 | 0.186 | -1.329 | 421 | 0.185 | 1 |
| Fri | - | Sun | 0.046 | 0.178 | 0.26 | 421 | 0.795 | 1 |
| Mon | - | Fri | 0.102 | 0.188 | 0.542 | 421 | 0.588 | 1 |
| Mon | - | Sat | -0.145 | 0.197 | -0.74 | 421 | 0.46 | 1 |
| Mon | - | Sun | 0.148 | 0.189 | 0.785 | 421 | 0.433 | 1 |
| Mon | - | Wed | -0.036 | 0.191 | -0.186 | 421 | 0.852 | 1 |
| Mon | - | Thu | 0.099 | 0.195 | 0.508 | 421 | 0.612 | 1 |
| Mon | - | Tue | 0.067 | 0.193 | 0.35 | 421 | 0.727 | 1 |
| Sat | - | Sun | 0.294 | 0.187 | 1.572 | 421 | 0.117 | 1 |
| Wed | - | Fri | 0.137 | 0.18 | 0.765 | 421 | 0.445 | 1 |
| Wed | - | Sat | -0.11 | 0.189 | -0.583 | 421 | 0.56 | 1 |
| Wed | - | Sun | 0.184 | 0.18 | 1.019 | 421 | 0.309 | 1 |
| Wed | - | Thu | 0.135 | 0.187 | 0.72 | 421 | 0.472 | 1 |
| Thu | - | Fri | 0.003 | 0.184 | 0.015 | 421 | 0.988 | 1 |
| Thu | - | Sat | -0.245 | 0.193 | -1.266 | 421 | 0.206 | 1 |
| Thu | - | Sun | 0.049 | 0.185 | 0.265 | 421 | 0.791 | 1 |
| Tue | - | Fri | 0.034 | 0.182 | 0.19 | 421 | 0.85 | 1 |
| Tue | - | Sat | -0.213 | 0.191 | -1.115 | 421 | 0.265 | 1 |
| Tue | - | Sun | 0.081 | 0.183 | 0.442 | 421 | 0.659 | 1 |
| Tue | - | Wed | -0.103 | 0.185 | -0.558 | 421 | 0.577 | 1 |
| Tue | - | Thu | 0.032 | 0.189 | 0.167 | 421 | 0.867 | 1 |

*[* p < 0.05; ** p < 0.01, *** p < 0.001]*

***Supplementary Table G.7:** Results of generalized linear model for Z-scored SOEP for those with a Weak SOW of weekday, age, and gender.

|  |  |  | **95% Confidence Interval** | |  |  |  |  |
| --- | --- | --- | --- | --- | --- | --- | --- | --- |
| **Effect** | **Estimate** | **SE** | **Lower** | **Upper** | **β** | **df** | **t** | **p** |
| (Intercept) | 0.027 | 0.056 | -0.084 | 0.138 | 0.000 | 419 | 0.478 | 0.633 |
| Age* | -0.009 | 0.004 | -0.017 | -2.832e−4 | -0.098 | 419 | -2.032 | 0.043 |
| Male - Female | 0.207 | 0.113 | -0.015 | 0.428 | 0.201 | 419 | 1.833 | 0.068 |
| Tue - Mon | -0.026 | 0.192 | -0.404 | 0.351 | -0.026 | 419 | -0.138 | 0.891 |
| Wed - Mon | 0.022 | 0.189 | -0.350 | 0.395 | 0.022 | 419 | 0.117 | 0.907 |
| Thu - Mon | -0.083 | 0.194 | -0.464 | 0.298 | -0.081 | 419 | -0.429 | 0.668 |
| Fri - Mon | -0.095 | 0.187 | -0.462 | 0.272 | -0.092 | 419 | -0.509 | 0.611 |
| Sat - Mon | 0.163 | 0.196 | -0.222 | 0.547 | 0.158 | 419 | 0.833 | 0.406 |
| Sun - Mon | -0.171 | 0.188 | -0.539 | 0.198 | -0.166 | 419 | -0.910 | 0.364 |

*[* p < 0.05; ** p < 0.01, *** p < 0.001]*

***Supplementary Materials Table G.8.:** Results of post-hoc comparisons for Z-scored SOEP for those with a Weak SOW of weekday, age, and gender.

| **DOW** | | | **Difference** | **SE** | **t** | **df** | **p** | **p_bonferroni_** |
| --- | --- | --- | --- | --- | --- | --- | --- | --- |
| Fri | - | Sat | -0.258 | 0.185 | -1.394 | 419 | 0.164 | 1 |
| Fri | - | Sun | 0.076 | 0.177 | 0.428 | 419 | 0.669 | 1 |
| Mon | - | Fri | 0.095 | 0.187 | 0.509 | 419 | 0.611 | 1 |
| Mon | - | Sat | -0.163 | 0.196 | -0.833 | 419 | 0.406 | 1 |
| Mon | - | Sun | 0.171 | 0.188 | 0.910 | 419 | 0.364 | 1 |
| Mon | - | Wed | -0.022 | 0.189 | -0.117 | 419 | 0.907 | 1 |
| Mon | - | Thu | 0.083 | 0.194 | 0.429 | 419 | 0.668 | 1 |
| Mon | - | Tue | 0.026 | 0.192 | 0.138 | 419 | 0.891 | 1 |
| Sat | - | Sun | 0.333 | 0.186 | 1.791 | 419 | 0.074 | 1 |
| Wed | - | Fri | 0.117 | 0.179 | 0.656 | 419 | 0.512 | 1 |
| Wed | - | Sat | -0.141 | 0.188 | -0.749 | 419 | 0.455 | 1 |
| Wed | - | Sun | 0.193 | 0.179 | 1.076 | 419 | 0.282 | 1 |
| Wed | - | Thu | 0.105 | 0.186 | 0.566 | 419 | 0.572 | 1 |
| Thu | - | Fri | 0.012 | 0.183 | 0.065 | 419 | 0.948 | 1 |
| Thu | - | Sat | -0.246 | 0.192 | -1.281 | 419 | 0.201 | 1 |
| Thu | - | Sun | 0.087 | 0.184 | 0.475 | 419 | 0.635 | 1 |
| Tue | - | Fri | 0.069 | 0.181 | 0.378 | 419 | 0.705 | 1 |
| Tue | - | Sat | -0.189 | 0.190 | -0.997 | 419 | 0.319 | 1 |
| Tue | - | Sun | 0.144 | 0.183 | 0.789 | 419 | 0.431 | 1 |
| Tue | - | Wed | -0.049 | 0.184 | -0.264 | 419 | 0.792 | 1 |
| Tue | - | Thu | 0.057 | 0.188 | 0.301 | 419 | 0.763 | 1 |

*[* p < 0.05; ** p < 0.01, *** p < 0.001]*

***Supplementary Table G.9:**  Results of generalized linear model for Z-scored DOSPERT for those with a Normal/Strong SOW of weekday only.

|  |  |  | **95% Confidence Interval** | | |  |  |  |
| --- | --- | --- | --- | --- | --- | --- | --- | --- |
| **Effect** | **Estimate** | **SE** | **Lower** | **Upper** | **β** | **df** | **t** | **p** |
| (Intercept) | -0.059 | 0.032 | -0.122 | 0.005 | 0 | 394 | -1.82 | 0.07 |
| Tue - Mon | 0.043 | 0.118 | -0.189 | 0.276 | 0.067 | 394 | 0.367 | 0.714 |
| Wed - Mon | 0.03 | 0.118 | -0.202 | 0.262 | 0.046 | 394 | 0.252 | 0.801 |
| Thu - Mon | 0.142 | 0.116 | -0.087 | 0.371 | 0.22 | 394 | 1.219 | 0.224 |
| Fri - Mon | -0.093 | 0.122 | -0.333 | 0.146 | -0.144 | 394 | -0.766 | 0.444 |
| Sat - Mon | -0.122 | 0.115 | -0.348 | 0.104 | -0.189 | 394 | -1.06 | 0.29 |
| Sun - Mon | -0.086 | 0.119 | -0.321 | 0.148 | -0.133 | 394 | -0.724 | 0.47 |

*[* p < 0.05; ** p < 0.01, *** p < 0.001]*

**(Supplementary Materials Table G.10.:** Results of post-hoc comparisons for Z-scored DOSPERT for those with a Normal/Strong SOW of weekday only.

| **Weekday** |  |  | **Difference** | **SE** | **t** | **df** | **p** | **p_bonferroni_** |
| --- | --- | --- | --- | --- | --- | --- | --- | --- |
| Fri | - | Sat | 0.029 | 0.123 | 0.234 | 394 | 0.815 | 1 |
| Fri | - | Sun | -0.007 | 0.127 | -0.055 | 394 | 0.956 | 1 |
| Mon | - | Fri | 0.093 | 0.122 | 0.766 | 394 | 0.444 | 1 |
| Mon | - | Sat | 0.122 | 0.115 | 1.06 | 394 | 0.29 | 1 |
| Mon | - | Sun | 0.086 | 0.119 | 0.724 | 394 | 0.47 | 1 |
| Mon | - | Wed | -0.03 | 0.118 | -0.252 | 394 | 0.801 | 1 |
| Mon | - | Thu | -0.142 | 0.116 | -1.219 | 394 | 0.224 | 1 |
| Mon | - | Tue | -0.043 | 0.118 | -0.367 | 394 | 0.714 | 1 |
| Sat | - | Sun | -0.036 | 0.12 | -0.297 | 394 | 0.767 | 1 |
| Wed | - | Fri | 0.123 | 0.126 | 0.98 | 394 | 0.328 | 1 |
| Wed | - | Sat | 0.152 | 0.119 | 1.275 | 394 | 0.203 | 1 |
| Wed | - | Sun | 0.116 | 0.123 | 0.943 | 394 | 0.346 | 1 |
| Wed | - | Thu | -0.112 | 0.12 | -0.932 | 394 | 0.352 | 1 |
| Thu | - | Fri | 0.235 | 0.124 | 1.896 | 394 | 0.059 | 1 |
| Thu | - | Sat | 0.264 | 0.117 | 2.248 | 394 | 0.025 | 0.527 |
| Thu | - | Sun | 0.228 | 0.122 | 1.878 | 394 | 0.061 | 1 |
| Tue | - | Fri | 0.137 | 0.126 | 1.088 | 394 | 0.277 | 1 |
| Tue | - | Sat | 0.165 | 0.119 | 1.389 | 394 | 0.166 | 1 |
| Tue | - | Sun | 0.13 | 0.123 | 1.053 | 394 | 0.293 | 1 |
| Tue | - | Wed | 0.014 | 0.122 | 0.111 | 394 | 0.912 | 1 |
| Tue | - | Thu | -0.099 | 0.12 | -0.819 | 394 | 0.413 | 1 |

*[* p < 0.05; ** p < 0.01, *** p < 0.001]*

***Supplementary Table G.11:**  Results of generalized linear model for Z-scored DOSPERT for those with a Normal/Strong SOW of weekday, age, and gender.

|  |  |  | **95% Confidence Interval** | |  | |  | |  | |  | |
| --- | --- | --- | --- | --- | --- | --- | --- | --- | --- | --- | --- | --- |
| **Effect** | **Estimate** | **SE** | **Lower** | **Upper** | **β** | **df** | | **t** | | **p** | |  |
| (Intercept) | 0.028 | 0.033 | -0.037 | 0.094 | 0.000 | 392 | | 0.850 | | 0.396 | |  |
| Age ** | -0.008 | 0.003 | -0.013 | -0.002 | -0.136 | 392 | | -2.863 | | 0.004 | |  |
| Male - Female *** | 0.426 | 0.067 | 0.294 | 0.558 | 0.659 | 392 | | 6.349 | | < .001 | |  |
| Tue - Mon | -0.002 | 0.112 | -0.222 | 0.218 | -0.003 | 392 | | -0.017 | | 0.986 | |  |
| Wed - Mon | -0.009 | 0.112 | -0.228 | 0.211 | -0.013 | 392 | | -0.078 | | 0.938 | |  |
| Thu - Mon | 0.170 | 0.110 | -0.046 | 0.387 | 0.264 | 392 | | 1.548 | | 0.123 | |  |
| Fri - Mon | -0.046 | 0.115 | -0.272 | 0.181 | -0.070 | 392 | | -0.395 | | 0.693 | |  |
| Sat - Mon | -0.149 | 0.109 | -0.363 | 0.065 | -0.231 | 392 | | -1.370 | | 0.171 | |  |
| Sun - Mon | -0.107 | 0.113 | -0.328 | 0.115 | -0.165 | 392 | | -0.946 | | 0.345 | |  |

*[* p < 0.05; ** p < 0.01, *** p < 0.001]*

***Supplementary Materials Table G.12:** Results of post-hoc comparisons for Z-scored DOSPERT for those with a Normal/Strong SOW of weekday, age, and gender.

| **Weekday** |  |  | **Difference** | **SE** | **t** | **df** | **p** | **p_bonferroni_** |
| --- | --- | --- | --- | --- | --- | --- | --- | --- |
| Fri | - | Sat | 0.104 | 0.116 | 0.890 | 392 | 0.374 | 1 |
| Fri | - | Sun | 0.061 | 0.120 | 0.510 | 392 | 0.611 | 1 |
| Mon | - | Fri | 0.046 | 0.115 | 0.395 | 392 | 0.693 | 1 |
| Mon | - | Sat | 0.149 | 0.109 | 1.370 | 392 | 0.171 | 1 |
| Mon | - | Sun | 0.107 | 0.113 | 0.946 | 392 | 0.345 | 1 |
| Mon | - | Wed | 0.009 | 0.112 | 0.078 | 392 | 0.938 | 1 |
| Mon | - | Thu | -0.170 | 0.110 | -1.548 | 392 | 0.123 | 1 |
| Mon | - | Tue | 0.002 | 0.112 | 0.017 | 392 | 0.986 | 1 |
| Sat | - | Sun | -0.042 | 0.113 | -0.374 | 392 | 0.708 | 1 |
| Wed | - | Fri | 0.037 | 0.119 | 0.310 | 392 | 0.757 | 1 |
| Wed | - | Sat | 0.140 | 0.112 | 1.251 | 392 | 0.212 | 1 |
| Wed | - | Sun | 0.098 | 0.116 | 0.844 | 392 | 0.399 | 1 |
| Wed | - | Thu | -0.179 | 0.114 | -1.571 | 392 | 0.117 | 1 |
| Thu | - | Fri | 0.216 | 0.117 | 1.844 | 392 | 0.066 | 1 |
| Thu | - | Sat | 0.320 | 0.111 | 2.878 | 392 | 0.004 | 0.089 |
| Thu | - | Sun | 0.277 | 0.115 | 2.412 | 392 | 0.016 | 0.343 |
| Tue | - | Fri | 0.044 | 0.119 | 0.365 | 392 | 0.715 | 1 |
| Tue | - | Sat | 0.147 | 0.112 | 1.310 | 392 | 0.191 | 1 |
| Tue | - | Sun | 0.105 | 0.116 | 0.901 | 392 | 0.368 | 1 |
| Tue | - | Wed | 0.007 | 0.115 | 0.058 | 392 | 0.953 | 1 |
| Tue | - | Thu | -0.172 | 0.114 | -1.510 | 392 | 0.132 | 1 |

*[* p < 0.05; ** p < 0.01, *** p < 0.001]*

***Supplementary Table G.13:**  Results of generalized linear model for Z-scored DOSPERT for those with a Weak SOW of weekday only.

|  |  |  | **95% Confidence Interval** | | |  |  |  |
| --- | --- | --- | --- | --- | --- | --- | --- | --- |
| **Effect** | **Estimate** | **SE** | **Lower** | **Upper** | **β** | **df** | **t** | **p** |
| (Intercept) | 0.054 | 0.033 | -0.011 | 0.12 | 0 | 420 | 1.637 | 0.102 |
| Tue - Mon | 0.07 | 0.128 | -0.182 | 0.321 | 0.102 | 420 | 0.543 | 0.588 |
| Wed - Mon | 0.198 | 0.127 | -0.051 | 0.448 | 0.29 | 420 | 1.567 | 0.118 |
| Thu - Mon | 0.111 | 0.13 | -0.144 | 0.366 | 0.163 | 420 | 0.858 | 0.392 |
| Fri - Mon | 0.03 | 0.125 | -0.216 | 0.275 | 0.043 | 420 | 0.237 | 0.813 |
| Sat - Mon | 0.142 | 0.131 | -0.115 | 0.399 | 0.207 | 420 | 1.083 | 0.279 |
| Sun - Mon | 0.093 | 0.126 | -0.155 | 0.34 | 0.135 | 420 | 0.736 | 0.462 |

*[* p < 0.05; ** p < 0.01, *** p < 0.001]*

***Supplementary Materials Table G.14:** Results of post-hoc comparisons for Z-scored DOSPERT for those with a Weak SOW of weekday only.

| **Weekday** |  | **Weekday** | **Difference** | **SE** | **t** | **df** | **p** | **p_bonferroni_** |
| --- | --- | --- | --- | --- | --- | --- | --- | --- |
| Fri | - | Sat | -0.112 | 0.124 | -0.905 | 420 | 0.366 | 1 |
| Fri | - | Sun | -0.063 | 0.118 | -0.531 | 420 | 0.596 | 1 |
| Mon | - | Fri | -0.03 | 0.125 | -0.237 | 420 | 0.813 | 1 |
| Mon | - | Sat | -0.142 | 0.131 | -1.083 | 420 | 0.279 | 1 |
| Mon | - | Sun | -0.093 | 0.126 | -0.736 | 420 | 0.462 | 1 |
| Mon | - | Wed | -0.198 | 0.127 | -1.567 | 420 | 0.118 | 1 |
| Mon | - | Thu | -0.111 | 0.13 | -0.858 | 420 | 0.392 | 1 |
| Mon | - | Tue | -0.07 | 0.128 | -0.543 | 420 | 0.588 | 1 |
| Sat | - | Sun | 0.049 | 0.125 | 0.394 | 420 | 0.694 | 1 |
| Wed | - | Fri | 0.169 | 0.119 | 1.414 | 420 | 0.158 | 1 |
| Wed | - | Sat | 0.057 | 0.125 | 0.453 | 420 | 0.651 | 1 |
| Wed | - | Sun | 0.106 | 0.12 | 0.88 | 420 | 0.379 | 1 |
| Wed | - | Thu | 0.087 | 0.124 | 0.702 | 420 | 0.483 | 1 |
| Thu | - | Fri | 0.082 | 0.123 | 0.665 | 420 | 0.506 | 1 |
| Thu | - | Sat | -0.03 | 0.128 | -0.237 | 420 | 0.813 | 1 |
| Thu | - | Sun | 0.019 | 0.123 | 0.151 | 420 | 0.88 | 1 |
| Tue | - | Fri | 0.04 | 0.121 | 0.33 | 420 | 0.742 | 1 |
| Tue | - | Sat | -0.072 | 0.127 | -0.568 | 420 | 0.57 | 1 |
| Tue | - | Sun | -0.023 | 0.122 | -0.189 | 420 | 0.85 | 1 |
| Tue | - | Wed | -0.129 | 0.123 | -1.051 | 420 | 0.294 | 1 |
| Tue | - | Thu | -0.042 | 0.126 | -0.331 | 420 | 0.741 | 1 |

*[* p < 0.05; ** p < 0.01, *** p < 0.001]*

***Supplementary Table G.15:** Results of generalized linear model for Z-scored DOSPERT for those with a Weak SOW of weekday, age, and gender.

|  |  |  | **95% Confidence Interval** | | |  |  | | |  |  | | |
| --- | --- | --- | --- | --- | --- | --- | --- | --- | --- | --- | --- | --- | --- |
| **Effect** | **Estimate** | **SE** | **Lower** | **Upper** | **β** | | | **df** | **t** | | | **p** |  |
| (Intercept) | 0.144 | 0.036 | 0.073 | 0.214 | 0.000 | | | 418 | 4.014 | | | < .001 |  |
| Age *** | -0.012 | 0.003 | -0.017 | -0.006 | -0.201 | | | 418 | -4.338 | | | < .001 |  |
| Male - Female *** | 0.379 | 0.072 | 0.238 | 0.520 | 0.555 | | | 418 | 5.289 | | | < .001 |  |
| Tue - Mon | 0.135 | 0.122 | -0.105 | 0.375 | 0.197 | | | 418 | 1.104 | | | 0.270 |  |
| Wed - Mon | 0.179 | 0.120 | -0.058 | 0.415 | 0.261 | | | 418 | 1.482 | | | 0.139 |  |
| Thu - Mon | 0.136 | 0.123 | -0.106 | 0.379 | 0.200 | | | 418 | 1.107 | | | 0.269 |  |
| Fri - Mon | 0.039 | 0.119 | -0.195 | 0.272 | 0.057 | | | 418 | 0.328 | | | 0.743 |  |
| Sat - Mon | 0.170 | 0.124 | -0.075 | 0.414 | 0.248 | | | 418 | 1.365 | | | 0.173 |  |
| Sun - Mon | 0.052 | 0.120 | -0.183 | 0.287 | 0.076 | | | 418 | 0.435 | | | 0.664 |  |

*[* p < 0.05; ** p < 0.01, *** p < 0.001]*

***Supplementary Materials Table G.16.:** Results of post-hoc comparisons for Z-scored DOSPERT for those with a Weak SOW of weekday, age, and gender.

| **DOW** |  | **DOW** | **Difference** | **SE** | **t** | **df** | **p** | **p_bonferroni_** |
| --- | --- | --- | --- | --- | --- | --- | --- | --- |
| Fri | - | Sat | -0.131 | 0.118 | -1.112 | 418 | 0.267 | 1 |
| Fri | - | Sun | -0.013 | 0.113 | -0.116 | 418 | 0.907 | 1 |
| Mon | - | Fri | -0.039 | 0.119 | -0.327 | 418 | 0.743 | 1 |
| Mon | - | Sat | -0.170 | 0.124 | -1.365 | 418 | 0.173 | 1 |
| Mon | - | Sun | -0.052 | 0.120 | -0.435 | 418 | 0.664 | 1 |
| Mon | - | Wed | -0.179 | 0.120 | -1.482 | 418 | 0.139 | 1 |
| Mon | - | Thu | -0.136 | 0.123 | -1.107 | 418 | 0.269 | 1 |
| Mon | - | Tue | -0.135 | 0.122 | -1.104 | 418 | 0.27 | 1 |
| Sat | - | Sun | 0.118 | 0.119 | 0.990 | 418 | 0.323 | 1 |
| Wed | - | Fri | 0.140 | 0.114 | 1.229 | 418 | 0.22 | 1 |
| Wed | - | Sat | 0.009 | 0.119 | 0.074 | 418 | 0.941 | 1 |
| Wed | - | Sun | 0.127 | 0.114 | 1.106 | 418 | 0.269 | 1 |
| Wed | - | Thu | 0.042 | 0.118 | 0.356 | 418 | 0.722 | 1 |
| Thu | - | Fri | 0.098 | 0.117 | 0.837 | 418 | 0.403 | 1 |
| Thu | - | Sat | -0.033 | 0.122 | -0.272 | 418 | 0.785 | 1 |
| Thu | - | Sun | 0.084 | 0.118 | 0.717 | 418 | 0.474 | 1 |
| Tue | - | Fri | 0.096 | 0.115 | 0.833 | 418 | 0.405 | 1 |
| Tue | - | Sat | -0.035 | 0.121 | -0.288 | 418 | 0.773 | 1 |
| Tue | - | Sun | 0.083 | 0.117 | 0.709 | 418 | 0.478 | 1 |
| Tue | - | Wed | -0.044 | 0.117 | -0.373 | 418 | 0.71 | 1 |
| Tue | - | Thu | -0.002 | 0.120 | -0.013 | 418 | 0.99 | 1 |

*[* p < 0.05; ** p < 0.01, *** p < 0.001]*

***Supplementary Table G.17:**  Results of generalized linear model for Z-scored BEG for those with a Normal/Strong SOW of weekday only.

|  |  |  | **95% Confidence Interval** | | |  |  |  |
| --- | --- | --- | --- | --- | --- | --- | --- | --- |
| **Effect** | **Estimate** | **SE** | **Lower** | **Upper** | **β** | **df** | **t** | **p** |
| (Intercept) | 0.023 | 0.05 | -0.075 | 0.121 | 0 | 388 | 0.467 | 0.641 |
| Tue - Mon | -0.057 | 0.182 | -0.414 | 0.3 | -0.058 | 388 | -0.314 | 0.754 |
| Wed - Mon | -0.045 | 0.182 | -0.402 | 0.312 | -0.046 | 388 | -0.249 | 0.803 |
| Thu - Mon | 0.089 | 0.18 | -0.265 | 0.442 | 0.09 | 388 | 0.493 | 0.622 |
| Fri - Mon | -6.250e−11 | 0.189 | -0.371 | 0.371 | -6.350e−11 | 388 | -3.315e−10 | 1 |
| Sat - Mon | -0.215 | 0.176 | -0.561 | 0.131 | -0.218 | 388 | -1.222 | 0.223 |
| Sun - Mon | -0.116 | 0.182 | -0.475 | 0.243 | -0.118 | 388 | -0.636 | 0.525 |

*[* p < 0.05; ** p < 0.01, *** p < 0.001]*

***Supplementary Materials Table G.18.:** Results of post-hoc comparisons for Z-scored BEG for those with a Normal/Strong SOW of weekday only.

|  |  | **Weekday** | **Difference** | **SE** | **t** | **df** | **p** | **p_bonferroni_** |
| --- | --- | --- | --- | --- | --- | --- | --- | --- |
| Fri | - | Sat | 0.215 | 0.19 | 1.132 | 388 | 0.258 | 1 |
| Fri | - | Sun | 0.116 | 0.196 | 0.592 | 388 | 0.554 | 1 |
| Mon | - | Fri | 6.25E-11 | 0.189 | 3.32E-10 | 388 | 1 | 1 |
| Mon | - | Sat | 0.215 | 0.176 | 1.222 | 388 | 0.223 | 1 |
| Mon | - | Sun | 0.116 | 0.182 | 0.636 | 388 | 0.525 | 1 |
| Mon | - | Wed | 0.045 | 0.182 | 0.249 | 388 | 0.803 | 1 |
| Mon | - | Thu | -0.089 | 0.18 | -0.493 | 388 | 0.622 | 1 |
| Mon | - | Tue | 0.057 | 0.182 | 0.314 | 388 | 0.754 | 1 |
| Sat | - | Sun | -0.099 | 0.184 | -0.539 | 388 | 0.591 | 1 |
| Wed | - | Fri | -0.045 | 0.195 | -0.232 | 388 | 0.817 | 1 |
| Wed | - | Sat | 0.17 | 0.183 | 0.928 | 388 | 0.354 | 1 |
| Wed | - | Sun | 0.071 | 0.189 | 0.374 | 388 | 0.709 | 1 |
| Wed | - | Thu | -0.134 | 0.187 | -0.718 | 388 | 0.473 | 1 |
| Thu | - | Fri | 0.089 | 0.193 | 0.459 | 388 | 0.647 | 1 |
| Thu | - | Sat | 0.304 | 0.181 | 1.676 | 388 | 0.095 | 1 |
| Thu | - | Sun | 0.205 | 0.188 | 1.092 | 388 | 0.276 | 1 |
| Tue | - | Fri | -0.057 | 0.195 | -0.292 | 388 | 0.771 | 1 |
| Tue | - | Sat | 0.158 | 0.183 | 0.864 | 388 | 0.388 | 1 |
| Tue | - | Sun | 0.059 | 0.189 | 0.312 | 388 | 0.755 | 1 |
| Tue | - | Wed | -0.012 | 0.188 | -0.062 | 388 | 0.951 | 1 |
| Tue | - | Thu | -0.146 | 0.187 | -0.78 | 388 | 0.436 | 1 |

***Supplementary Table G.19:**  Results of generalized linear model for Z-scored BEG for those with a Normal/Strong SOW of weekday, age, and gender.

|  |  |  | **95% Confidence Interval** | | |  |  |  |  |
| --- | --- | --- | --- | --- | --- | --- | --- | --- | --- |
| **Effect** | **Estimate** | **SE** | **Lower** | **Upper** | **β** | | **df** | **t** | **p** |
| (Intercept) | 0.038 | 0.055 | -0.069 | 0.146 | 0.000 | | 386 | 0.698 | 0.486 |
| Age | 0.001 | 0.004 | -0.008 | 0.009 | 0.008 | | 386 | 0.147 | 0.883 |
| Male - Female | 0.073 | 0.110 | -0.142 | 0.289 | 0.075 | | 386 | 0.670 | 0.503 |
| Tue - Mon | -0.056 | 0.183 | -0.416 | 0.303 | -0.057 | | 386 | -0.309 | 0.758 |
| Wed - Mon | -0.046 | 0.182 | -0.404 | 0.312 | -0.047 | | 386 | -0.253 | 0.801 |
| Thu - Mon | 0.096 | 0.181 | -0.259 | 0.451 | 0.097 | | 386 | 0.530 | 0.596 |
| Fri - Mon | 0.008 | 0.189 | -0.365 | 0.380 | 0.008 | | 386 | 0.040 | 0.968 |
| Sat - Mon | -0.214 | 0.177 | -0.561 | 0.134 | -0.217 | | 386 | -1.209 | 0.227 |
| Sun - Mon | -0.115 | 0.183 | -0.475 | 0.246 | -0.116 | | 386 | -0.625 | 0.532 |

*[* p < 0.05; ** p < 0.01, *** p < 0.001]*

***Supplementary Materials Table G.20:** Results of post-hoc comparisons for Z-scored BEG for those with a Normal/Strong SOW of weekday, age, and gender.

| **Weekday** |  |  | **Difference** | **SE** | **t** | **df** | **p** | **p_bonferroni_** |
| --- | --- | --- | --- | --- | --- | --- | --- | --- |
| Fri | - | Sat | 0.221 | 0.191 | 1.159 | 386 | 0.247 | 1 |
| Fri | - | Sun | 0.122 | 0.197 | 0.620 | 386 | 0.535 | 1 |
| Mon | - | Fri | -0.008 | 0.189 | -0.040 | 386 | 0.968 | 1 |
| Mon | - | Sat | 0.214 | 0.177 | 1.209 | 386 | 0.227 | 1 |
| Mon | - | Sun | 0.115 | 0.183 | 0.625 | 386 | 0.532 | 1 |
| Mon | - | Wed | 0.046 | 0.182 | 0.253 | 386 | 0.801 | 1 |
| Mon | - | Thu | -0.096 | 0.181 | -0.530 | 386 | 0.596 | 1 |
| Mon | - | Tue | 0.056 | 0.183 | 0.309 | 386 | 0.758 | 1 |
| Sat | - | Sun | -0.099 | 0.184 | -0.539 | 386 | 0.590 | 1 |
| Wed | - | Fri | -0.054 | 0.196 | -0.274 | 386 | 0.785 | 1 |
| Wed | - | Sat | 0.168 | 0.183 | 0.915 | 386 | 0.361 | 1 |
| Wed | - | Sun | 0.068 | 0.190 | 0.361 | 386 | 0.718 | 1 |
| Wed | - | Thu | -0.142 | 0.188 | -0.756 | 386 | 0.450 | 1 |
| Thu | - | Fri | 0.088 | 0.194 | 0.454 | 386 | 0.650 | 1 |
| Thu | - | Sat | 0.310 | 0.182 | 1.701 | 386 | 0.090 | 1 |
| Thu | - | Sun | 0.210 | 0.188 | 1.117 | 386 | 0.265 | 1 |
| Tue | - | Fri | -0.064 | 0.197 | -0.326 | 386 | 0.745 | 1 |
| Tue | - | Sat | 0.157 | 0.183 | 0.858 | 386 | 0.392 | 1 |
| Tue | - | Sun | 0.058 | 0.190 | 0.306 | 386 | 0.760 | 1 |
| Tue | - | Wed | -0.010 | 0.189 | -0.055 | 386 | 0.956 | 1 |
| Tue | - | Thu | -0.152 | 0.188 | -0.810 | 386 | 0.418 | 1 |

*[* p < 0.05; ** p < 0.01, *** p < 0.001]*

***Supplementary Table G.21:**  Results of generalized linear model for Z-scored BEG for those with a Weak SOW of weekday only.

|  |  |  | **95% Confidence Interval** | | |  |  |  |
| --- | --- | --- | --- | --- | --- | --- | --- | --- |
| **Effect** | **Estimate** | **SE** | **Lower** | **Upper** | **β** | **df** | **t** | **p** |
| (Intercept) | -0.016 | 0.049 | -0.112 | 0.081 | 0 | 420 | -0.318 | 0.751 |
| Tue - Mon | -0.067 | 0.19 | -0.44 | 0.307 | -0.066 | 420 | -0.351 | 0.726 |
| Wed - Mon | -0.017 | 0.187 | -0.385 | 0.351 | -0.017 | 420 | -0.091 | 0.927 |
| Thu - Mon | 0.338 | 0.192 | -0.039 | 0.715 | 0.333 | 420 | 1.764 | 0.078 |
| Fri - Mon | -0.021 | 0.185 | -0.384 | 0.342 | -0.02 | 420 | -0.112 | 0.911 |
| Sat - Mon | 0.221 | 0.193 | -0.159 | 0.601 | 0.218 | 420 | 1.143 | 0.254 |
| Sun - Mon | 0.074 | 0.185 | -0.29 | 0.438 | 0.073 | 420 | 0.398 | 0.691 |

*[* p < 0.05; ** p < 0.01, *** p < 0.001]*

***Supplementary Materials Table G.22:** Results of post-hoc comparisons for Z-scored BEG for those with a Weak SOW of weekday only.

| **Weekday** |  | **Weekday** | **Difference** | **SE** | **t** | **df** | **p** | **p_bonferroni_** |
| --- | --- | --- | --- | --- | --- | --- | --- | --- |
| Fri | - | Sat | -0.242 | 0.183 | -1.321 | 420 | 0.187 | 1 |
| Fri | - | Sun | -0.094 | 0.174 | -0.541 | 420 | 0.589 | 1 |
| Mon | - | Fri | 0.021 | 0.185 | 0.112 | 420 | 0.911 | 1 |
| Mon | - | Sat | -0.221 | 0.193 | -1.143 | 420 | 0.254 | 1 |
| Mon | - | Sun | -0.074 | 0.185 | -0.398 | 420 | 0.691 | 1 |
| Mon | - | Wed | 0.017 | 0.187 | 0.091 | 420 | 0.927 | 1 |
| Mon | - | Thu | -0.338 | 0.192 | -1.764 | 420 | 0.078 | 1 |
| Mon | - | Tue | 0.067 | 0.19 | 0.351 | 420 | 0.726 | 1 |
| Sat | - | Sun | 0.147 | 0.183 | 0.803 | 420 | 0.423 | 1 |
| Wed | - | Fri | 0.004 | 0.176 | 0.02 | 420 | 0.984 | 1 |
| Wed | - | Sat | -0.238 | 0.185 | -1.284 | 420 | 0.2 | 1 |
| Wed | - | Sun | -0.091 | 0.177 | -0.513 | 420 | 0.608 | 1 |
| Wed | - | Thu | -0.355 | 0.184 | -1.933 | 420 | 0.054 | 1 |
| Thu | - | Fri | 0.359 | 0.181 | 1.981 | 420 | 0.048 | 1 |
| Thu | - | Sat | 0.117 | 0.19 | 0.617 | 420 | 0.538 | 1 |
| Thu | - | Sun | 0.264 | 0.182 | 1.455 | 420 | 0.147 | 1 |
| Tue | - | Fri | -0.046 | 0.179 | -0.256 | 420 | 0.798 | 1 |
| Tue | - | Sat | -0.288 | 0.188 | -1.527 | 420 | 0.127 | 1 |
| Tue | - | Sun | -0.14 | 0.18 | -0.779 | 420 | 0.436 | 1 |
| Tue | - | Wed | -0.05 | 0.182 | -0.272 | 420 | 0.786 | 1 |
| Tue | - | Thu | -0.405 | 0.187 | -2.169 | 420 | 0.031 | 0.644 |

*[* p < 0.05; ** p < 0.01, *** p < 0.001]*

***Supplementary Table G.23:** Results of generalized linear model for Z-scored BEG for those with a Weak SOW of weekday, age, and gender.

|  |  |  | **95% Confidence Interval** | |  |  |  |  |
| --- | --- | --- | --- | --- | --- | --- | --- | --- |
| **Effect** | **Estimate** | **SE** | **Lower** | **Upper** | **β** | **df** | **t** | **p** |
| (Intercept) | 0.020 | 0.056 | -0.089 | 0.130 | 0.000 | 418 | 0.362 | 0.717 |
| Age | 0.001 | 0.004 | -0.007 | 0.010 | 0.014 | 418 | 0.297 | 0.766 |
| Male - Female | 0.152 | 0.111 | -0.067 | 0.372 | 0.150 | 418 | 1.368 | 0.172 |
| Tue - Mon | -0.055 | 0.191 | -0.430 | 0.319 | -0.055 | 418 | -0.291 | 0.771 |
| Wed - Mon | -0.019 | 0.187 | -0.387 | 0.349 | -0.018 | 418 | -0.100 | 0.921 |
| Thu - Mon | 0.342 | 0.192 | -0.034 | 0.719 | 0.337 | 418 | 1.786 | 0.075 |
| Fri - Mon | -0.022 | 0.185 | -0.385 | 0.341 | -0.022 | 418 | -0.119 | 0.906 |
| Sat - Mon | 0.227 | 0.193 | -0.153 | 0.607 | 0.223 | 418 | 1.173 | 0.242 |
| Sun - Mon | 0.065 | 0.185 | -0.300 | 0.430 | 0.064 | 418 | 0.350 | 0.726 |
|  |  |  |  |  |  |  |  |  |

*[* p < 0.05; ** p < 0.01, *** p < 0.001]*

***Supplementary Materials Table G.24.:** Results of post-hoc comparisons for Z-scored BEG for those with a Weak SOW of weekday, age, and gender.

| **DOW** |  |  | **Difference** | **SE** | **t** | **df** | **p** | **p_bonferroni_** |
| --- | --- | --- | --- | --- | --- | --- | --- | --- |
| Fri | - | Sat | -0.249 | 0.183 | -1.360 | 418 | 0.175 | 1 |
| Fri | - | Sun | -0.087 | 0.175 | -0.498 | 418 | 0.619 | 1 |
| Mon | - | Fri | 0.022 | 0.185 | 0.119 | 418 | 0.906 | 1 |
| Mon | - | Sat | -0.227 | 0.193 | -1.173 | 418 | 0.242 | 1 |
| Mon | - | Sun | -0.065 | 0.186 | -0.350 | 418 | 0.726 | 1 |
| Mon | - | Wed | 0.019 | 0.187 | 0.100 | 418 | 0.921 | 1 |
| Mon | - | Thu | -0.342 | 0.192 | -1.786 | 418 | 0.075 | 1 |
| Mon | - | Tue | 0.055 | 0.191 | 0.291 | 418 | 0.771 | 1 |
| Sat | - | Sun | 0.162 | 0.184 | 0.879 | 418 | 0.38 | 1 |
| Wed | - | Fri | 0.003 | 0.177 | 0.019 | 418 | 0.985 | 1 |
| Wed | - | Sat | -0.245 | 0.186 | -1.321 | 418 | 0.187 | 1 |
| Wed | - | Sun | -0.084 | 0.177 | -0.472 | 418 | 0.637 | 1 |
| Wed | - | Thu | -0.361 | 0.184 | -1.962 | 418 | 0.05 | 1 |
| Thu | - | Fri | 0.364 | 0.181 | 2.011 | 418 | 0.045 | 0.944 |
| Thu | - | Sat | 0.116 | 0.190 | 0.609 | 418 | 0.543 | 1 |
| Thu | - | Sun | 0.277 | 0.182 | 1.522 | 418 | 0.129 | 1 |
| Tue | - | Fri | -0.033 | 0.180 | -0.186 | 418 | 0.852 | 1 |
| Tue | - | Sat | -0.282 | 0.189 | -1.497 | 418 | 0.135 | 1 |
| Tue | - | Sun | -0.120 | 0.182 | -0.663 | 418 | 0.508 | 1 |
| Tue | - | Wed | -0.037 | 0.183 | -0.201 | 418 | 0.841 | 1 |
| Tue | - | Thu | -0.398 | 0.187 | -2.129 | 418 | 0.034 | 0.710 |

*[* p < 0.05; ** p < 0.01, *** p < 0.001]*

***Supplementary Table G.25:**  Results of generalized linear model for Z-scored BART for those with a Normal/Strong SOW of weekday only.

|  |  |  | **95% Confidence Interval** | | |  |  |  |
| --- | --- | --- | --- | --- | --- | --- | --- | --- |
| **Effect** | **Estimate** | **SE** | **Lower** | **Upper** | **β** | **df** | **t** | **p** |
| (Intercept) | -0.057 | 0.051 | -0.157 | 0.044 | 0 | 394 | -1.107 | 0.269 |
| Tue - Mon | -0.07 | 0.187 | -0.437 | 0.297 | -0.069 | 394 | -0.375 | 0.708 |
| Wed - Mon | -0.029 | 0.187 | -0.396 | 0.338 | -0.028 | 394 | -0.154 | 0.878 |
| Thu - Mon | -0.087 | 0.184 | -0.448 | 0.275 | -0.085 | 394 | -0.47 | 0.638 |
| Fri - Mon | -0.107 | 0.192 | -0.486 | 0.271 | -0.106 | 394 | -0.558 | 0.577 |
| Sat - Mon | 0.028 | 0.182 | -0.329 | 0.386 | 0.028 | 394 | 0.155 | 0.877 |
| Sun - Mon | -0.291 | 0.188 | -0.661 | 0.08 | -0.286 | 394 | -1.542 | 0.124 |

*[* p < 0.05; ** p < 0.01, *** p < 0.001]*

***Supplementary Materials Table G.26.:** Results of post-hoc comparisons for Z-scored BART for those with a Normal/Strong SOW of weekday only.

| **Weekday** |  | **Weekday** | **Difference** | **SE** | **t** | **df** | **p** | **p_bonferroni_** |
| --- | --- | --- | --- | --- | --- | --- | --- | --- |
| Fri | - | Sat | -0.136 | 0.194 | -0.699 | 394 | 0.485 | 1 |
| Fri | - | Sun | 0.183 | 0.2 | 0.916 | 394 | 0.36 | 1 |
| Mon | - | Fri | 0.107 | 0.192 | 0.558 | 394 | 0.577 | 1 |
| Mon | - | Sat | -0.028 | 0.182 | -0.155 | 394 | 0.877 | 1 |
| Mon | - | Sun | 0.291 | 0.188 | 1.542 | 394 | 0.124 | 1 |
| Mon | - | Wed | 0.029 | 0.187 | 0.154 | 394 | 0.878 | 1 |
| Mon | - | Thu | 0.087 | 0.184 | 0.47 | 394 | 0.638 | 1 |
| Mon | - | Tue | 0.07 | 0.187 | 0.375 | 394 | 0.708 | 1 |
| Sat | - | Sun | 0.319 | 0.19 | 1.679 | 394 | 0.094 | 1 |
| Wed | - | Fri | 0.079 | 0.198 | 0.396 | 394 | 0.692 | 1 |
| Wed | - | Sat | -0.057 | 0.188 | -0.302 | 394 | 0.762 | 1 |
| Wed | - | Sun | 0.262 | 0.195 | 1.347 | 394 | 0.179 | 1 |
| Wed | - | Thu | 0.058 | 0.19 | 0.304 | 394 | 0.761 | 1 |
| Thu | - | Fri | 0.021 | 0.196 | 0.106 | 394 | 0.916 | 1 |
| Thu | - | Sat | -0.115 | 0.185 | -0.619 | 394 | 0.536 | 1 |
| Thu | - | Sun | 0.204 | 0.192 | 1.062 | 394 | 0.289 | 1 |
| Tue | - | Fri | 0.037 | 0.198 | 0.188 | 394 | 0.851 | 1 |
| Tue | - | Sat | -0.098 | 0.188 | -0.522 | 394 | 0.602 | 1 |
| Tue | - | Sun | 0.221 | 0.195 | 1.134 | 394 | 0.257 | 1 |
| Tue | - | Wed | -0.041 | 0.193 | -0.214 | 394 | 0.83 | 1 |
| Tue | - | Thu | 0.017 | 0.19 | 0.087 | 394 | 0.931 | 1 |

*[* p < 0.05; ** p < 0.01, *** p < 0.001]*

***Supplementary Table G.27:**  Results of generalized linear model for Z-scored BART for those with a Normal/Strong SOW of weekday, age, and gender.

|  |  |  | **95% Confidence Interval** | | |  | |  | |  | |  | |
| --- | --- | --- | --- | --- | --- | --- | --- | --- | --- | --- | --- | --- | --- |
| **Effect** | **Estimate** | **SE** | **Lower** | **Upper** | **β** | | **df** | | **t** | | **p** | |  |
| (Intercept) | -0.098 | 0.056 | -0.208 | 0.012 | 0.000 | | 392 | | -1.758 | | 0.08 | |  |
| Age | -0.001 | 0.004 | -0.010 | 0.008 | -0.013 | | 392 | | -0.253 | | 0.801 | |  |
| Male - Female | -0.204 | 0.112 | -0.425 | 0.016 | -0.201 | | 392 | | -1.819 | | 0.07 | |  |
| Tue - Mon | -0.070 | 0.187 | -0.438 | 0.299 | -0.068 | | 392 | | -0.371 | | 0.711 | |  |
| Wed - Mon | -0.022 | 0.187 | -0.389 | 0.345 | -0.022 | | 392 | | -0.120 | | 0.904 | |  |
| Thu - Mon | -0.107 | 0.184 | -0.469 | 0.255 | -0.105 | | 392 | | -0.581 | | 0.562 | |  |
| Fri - Mon | -0.130 | 0.193 | -0.509 | 0.248 | -0.128 | | 392 | | -0.677 | | 0.499 | |  |
| Sat - Mon | 0.027 | 0.182 | -0.331 | 0.385 | 0.026 | | 392 | | 0.148 | | 0.883 | |  |
| Sun - Mon | -0.293 | 0.188 | -0.664 | 0.078 | -0.288 | | 392 | | -1.555 | | 0.121 | |  |

*[* p < 0.05; ** p < 0.01, *** p < 0.001]*

***Supplementary Materials Table G.28:** Results of post-hoc comparisons for Z-scored BART for those with a Normal/Strong SOW of weekday, age, and gender.

| **Weekday** |  |  | **Difference** | **SE** | **t** | **df** | **p** | **p_bonferroni_** |
| --- | --- | --- | --- | --- | --- | --- | --- | --- |
| Fri | - | Sat | -0.157 | 0.194 | -0.809 | 392 | 0.419 | 1 |
| Fri | - | Sun | 0.163 | 0.201 | 0.811 | 392 | 0.418 | 1 |
| Mon | - | Fri | 0.130 | 0.193 | 0.677 | 392 | 0.499 | 1 |
| Mon | - | Sat | -0.027 | 0.182 | -0.148 | 392 | 0.883 | 1 |
| Mon | - | Sun | 0.293 | 0.189 | 1.555 | 392 | 0.121 | 1 |
| Mon | - | Wed | 0.022 | 0.187 | 0.120 | 392 | 0.904 | 1 |
| Mon | - | Thu | 0.107 | 0.184 | 0.581 | 392 | 0.562 | 1 |
| Mon | - | Tue | 0.070 | 0.187 | 0.371 | 392 | 0.711 | 1 |
| Sat | - | Sun | 0.320 | 0.190 | 1.688 | 392 | 0.092 | 1 |
| Wed | - | Fri | 0.108 | 0.199 | 0.542 | 392 | 0.588 | 1 |
| Wed | - | Sat | -0.049 | 0.188 | -0.263 | 392 | 0.793 | 1 |
| Wed | - | Sun | 0.271 | 0.194 | 1.393 | 392 | 0.164 | 1 |
| Wed | - | Thu | 0.085 | 0.191 | 0.444 | 392 | 0.658 | 1 |
| Thu | - | Fri | 0.023 | 0.196 | 0.119 | 392 | 0.905 | 1 |
| Thu | - | Sat | -0.134 | 0.186 | -0.721 | 392 | 0.471 | 1 |
| Thu | - | Sun | 0.186 | 0.192 | 0.968 | 392 | 0.333 | 1 |
| Tue | - | Fri | 0.061 | 0.200 | 0.305 | 392 | 0.761 | 1 |
| Tue | - | Sat | -0.096 | 0.188 | -0.513 | 392 | 0.608 | 1 |
| Tue | - | Sun | 0.223 | 0.194 | 1.150 | 392 | 0.251 | 1 |
| Tue | - | Wed | -0.047 | 0.193 | -0.245 | 392 | 0.807 | 1 |
| Tue | - | Thu | 0.037 | 0.191 | 0.196 | 392 | 0.845 | 1 |

*[* p < 0.05; ** p < 0.01, *** p < 0.001]*

***Supplementary Table G.29:**  Results of generalized linear model for Z-scored BART for those with a Weak SOW of weekday only.

|  |  |  | **95% Confidence Interval** | | |  |  |  |
| --- | --- | --- | --- | --- | --- | --- | --- | --- |
| **Effect** | **Estimate** | **SE** | **Lower** | **Upper** | **β** | **df** | **t** | **p** |
| (Intercept) | 0.046 | 0.048 | -0.048 | 0.139 | 0 | 421 | 0.963 | 0.336 |
| Tue - Mon | -0.164 | 0.183 | -0.524 | 0.196 | -0.167 | 421 | -0.894 | 0.372 |
| Wed - Mon | 0.178 | 0.181 | -0.179 | 0.534 | 0.181 | 421 | 0.981 | 0.327 |
| Thu - Mon | 0.133 | 0.186 | -0.231 | 0.498 | 0.136 | 421 | 0.719 | 0.472 |
| Fri - Mon | 0.187 | 0.179 | -0.165 | 0.538 | 0.19 | 421 | 1.045 | 0.297 |
| Sat - Mon | -0.047 | 0.187 | -0.414 | 0.321 | -0.047 | 421 | -0.249 | 0.803 |
| Sun - Mon | -0.072 | 0.179 | -0.425 | 0.28 | -0.074 | 421 | -0.403 | 0.687 |

*[* p < 0.05; ** p < 0.01, *** p < 0.001]*

***Supplementary Materials Table G.30:** Results of post-hoc comparisons for Z-scored BART for those with a Weak SOW of weekday only.

| **Weekday** |  | **Weekday** | **Difference** | **SE** | **t** | **df** | **p** | **p_bonferroni_** |
| --- | --- | --- | --- | --- | --- | --- | --- | --- |
| Fri | - | Sat | 0.233 | 0.177 | 1.318 | 421 | 0.188 | 1 |
| Fri | - | Sun | 0.259 | 0.169 | 1.534 | 421 | 0.126 | 1 |
| Mon | - | Fri | -0.187 | 0.179 | -1.045 | 421 | 0.297 | 1 |
| Mon | - | Sat | 0.047 | 0.187 | 0.249 | 421 | 0.803 | 1 |
| Mon | - | Sun | 0.072 | 0.179 | 0.403 | 421 | 0.687 | 1 |
| Mon | - | Wed | -0.178 | 0.181 | -0.981 | 421 | 0.327 | 1 |
| Mon | - | Thu | -0.133 | 0.186 | -0.719 | 421 | 0.472 | 1 |
| Mon | - | Tue | 0.164 | 0.183 | 0.894 | 421 | 0.372 | 1 |
| Sat | - | Sun | 0.026 | 0.178 | 0.144 | 421 | 0.885 | 1 |
| Wed | - | Fri | -0.009 | 0.171 | -0.052 | 421 | 0.958 | 1 |
| Wed | - | Sat | 0.224 | 0.18 | 1.25 | 421 | 0.212 | 1 |
| Wed | - | Sun | 0.25 | 0.171 | 1.458 | 421 | 0.145 | 1 |
| Wed | - | Thu | 0.044 | 0.178 | 0.25 | 421 | 0.803 | 1 |
| Thu | - | Fri | -0.053 | 0.175 | -0.304 | 421 | 0.761 | 1 |
| Thu | - | Sat | 0.18 | 0.184 | 0.98 | 421 | 0.328 | 1 |
| Thu | - | Sun | 0.206 | 0.176 | 1.169 | 421 | 0.243 | 1 |
| Tue | - | Fri | -0.351 | 0.173 | -2.027 | 421 | 0.043 | 0.909 |
| Tue | - | Sat | -0.117 | 0.182 | -0.646 | 421 | 0.519 | 1 |
| Tue | - | Sun | -0.092 | 0.174 | -0.528 | 421 | 0.598 | 1 |
| Tue | - | Wed | -0.342 | 0.176 | -1.947 | 421 | 0.052 | 1 |
| Tue | - | Thu | -0.297 | 0.18 | -1.653 | 421 | 0.099 | 1 |

*[* p < 0.05; ** p < 0.01, *** p < 0.001]*

***Supplementary Table G.31:** Results of generalized linear model for Z-scored BART for those with a Weak SOW of weekday, age, and gender.

|  |  |  | **95% Confidence Interval** | | |  | |  | |  | |  |  |
| --- | --- | --- | --- | --- | --- | --- | --- | --- | --- | --- | --- | --- | --- |
| **Effect** | **Estimate** | **SE** | **Lower** | **Upper** | **β** | | **df** | | **t** | | **p** | | |
| (Intercept) | 0.072 | 0.054 | -0.034 | 0.178 | 0.000 | | 419 | | 1.337 | | 0.182 | | |
| Age | -0.006 | 0.004 | -0.014 | 0.002 | -0.071 | | 419 | | -1.455 | | 0.147 | | |
| Male - Female | 0.111 | 0.108 | -0.101 | 0.323 | 0.113 | | 419 | | 1.027 | | 0.305 | | |
| Tue - Mon | -0.139 | 0.184 | -0.500 | 0.222 | -0.141 | | 419 | | -0.756 | | 0.450 | | |
| Wed - Mon | 0.169 | 0.181 | -0.187 | 0.525 | 0.172 | | 419 | | 0.935 | | 0.350 | | |
| Thu - Mon | 0.143 | 0.185 | -0.221 | 0.508 | 0.146 | | 419 | | 0.773 | | 0.440 | | |
| Fri - Mon | 0.192 | 0.179 | -0.160 | 0.543 | 0.195 | | 419 | | 1.073 | | 0.284 | | |
| Sat - Mon | -0.036 | 0.187 | -0.404 | 0.331 | -0.037 | | 419 | | -0.193 | | 0.847 | | |
| Sun - Mon | -0.086 | 0.179 | -0.438 | 0.267 | -0.087 | | 419 | | -0.477 | | 0.633 | | |

*[* p < 0.05; ** p < 0.01, *** p < 0.001]*

***Supplementary Materials Table G.32.:** Results of post-hoc comparisons for Z-scored BART for those with a Weak SOW of weekday, age, and gender.

| **Weekday** |  |  | **Difference** | **SE** | **t** | **df** | **p** | **p_bonferroni_** |
| --- | --- | --- | --- | --- | --- | --- | --- | --- |
| Fri | - | Sat | 0.228 | 0.177 | 1.288 | 419 | 0.199 | 1 |
| Fri | - | Sun | 0.277 | 0.169 | 1.641 | 419 | 0.102 | 1 |
| Mon | - | Fri | -0.192 | 0.179 | -1.073 | 419 | 0.284 | 1 |
| Mon | - | Sat | 0.036 | 0.187 | 0.193 | 419 | 0.847 | 1 |
| Mon | - | Sun | 0.086 | 0.179 | 0.477 | 419 | 0.633 | 1 |
| Mon | - | Wed | -0.169 | 0.181 | -0.935 | 419 | 0.350 | 1 |
| Mon | - | Thu | -0.143 | 0.185 | -0.773 | 419 | 0.440 | 1 |
| Mon | - | Tue | 0.139 | 0.184 | 0.756 | 419 | 0.450 | 1 |
| Sat | - | Sun | 0.050 | 0.178 | 0.278 | 419 | 0.781 | 1 |
| Wed | - | Fri | -0.022 | 0.171 | -0.130 | 419 | 0.896 | 1 |
| Wed | - | Sat | 0.205 | 0.180 | 1.144 | 419 | 0.253 | 1 |
| Wed | - | Sun | 0.255 | 0.171 | 1.488 | 419 | 0.137 | 1 |
| Wed | - | Thu | 0.026 | 0.178 | 0.147 | 419 | 0.883 | 1 |
| Thu | - | Fri | -0.048 | 0.175 | -0.276 | 419 | 0.783 | 1 |
| Thu | - | Sat | 0.179 | 0.184 | 0.977 | 419 | 0.329 | 1 |
| Thu | - | Sun | 0.229 | 0.176 | 1.299 | 419 | 0.195 | 1 |
| Tue | - | Fri | -0.330 | 0.173 | -1.908 | 419 | 0.057 | 1 |
| Tue | - | Sat | -0.103 | 0.182 | -0.566 | 419 | 0.572 | 1 |
| Tue | - | Sun | -0.053 | 0.175 | -0.305 | 419 | 0.761 | 1 |
| Tue | - | Wed | -0.308 | 0.176 | -1.748 | 419 | 0.081 | 1 |
| Tue | - | Thu | -0.282 | 0.180 | -1.568 | 419 | 0.118 | 1 |

*[* p < 0.05; ** p < 0.01, *** p < 0.001]*

**Supplementary Material Figure J:** Comparison of risk score calculated as in main text and calculated in the same manner but without inclusion of the BART score. Error bars represent +/- SE.

**
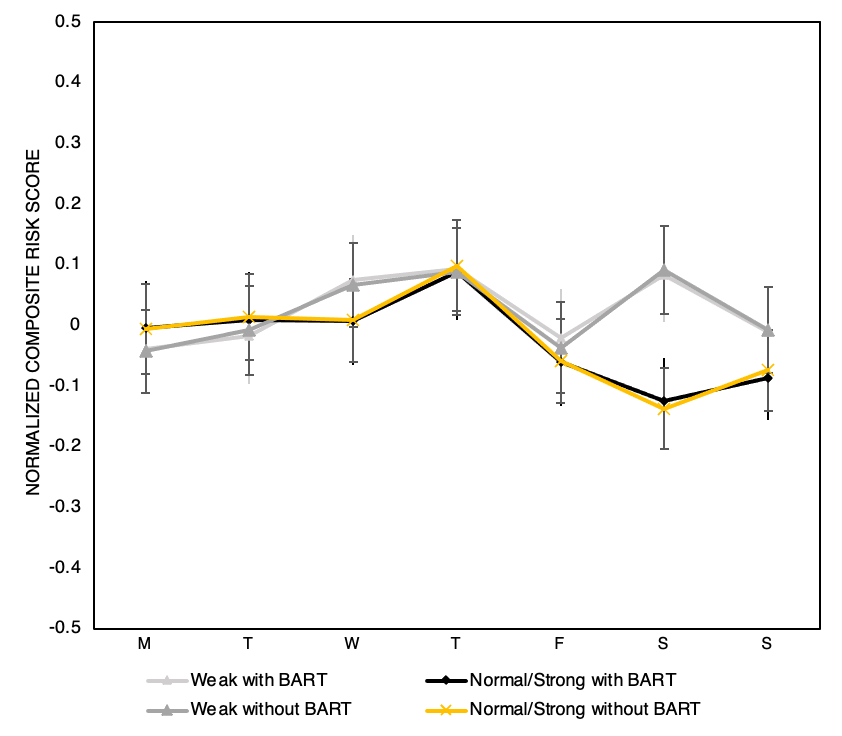
**

***Supplementary Table J.1:**  Results of generalized linear model for Z-scored composite risk score without BART for those with a Normal/Strong SOW of weekday only.

|  |  |  | **95% Confidence Interval** | | |  |  |  |
| --- | --- | --- | --- | --- | --- | --- | --- | --- |
| **Effect** | **Estimate** | **SE** | **Lower** | **Upper** | **β** | **df** | **t** | **p** |
| (Intercept) | -0.022 | 0.028 | -0.078 | 0.034 | 0 | 394 | -0.783 | 0.434 |
| Tue - Mon | 0.02 | 0.104 | -0.184 | 0.224 | 0.035 | 394 | 0.191 | 0.848 |
| Wed - Mon | 0.014 | 0.104 | -0.19 | 0.219 | 0.025 | 394 | 0.139 | 0.889 |
| Thu - Mon | 0.105 | 0.102 | -0.097 | 0.306 | 0.184 | 394 | 1.022 | 0.307 |
| Fri - Mon | -0.052 | 0.107 | -0.263 | 0.158 | -0.092 | 394 | -0.49 | 0.625 |
| Sat - Mon | -0.131 | 0.101 | -0.33 | 0.068 | -0.23 | 394 | -1.294 | 0.196 |
| Sun - Mon | -0.068 | 0.105 | -0.274 | 0.138 | -0.119 | 394 | -0.645 | 0.519 |

*[* p < 0.05; ** p < 0.01, *** p < 0.001]*

***Supplementary Materials Table J.2.:** Results of post-hoc comparisons for Z-scored composite risk score without BART for those with a Normal/Strong SOW of weekday only.

| **Weekday** |  | **Weekday** | **Difference** | **SE** | **t** | **df** | **p** | **p_bonferroni_** |
| --- | --- | --- | --- | --- | --- | --- | --- | --- |
| Fri | - | Sat | 0.078 | 0.108 | 0.727 | 394 | 0.468 | 1 |
| Fri | - | Sun | 0.015 | 0.111 | 0.137 | 394 | 0.891 | 1 |
| Mon | - | Fri | 0.052 | 0.107 | 0.49 | 394 | 0.625 | 1 |
| Mon | - | Sat | 0.131 | 0.101 | 1.294 | 394 | 0.196 | 1 |
| Mon | - | Sun | 0.068 | 0.105 | 0.645 | 394 | 0.519 | 1 |
| Mon | - | Wed | -0.014 | 0.104 | -0.139 | 394 | 0.889 | 1 |
| Mon | - | Thu | -0.105 | 0.102 | -1.022 | 394 | 0.307 | 1 |
| Mon | - | Tue | -0.02 | 0.104 | -0.191 | 394 | 0.848 | 1 |
| Sat | - | Sun | -0.063 | 0.106 | -0.598 | 394 | 0.55 | 1 |
| Wed | - | Fri | 0.067 | 0.11 | 0.606 | 394 | 0.545 | 1 |
| Wed | - | Sat | 0.145 | 0.105 | 1.389 | 394 | 0.166 | 1 |
| Wed | - | Sun | 0.082 | 0.108 | 0.759 | 394 | 0.448 | 1 |
| Wed | - | Thu | -0.09 | 0.106 | -0.852 | 394 | 0.395 | 1 |
| Thu | - | Fri | 0.157 | 0.109 | 1.441 | 394 | 0.151 | 1 |
| Thu | - | Sat | 0.235 | 0.103 | 2.282 | 394 | 0.023 | 0.483 |
| Thu | - | Sun | 0.172 | 0.107 | 1.613 | 394 | 0.108 | 1 |
| Tue | - | Fri | 0.072 | 0.11 | 0.655 | 394 | 0.513 | 1 |
| Tue | - | Sat | 0.151 | 0.105 | 1.441 | 394 | 0.151 | 1 |
| Tue | - | Sun | 0.087 | 0.108 | 0.809 | 394 | 0.419 | 1 |
| Tue | - | Wed | 0.005 | 0.107 | 0.05 | 394 | 0.96 | 1 |
| Tue | - | Thu | -0.085 | 0.106 | -0.801 | 394 | 0.423 | 1 |

*[* p < 0.05; ** p < 0.01, *** p < 0.001]*

***Supplementary Table J.3:**  Results of generalized linear model for Z-scored composite risk score without BART for those with a Normal/Strong SOW of weekday, age, and gender.

|  |  |  | **95% Confidence Interval** | |  |  |  |  |
| --- | --- | --- | --- | --- | --- | --- | --- | --- |
| **Effect** | **Estimate** | **SE** | **Lower** | **Upper** | **β** | **df** | **t** | **p** |
| (Intercept) | 0.053 | 0.029 | -0.005 | 0.110 | 0.000 | 392 | 1.810 | 0.071 |
| Age *** | -0.009 | 0.002 | -0.014 | -0.005 | -0.189 | 392 | -4.000 | < .001 |
| Male – Female *** | 0.367 | 0.059 | 0.252 | 0.482 | 0.646 | 392 | 6.265 | < .001 |
| Tue - Mon | -0.031 | 0.098 | -0.223 | 0.161 | -0.055 | 392 | -0.319 | 0.750 |
| Wed - Mon | -0.026 | 0.097 | -0.217 | 0.166 | -0.045 | 392 | -0.263 | 0.793 |
| Thu - Mon | 0.125 | 0.096 | -0.063 | 0.314 | 0.221 | 392 | 1.305 | 0.193 |
| Fri - Mon | -0.011 | 0.100 | -0.209 | 0.186 | -0.020 | 392 | -0.114 | 0.909 |
| Sat - Mon | -0.162 | 0.095 | -0.349 | 0.024 | -0.286 | 392 | -1.712 | 0.088 |
| Sun - Mon | -0.092 | 0.098 | -0.285 | 0.101 | -0.162 | 392 | -0.938 | 0.349 |

*[* p < 0.05; ** p < 0.01, *** p < 0.001]*

***Supplementary Materials Table J.4:** Results of post-hoc comparisons for Z-scored composite risk score without BART for those with a Normal/Strong SOW of weekday, age, and gender.

| **Weekday** |  |  | **Difference** | **SE** | **t** | **df** | **p** | **p_bonferroni_** |
| --- | --- | --- | --- | --- | --- | --- | --- | --- |
| Fri | - | Sat | 0.151 | 0.101 | 1.489 | 392 | 0.137 | 1.000 |
| Fri | - | Sun | 0.081 | 0.105 | 0.772 | 392 | 0.441 | 1.000 |
| Mon | - | Fri | 0.011 | 0.100 | 0.114 | 392 | 0.909 | 1.000 |
| Mon | - | Sat | 0.162 | 0.095 | 1.712 | 392 | 0.088 | 1.000 |
| Mon | - | Sun | 0.092 | 0.098 | 0.937 | 392 | 0.349 | 1.000 |
| Mon | - | Wed | 0.026 | 0.097 | 0.263 | 392 | 0.793 | 1.000 |
| Mon | - | Thu | -0.125 | 0.096 | -1.305 | 392 | 0.193 | 1.000 |
| Mon | - | Tue | 0.031 | 0.098 | 0.319 | 392 | 0.750 | 1.000 |
| Sat | - | Sun | -0.070 | 0.099 | -0.711 | 392 | 0.478 | 1.000 |
| Wed | - | Fri | -0.014 | 0.104 | -0.136 | 392 | 0.892 | 1.000 |
| Wed | - | Sat | 0.137 | 0.098 | 1.397 | 392 | 0.163 | 1.000 |
| Wed | - | Sun | 0.067 | 0.101 | 0.657 | 392 | 0.512 | 1.000 |
| Wed | - | Thu | -0.151 | 0.099 | -1.519 | 392 | 0.130 | 1.000 |
| Thu | - | Fri | 0.137 | 0.102 | 1.340 | 392 | 0.181 | 1.000 |
| Thu | - | Sat | 0.288 | 0.097 | 2.972 | 392 | 0.003 | 0.066 |
| Thu | - | Sun | 0.217 | 0.100 | 2.171 | 392 | 0.031 | 0.641 |
| Tue | - | Fri | -0.020 | 0.104 | -0.190 | 392 | 0.850 | 1.000 |
| Tue | - | Sat | 0.131 | 0.098 | 1.340 | 392 | 0.181 | 1.000 |
| Tue | - | Sun | 0.061 | 0.101 | 0.601 | 392 | 0.548 | 1.000 |
| Tue | - | Wed | -0.006 | 0.100 | -0.056 | 392 | 0.955 | 1.000 |
| Tue | - | Thu | -0.157 | 0.100 | -1.573 | 392 | 0.116 | 1.000 |

*[* p < 0.05; ** p < 0.01, *** p < 0.001]*

***Supplementary Table J.5:**  Results of generalized linear model for Z-scored composite risk score without BART for those with a Weak SOW of weekday only.

|  |  |  | **95% Confidence Interval** | | |  |  |  |
| --- | --- | --- | --- | --- | --- | --- | --- | --- |
| **Effect** | **Estimate** | **SE** | **Lower** | **Upper** | **β** | **df** | **t** | **p** |
| (Intercept) | 0.022 | 0.029 | -0.035 | 0.078 | 0 | 421 | 0.752 | 0.452 |
| Tue - Mon | 0.034 | 0.11 | -0.182 | 0.251 | 0.058 | 421 | 0.309 | 0.757 |
| Wed - Mon | 0.109 | 0.109 | -0.105 | 0.324 | 0.186 | 421 | 1.005 | 0.315 |
| Thu - Mon | 0.131 | 0.111 | -0.088 | 0.35 | 0.223 | 421 | 1.174 | 0.241 |
| Fri - Mon | 0.006 | 0.107 | -0.205 | 0.217 | 0.01 | 421 | 0.055 | 0.956 |
| Sat - Mon | 0.134 | 0.112 | -0.087 | 0.355 | 0.227 | 421 | 1.189 | 0.235 |
| Sun - Mon | 0.035 | 0.108 | -0.177 | 0.246 | 0.059 | 421 | 0.321 | 0.749 |

*[* p < 0.05; ** p < 0.01, *** p < 0.001]*

***Supplementary Materials Table J.6:** Results of post-hoc comparisons for Z-scored composite risk score without BART for those with a Weak SOW of weekday only.

| **Weekday** |  | **Weekday** | **Difference** | **SE** | **t** | **df** | **p** | **p_bonferroni_** |
| --- | --- | --- | --- | --- | --- | --- | --- | --- |
| Fri | - | Sat | -0.128 | 0.106 | -1.201 | 421 | 0.231 | 1 |
| Fri | - | Sun | -0.029 | 0.101 | -0.282 | 421 | 0.778 | 1 |
| Mon | - | Fri | -0.006 | 0.107 | -0.055 | 421 | 0.956 | 1 |
| Mon | - | Sat | -0.134 | 0.112 | -1.189 | 421 | 0.235 | 1 |
| Mon | - | Sun | -0.035 | 0.108 | -0.321 | 421 | 0.749 | 1 |
| Mon | - | Wed | -0.109 | 0.109 | -1.005 | 421 | 0.315 | 1 |
| Mon | - | Thu | -0.131 | 0.111 | -1.174 | 421 | 0.241 | 1 |
| Mon | - | Tue | -0.034 | 0.11 | -0.309 | 421 | 0.757 | 1 |
| Sat | - | Sun | 0.099 | 0.107 | 0.928 | 421 | 0.354 | 1 |
| Wed | - | Fri | 0.104 | 0.103 | 1.009 | 421 | 0.314 | 1 |
| Wed | - | Sat | -0.024 | 0.108 | -0.224 | 421 | 0.823 | 1 |
| Wed | - | Sun | 0.075 | 0.103 | 0.727 | 421 | 0.468 | 1 |
| Wed | - | Thu | -0.021 | 0.107 | -0.2 | 421 | 0.841 | 1 |
| Thu | - | Fri | 0.125 | 0.105 | 1.186 | 421 | 0.236 | 1 |
| Thu | - | Sat | -0.003 | 0.11 | -0.025 | 421 | 0.98 | 1 |
| Thu | - | Sun | 0.096 | 0.106 | 0.911 | 421 | 0.363 | 1 |
| Tue | - | Fri | 0.028 | 0.104 | 0.271 | 421 | 0.787 | 1 |
| Tue | - | Sat | -0.1 | 0.109 | -0.913 | 421 | 0.362 | 1 |
| Tue | - | Sun | -4.848e−4 | 0.104 | -0.005 | 421 | 0.996 | 1 |
| Tue | - | Wed | -0.075 | 0.105 | -0.715 | 421 | 0.475 | 1 |
| Tue | - | Thu | -0.097 | 0.108 | -0.895 | 421 | 0.371 | 1 |

*[* p < 0.05; ** p < 0.01, *** p < 0.001]*

***Supplementary Table J.7:** Results of generalized linear model for Z-scored composite risk score without BARTfor those with a Weak SOW of weekday, age, and gender.

|  |  |  | **95% Confidence Interval** | |  |  |  |  |
| --- | --- | --- | --- | --- | --- | --- | --- | --- |
| **Effect** | **Estimate** | **SE** | **Lower** | **Upper** | **β** | **df** | **t** | **p** |
| (Intercept) | 0.094 | 0.031 | 0.033 | 0.155 | 0.000 | 419 | 3.018 | 0.003 |
| Age *** | -0.009 | 0.002 | -0.013 | -0.004 | -0.172 | 419 | -3.668 | < .001 |
| Male - Female *** | 0.306 | 0.062 | 0.184 | 0.429 | 0.521 | 419 | 4.920 | < .001 |
| Tue - Mon | 0.085 | 0.106 | -0.124 | 0.293 | 0.144 | 419 | 0.799 | 0.425 |
| Wed - Mon | 0.094 | 0.105 | -0.111 | 0.300 | 0.160 | 419 | 0.902 | 0.368 |
| Thu - Mon | 0.150 | 0.107 | -0.060 | 0.361 | 0.256 | 419 | 1.405 | 0.161 |
| Fri - Mon | 0.013 | 0.103 | -0.190 | 0.215 | 0.022 | 419 | 0.123 | 0.902 |
| Sat - Mon | 0.155 | 0.108 | -0.057 | 0.368 | 0.265 | 419 | 1.440 | 0.151 |
| Sun - Mon | 0.006 | 0.104 | -0.198 | 0.209 | 0.009 | 419 | 0.054 | 0.957 |

*[* p < 0.05; ** p < 0.01, *** p < 0.001]*

***Supplementary Materials Table J.8.:** Results of post-hoc comparisons for Z-scored composite risk score for those with a Weak SOW of weekday, age, and gender.

| **Weekday** |  |  | **Difference** | **SE** | **t** | **df** | **p** | **p_bonferroni_** |
| --- | --- | --- | --- | --- | --- | --- | --- | --- |
| Fri | - | Sat | -0.143 | 0.102 | -1.398 | 419 | 0.163 | 1 |
| Fri | - | Sun | 0.007 | 0.098 | 0.073 | 419 | 0.942 | 1 |
| Mon | - | Fri | -0.013 | 0.103 | -0.123 | 419 | 0.902 | 1 |
| Mon | - | Sat | -0.155 | 0.108 | -1.440 | 419 | 0.151 | 1 |
| Mon | - | Sun | -0.006 | 0.104 | -0.054 | 419 | 0.957 | 1 |
| Mon | - | Wed | -0.094 | 0.105 | -0.902 | 419 | 0.368 | 1 |
| Mon | - | Thu | -0.150 | 0.107 | -1.405 | 419 | 0.161 | 1 |
| Mon | - | Tue | -0.085 | 0.106 | -0.799 | 419 | 0.425 | 1 |
| Sat | - | Sun | 0.150 | 0.103 | 1.459 | 419 | 0.145 | 1 |
| Wed | - | Fri | 0.082 | 0.099 | 0.828 | 419 | 0.408 | 1 |
| Wed | - | Sat | -0.061 | 0.104 | -0.590 | 419 | 0.556 | 1 |
| Wed | - | Sun | 0.089 | 0.099 | 0.897 | 419 | 0.370 | 1 |
| Wed | - | Thu | -0.056 | 0.103 | -0.546 | 419 | 0.585 | 1 |
| Thu | - | Fri | 0.138 | 0.101 | 1.362 | 419 | 0.174 | 1 |
| Thu | - | Sat | -0.005 | 0.106 | -0.048 | 419 | 0.962 | 1 |
| Thu | - | Sun | 0.145 | 0.102 | 1.423 | 419 | 0.155 | 1 |
| Tue | - | Fri | 0.072 | 0.100 | 0.720 | 419 | 0.472 | 1 |
| Tue | - | Sat | -0.071 | 0.105 | -0.675 | 419 | 0.500 | 1 |
| Tue | - | Sun | 0.079 | 0.101 | 0.784 | 419 | 0.433 | 1 |
| Tue | - | Wed | -0.010 | 0.102 | -0.094 | 419 | 0.925 | 1 |
| Tue | - | Thu | -0.066 | 0.104 | -0.632 | 419 | 0.528 | 1 |

*[* p < 0.05; ** p < 0.01, *** p < 0.001]*
